# Supplementary material for: Transition-metal-free formal cross-coupling of aryl methyl sulfoxides and alcohols via nucleophilic activation of C-S bond
Source: Nat Commun. 2020 Jun 8;11:2890. doi: 10.1038/s41467-020-16713-8 (PMC7280189; doi:10.1038/s41467-020-16713-8)
Supplement: Supplementary file 1 — Supplementary Information [file 41467_2020_16713_MOESM1_ESM.pdf]

Supplementary Information

Transition-Metal-Free Formal Cross-Coupling of Aryl Methyl Sulfoxides and Alcohols via Nucleophilic Activation of C-S Bond

Li *et al*.

Supplementary Methods

General Information

All reactions were carried out under dry argon. Anhydrous 1,4-dioxane, 2-methyltetrahydrofuran, and dimethoxyethane (DME) were purchased from J&K Chemicals, and directly used without further purification. Unless otherwise stated, reagents were commercially available and used as purchased without further purification. Chemicals were purchased from J&K Chemicals, Adamas-beta, Macklin Reagent, Energy Chemicals, Aladdin, JiuDing Chemicals or Bide Pharmatech Ltd. The progress of the reactions was monitored by thin-layer chromatography using Whatman Partisil K6F 250 μm precoated 60 Å silica gel plates and visualized by short-wave ultraviolet light as well as by treatment with iodine. Flash chromatography was performed with silica gel (300–400 mesh). The NMR spectra were obtained using a Brüker 400 MHz Fourier-transform NMR spectrometer. Chemical shifts are reported in units of parts per million (ppm) downfield from tetramethylsilane (TMS), and all coupling constants are reported in hertz. The infrared spectra were taken with KBr plates with a Perkin-Elmer Spectrum Vertex 80 Series spectrometer. High resolution mass spectrometry (HRMS) data were obtained on a Bruker Apex IV RTMS using electrospray ionization (ESI) in positive mode. Melting points were determined on a Mel-Temp melting point apparatus and were uncorrected.

Preparation of Aryl Methyl Sulfoxides

Sulfoxides were prepared according to the literature procedures[1, 2].

General Procedure for Catalysis

To an oven-dried microwave vial equipped with a stir bar was added KO^{t}Bu (44.9 mg, 0.4 mmol, 2 equiv.), and 2-methanesulfinyl-naphthalene (38.0 mg, 0.2 mmol, 1 equiv.) under argon atmosphere in a glove box. DME (0.4 mL) was added to the vial by syringe. The microwave vial was sealed and removed from the glove box. Then, methanol (16.2 μL, 0.4 mmol, 2 equiv.) was added by syringe under argon atmosphere. Note that solid and viscous oil alcohols were added to the reaction vial prior to KO^{t}Bu. The reaction mixture was heated to 110 °C in an oil bath and stirred for 12 h. Upon completion of the reaction, the sealed vial was cooled to room temperature, and opened to air. The reaction mixture was passed through a short pad of silica gel. The pad was then rinsed with 10:1 dichloromethane:methanol. The resulting solution was subjected to reduced pressure to remove the volatile materials and yielded a viscous oil. The residue was purified by flash chromatography as outlined below.

Procedure and Characterization

2-Methoxy-naphthalene (3a): The reaction was performed following the General Procedure with **1a** (38.0 mg, 0.2 mmol), methanol (**2a**) (16.2 μL, 0.4 mmol), and

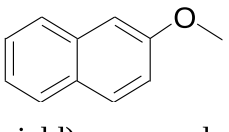

KO^{t}Bu (44.9 mg, 0.4 mmol) in 0.4 mL DME at 110 ℃ for 12 h. The crude product was purified by flash chromatography on silica gel (eluted with hexanes) to give the product **3a** (28.3 mg, 90% yield) as a colorless solid; R_f = 0.4 (hexanes); HRMS calculated for $C_{11}H_{11}O$ 159.0804, found 159.0805 $[M+H]^+$. The spectroscopic data match the previously reported data[3].

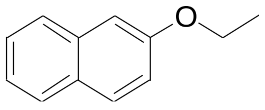

2-Ethoxy-naphthalene (3b): The reaction was performed following the General Procedure with **1a** (38.0 mg, 0.2 mmol), ethanol (**2a**) (23.4 µL, 0.4 mmol), and KO^{t}Bu (44.9 mg, 0.4 mmol) in 0.4 mL DME at 110 ℃ for 12 h. The crude product was purified by flash chromatography on silica gel (eluted with hexanes) to give the product **3b** (32.0 mg, 93% yield) as a colorless solid; R_f = 0.5 (hexanes). The spectroscopic data match the previously reported data[4].

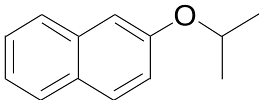

2-Isopropoxy-naphthalene (3c): The reaction was performed following the General Procedure with **1a** (38.0 mg, 0.2 mmol), propan-2-ol (**2c**) (30.6 µL, 0.4 mmol), and KO^{t}Bu (44.9 mg, 0.4 mmol) in 0.4 mL DME at 110 ℃ for 12 h. The crude product was purified by flash chromatography on silica gel (eluted with hexanes) to give the product **3c** (30.5 mg, 82% yield) as a pale yellow solid; R_f = 0.5 (hexanes). The spectroscopic data match the previously reported data[5].

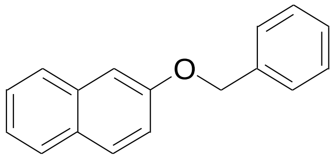

2-Benzyloxy-naphthalene (3d): The reaction was performed following the General Procedure with **1a** (38.0 mg, 0.2 mmol), phenyl-methanol (**2d**) (41.4 µL, 0.4 mmol), and KO^{t}Bu (44.9 mg, 0.4 mmol) in 0.4 mL DME at 110 ℃ for 12 h. The crude product was purified by flash chromatography on silica gel (eluted with hexanes) to give the product **3d** (28.5 mg, 61% yield) as a colorless solid; R_f = 0.2 (hexanes). The spectroscopic data match the previously reported data[6].

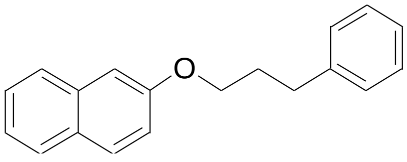

2-(3-Phenyl-propoxy)-naphthalene (3e): The reaction was performed following the General Procedure with **1a** (38.0 mg, 0.2 mmol), 3-Phenyl-propan-1-ol (**2e**) (54.4 mg, 0.4 mmol), and KO^{t}Bu (44.9 mg, 0.4 mmol) in 0.4 mL DME at 110 ℃ for 12 h. The crude product was purified by flash chromatography on silica gel (eluted with hexanes) to give the product **3e** (45.1 mg, 86% yield) as a colorless solid; R_f = 0.2 (hexanes); ^{1}H NMR (400 MHz, $CDCl_3$) δ 7.81 – 7.65 (m, 3H), 7.42 (t, J = 7.1 Hz, 1H), 7.35 – 7.26 (m, 3H), 7.25 – 7.13 (m, 4H), 7.09 (d, J = 2.2 Hz, 1H), 4.08 (t, J = 6.3 Hz, 2H), 2.86 (t, J = 7.6 Hz, 2H), 2.22 – 2.12 (m, 2H); ^{13}C NMR (100 MHz, $CDCl_3$) δ 157.1, 141.6, 134.7, 129.5, 129.0, 128.7, 128.6, 127.8, 126.8, 126.4, 126.1, 123.6, 119.1, 106.7, 66.9, 32.3, 30.9; IR (thin film): 2361, 2160, 1459, 1347, 1186, 1142, 988, 811 cm^{-1};

HRMS calculated for $C_{19}H_{19}O$ 263.1430, found 263.1430 $[M+H]^+$. The melting point was previously reported[7].

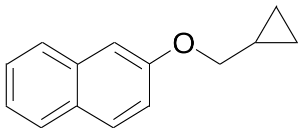

2-Cyclopropylmethoxy-naphthalene (3f): The reaction was performed following the General Procedure with **1a** (38.0 mg, 0.2 mmol), cyclopropyl-methanol (**2f**) (32.4 μL, 0.4 mmol), and KO^{t}Bu (44.9 mg, 0.4 mmol) in 0.4 mL DME at 110 °C for 12 h. The crude product was purified by flash chromatography on silica gel (eluted with hexanes) to give the product **3f** (36.7 mg, 93% yield) as a colorless solid; R_f = 0.3 (hexanes); ^{1}H NMR (400 MHz, $CDCl_3$) δ 7.81 – 7.68 (m, 3H), 7.49 – 7.39 (m, 1H), 7.38 – 7.29 (m, 1H), 7.20 (dd, *J* = 8.9, 2.5 Hz, 1H), 7.12 (d, *J* = 2.5 Hz, 1H), 3.93 (d, *J* = 6.9 Hz, 2H), 1.42 – 1.30 (m, 1H), 0.73 – 0.65 (m, 2H), 0.46 – 0.37 (m, 2H); ^{13}C NMR (100 MHz, $CDCl_3$) δ 157.1, 134.7, 129.5, 129.1, 127.8, 126.8, 126.4, 123.7, 119.2, 106.8, 72.9, 10.4, 3.4; IR (thin film): 2361, 2341, 1626, 1595, 1507, 1465, 1257, 1215, 1174, 1009, 839, 818, 742 cm^{-1}; HRMS calculated for $C_{14}H_{15}O$ 199.1117, found 199.1116 $[M+H]^+$. The melting point was previously reported[8].

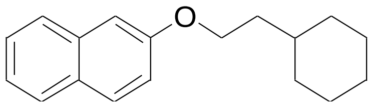

2-(2-Cyclohexyl-ethoxy)-naphthalene (3g): The reaction was performed following the General Procedure with **1a** (38.0 mg, 0.2 mmol), 2-cyclohexyl-ethanol (**2g**) (51.3 mg, 0.4 mmol), and KO^{t}Bu (44.9 mg, 0.4 mmol) in 0.4 mL DME at 110 °C for 12 h. The crude product was purified by flash chromatography on silica gel (eluted with hexanes) to give the product **3g** (44.7 mg, 88% yield) as a colorless viscous oil; R_f = 0.5 (hexanes); ^{1}H NMR (400 MHz, $CDCl_3$) δ 7.80 – 7.71 (m, 3H), 7.47 – 7.42 (m, 1H), 7.37 – 7.31 (m, 1H), 7.19 – 7.14 (m, 2H), 4.13 (t, *J* = 6.7 Hz, 2H), 1.86 – 1.67 (m, 7H), 1.62 – 1.53 (m, 1H), 1.33 – 1.18 (m, 3H), 1.08 – 0.96 (m, 2H); ^{13}C NMR (100 MHz, $CDCl_3$) δ 157.2, 134.8, 129.4, 129.0, 127.8, 126.8, 126.4, 123.6, 119.2, 106.6, 66.1, 36.8, 34.8, 33.5, 26.7, 26.4; IR (thin film): 2924, 2851, 1258, 1217, 1181, 986, 837, 746 cm^{-1}; HRMS calculated for $C_{18}H_{23}O$ 255.1743, found 255.1742 $[M+H]^+$.

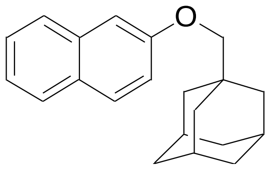

1-(Naphthalen-2-yloxymethyl)-adamantane (3h): The reaction was performed following the General Procedure with **1a** (38.0 mg, 0.2 mmol), adamantan-1-yl-methanol (**2h**) (66.5 mg, 0.4 mmol), and KO^{t}Bu (44.9 mg, 0.4 mmol) in 0.4 mL DME at 110 °C for 12 h. The crude product was purified by flash chromatography on silica gel (eluted with hexanes) to give the product **3h** (49.7 mg, 80% yield) as a colorless solid; R_f = 0.6 (hexanes); m.p. = 135–136 °C; ^{1}H NMR (400 MHz, $CDCl_3$) δ 7.80 – 7.71 (m, 3H), 7.44 (t, *J* = 7.5 Hz, 1H), 7.33 (dd, *J* = 7.9, 7.0 Hz, 1H), 7.19 (dd, *J* = 8.9, 1.5 Hz, 1H), 7.14 (d, *J* = 1.5 Hz, 1H), 3.65 (s, 2H), 2.07 (s, 3H), 1.85 – 1.71 (m, 12H); ^{13}C NMR (100 MHz, $CDCl_3$) δ 157.8, 134.8, 129.3, 128.9, 127.7, 126.8, 126.4, 123.5, 119.3, 106.6, 78.4, 39.7, 37.3, 33.9, 28.4; IR

(thin film): 2902, 1627, 1599, 1260, 1218, 1185, 1031, 834, 812, 745 cm^{-1}; HRMS calculated for $C_{21}H_{25}O$ 293.1900, found 293.1898 $[M+H]^+$.

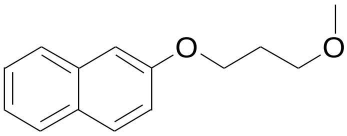

2-(3-Methoxy-propoxy)-naphthalene (3i): The reaction was performed following the General Procedure with **1a** (38.0 mg, 0.2 mmol), 3-methoxy-propan-1-ol (**2i**) (38.3 μL, 0.4 mmol), and KO^{t}Bu (44.9 mg, 0.4 mmol) in 0.4 mL DME at 110 ℃ for 12 h. The crude product was purified by flash chromatography on silica gel (eluted with hexanes) to give the product **3i** (40.2 mg, 93% yield) as a colorless viscous oil; R_f = 0.2 (hexanes). The spectroscopic data match the previously reported data[9].

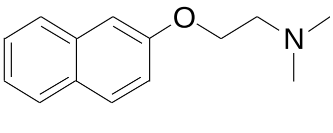

Dimethyl-[2-(naphthalen-2-yloxy)-ethyl]-amine (3j): The reaction was performed following the General Procedure with **1a** (38.0 mg, 0.2 mmol), 2-dimethylamino-ethanol (**2j**) (40.2 μL, 0.4 mmol), and KO^{t}Bu (44.9 mg, 0.4 mmol) in 0.4 mL DME at 110 ℃ for 12 h. The crude product was purified by flash chromatography on silica gel (eluted with EtOAc:methanol = 20:1) to give the product **3j** (43.0 mg, 99% yield) as a dark brown viscous oil; R_f = 0.1 (EtOAc:methanol); ^{1}H NMR (400 MHz, $CDCl_3$) δ 7.81 – 7.69 (m, 3H), 7.47 – 7.40 (m, 1H), 7.37 – 7.30 (m, 1H), 7.20 (dd, *J* = 8.9, 2.5 Hz, 1H), 7.15 (d, *J* = 2.5 Hz, 1H), 4.20 (t, *J* = 5.7 Hz, 2H), 2.81 (t, *J* = 5.7 Hz, 2H), 2.38 (s, 6H); ^{13}C NMR (100 MHz, $CDCl_3$) δ 157.0, 134.6, 129.5, 129.1, 127.8, 126.9, 126.4, 123.7, 119.2, 106.7, 66.1, 58.4, 46.1; IR (thin film): 1630, 1268, 1182, 971, 839, 747 cm^{-1}; HRMS calculated for $C_{14}H_{18}NO$ 216.1383, found 216.1382 $[M+H]^+$.

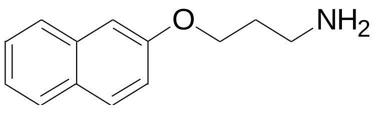

3-(Naphthalen-2-yloxy)-propylamine (3k): The reaction was performed following the General Procedure with **1a** (38.0 mg, 0.2 mmol), 3-amino-propan-1-ol (**2k**) (30.6 μL, 0.4 mmol), and KO^{t}Bu (44.9 mg, 0.4 mmol) in 0.4 mL DME, at 110 ℃ for 12 h. The crude product was purified by flash chromatography on silica gel (eluted with EtOAc:methanol = 10:1) to give the product **3k** (37.4 mg, 93% yield) as a pale yellow solid; R_f = 0.6 (EtOAc:methanol = 2:1); m.p. = 103–104 ℃; ^{1}H NMR (400 MHz, $CDCl_3$) δ 7.82 – 7.66 (m, 3H), 7.47 – 7.39 (m, 1H), 7.37 – 7.30 (m, 1H), 7.15 (s, 1H), 4.17 (t, *J* = 5.7 Hz, 2H), 2.96 (t, *J* = 6.2 Hz, 2H), 2.05 – 1.92 (m, 2H), 1.52 (br s, 2H); ^{13}C NMR (100 MHz, $CDCl_3$) δ 157.0, 134.7, 129.5, 129.3, 127.8, 126.8, 126.5, 123.7, 119.0, 106.7, 66.0, 39.4, 33.1; IR (thin film): 2922, 1598, 1461, 1391, 1257, 1216, 1182, 1029, 838, 816, 745 cm^{-1}; HRMS calculated for $C_{13}H_{16}NO$ 202.1226, found 202.1226 $[M+H]^+$.

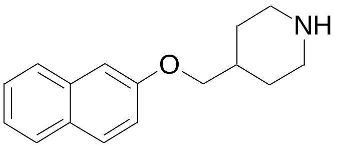

4-(Naphthalen-2-yloxymethyl)-piperidine (3l): The reaction was performed following the General Procedure with **1a** (38.0 mg, 0.2 mmol), piperidin-4-yl-methanol (**2l**) (46.1 mg, 0.4 mmol), and KO^{t}Bu (44.9 mg, 0.4 mmol) in

0.4 mL DME at 110 ℃ for 12 h. The crude product was purified by flash chromatography on silica gel (eluted with EtOAc:methanol = 10:1) to give the product **3l** (49.7 mg, 99% yield) as a pale yellow solid; R_f = 0.6 (EtOAc:methanol = 2:1); m.p. = 88–89 ℃; ^{1}H NMR (400 MHz, $CDCl_3$) δ 7.80 – 7.67 (m, 3H), 7.43 (t, *J* = 7.4 Hz, 1H), 7.33 (t, *J* = 7.4 Hz, 1H), 7.20 – 7.05 (m, 2H), 3.90 (d, *J* = 6.2 Hz, 1H), 3.16 (d, *J* = 11.7 Hz, 2H), 2.97 (s, 1H), 2.68 (t, *J* = 11.7 Hz, 2H), 2.06 – 1.93 (m, 1H), 1.88 (d, *J* = 12.4 Hz, 1H), 1.45 – 1.28 (m, 2H); ^{13}C NMR (100 MHz, $CDCl_3$) δ 157.1, 134.6, 129.4, 129.0, 127.7, 126.7, 126.4, 123.6, 119.0, 106.6, 72.9, 46.1, 36.3, 30.0; IR (thin film): 1257, 1216, 1182, 1029, 838, 816, 745 cm^{-1}; HRMS calculated for $C_{16}H_{20}NO$ 242.1539, found 242.1538 $[M+H]^+$.

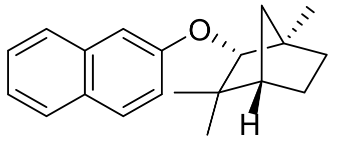

2-(1,3,3-Trimethyl-bicyclo[2.2.1]hept-2-yloxy)-naphthalene (3m): The reaction was performed following the General Procedure with **1a** (38.0 mg, 0.2 mmol), 1,3,3-trimethyl-bicyclo[2.2.1]heptan-2-ol (**2m**) (66.3 μL, 0.4 mmol), and KO^{t}Bu (44.9 mg, 0.4 mmol) in 0.4 mL DME at 110 ℃ for 12 h. The crude product was purified by flash chromatography on silica gel (eluted with hexanes) to give the product **3m** (56.0 mg, 99% yield) as a colorless solid; R_f = 0.7 (hexanes); m.p. = 70–71 ℃; ^{1}H NMR (400 MHz, $CDCl_3$) δ 7.81 – 7.69 (m, 3H), 7.47 – 7.42 (m, 1H), 7.37 – 7.31 (m, 1H), 7.25 – 7.18 (m, 2H), 4.09 (d, *J* = 1.0 Hz, 1H), 2.18 – 2.09 (m, 1H), 1.89 – 1.78 (m, 2H), 1.69 (dd, *J* = 10.3, 1.5 Hz, 1H), 1.59 – 1.50 (m, 1H), 1.33 – 1.28 (m, 4H), 1.21 – 1.13 (m, 4H), 0.93 (s, 3H); ^{13}C NMR (100 MHz, $CDCl_3$) δ 158.3, 134.7, 129.4, 128.9, 127.7, 126.7, 126.4, 123.5, 119.8, 108.4, 90.1, 49.8, 49.3, 41.6, 40.3, 30.8, 26.6, 26.1, 20.5, 20.1; IR (thin film): 2949, 2923, 1596, 1460, 1252, 1213, 1173, 1058, 834, 816, 743 cm^{-1}; HRMS calculated for $C_{20}H_{25}O$ 281.1900, found 281.1897 $[M+H]^+$.

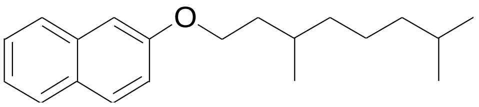

2-(3,7-Dimethyl-octyloxy)-naphthalene (3n): The reaction was performed following the General Procedure with **1a** (38.0 mg, 0.2 mmol), 3,7-dimethyl-octan-1-ol (**2n**) (76.5 μL, 0.4 mmol), and KO^{t}Bu (44.9 mg, 0.4 mmol) in 0.4 mL DME at 110 ℃ for 12 h. The crude product was purified by flash chromatography on silica gel (eluted with hexanes) to give the product **3n** (50.6 mg, 89% yield) as a colorless viscous oil; R_f = 0.6 (hexanes); ^{1}H NMR (400 MHz, $CDCl_3$) δ 7.81 – 7.71 (m, 3H), 7.48 – 7.41 (m, 1H), 7.37 – 7.30 (m, 1H), 7.20 – 7.12 (m, 2H), 4.18 – 4.08 (m, 2H), 1.97 – 1.87 (m, 1H), 1.80 – 1.61 (m, 2H), 1.60 – 1.51 (m, 1H), 1.42 – 1.28 (m, 3H), 1.26 – 1.16 (m, 3H), 1.00 (d, *J* = 6.5 Hz, 3H), 0.90 (d, *J* = 6.5 Hz, 6H); ^{13}C NMR (100 MHz, $CDCl_3$) δ 157.3, 134.8, 129.4, 129.0, 127.8, 126.8, 126.4, 123.6, 119.2, 106.6, 66.5, 39.4, 37.5, 36.3, 30.1, 28.2, 24.8, 22.8, 19.9; IR (thin film): 2955, 1631, 1602, 1467, 1258, 1217, 1181, 837, 810, 746 cm^{-1}; HRMS calculated for $C_{20}H_{29}O$ 285.2213, found 285.2212 $[M+H]^+$.

(2-Cyclohexyl-ethoxy)-benzene (4a): The reaction was performed following the General Procedure with methanesulfinyl-benzene (**1aa)** (28.0 mg, 0.2 mmol),

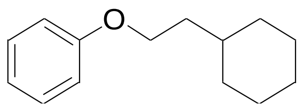

2-cyclohexyl-ethanol (**2g**) (76.9 mg, 0.6 mmol), and KO^{t}Bu (67.3 mg, 0.6 mmol) in 0.4 mL DME at 110 ℃ for 24 h. The crude product was purified by flash chromatography on silica gel (eluted with hexanes) to give the product **4a** (77.5 mg, 76% yield) as a colorless solid; R$_f$ = 0.6 (hexanes); m.p. = 34–35 ℃; ^{1}H NMR (400 MHz, $CDCl_3$) δ 7.33 – 7.26 (m, 2H), 6.98 – 6.88 (m, 3H), 4.01 (t, J = 6.7 Hz, 2H), 1.82 – 1.66 (m, 7H), 1.59 – 1.47 (m, 1H), 1.34 – 1.16 (m, 3H), 1.06 – 0.93 (m, 2H); ^{13}C NMR (100 MHz, $CDCl_3$) δ 159.3, 129.5, 120.6, 114.6, 77.5, 77.2, 76.8, 65.9, 36.8, 34.7, 33.5, 26.7, 26.4; IR (thin film): 2924, 2381, 1182, 1244, 1029, 753, 691 cm^{-1}; HRMS calculated for $C_{14}H_{21}O$ 205.1592, found 205.1588 [M+H]$^+$.

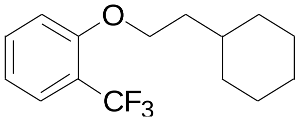

1-(2-Cyclohexyl-ethoxy)-2-trifluoromethyl-benzene (4b): The reaction was performed following the General Procedure with 1-methanesulfinyl-2-trifluoromethyl-benzene (**1b)** (41.6 mg, 0.2 mmol), 2-cyclohexyl-ethanol (**2g**) (51.3 mg, 0.4 mmol), and KO^{t}Bu (44.9 mg, 0.4 mmol) in 0.4 mL DME at 110 ℃ for 12 h. The crude product was purified by flash chromatography on silica gel (eluted with hexanes) to give the product **4b** (54.4 mg, 99% yield) as a colorless viscous oil; R$_f$ = 0.7 (hexanes); ^{1}H NMR (400 MHz, $CDCl_3$) δ 7.61 – 7.53 (m, 1H), 7.50 – 7.42 (m, 1H), 7.03 – 6.94 (m, 2H), 4.08 (t, J = 6.6 Hz, 2H), 1.84 – 1.62 (m, 7H), 1.62 – 1.49 (m, 1H), 1.32 – 1.13 (m, 3H), 1.03 – 0.91 (m, 2H); ^{13}C NMR (100 MHz, $CDCl_3$) δ 157.2, 133.3, 127.2 (q, J_{C-F} = 5.3 Hz), 123.9 (q, J_{C-F} = 272.3 Hz), 119.8, 119.0 (q, J_{C-F} = 30.7 Hz), 112.8, 66.8, 36.5, 34.5, 33.3, 26.7, 26.4; IR (thin film): 1636, 1460, 1182, 1323, 1135, 1059, 1034, 755 cm^{-1}; HRMS calculated for $C_{15}H_{19}F_3ONa$ 295.1280, found 295.1277 [M+Na]$^+$.

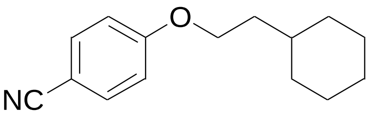

4-(2-Cyclohexyl-ethoxy)-benzonitrile (4c): The reaction was performed following the General Procedure with 4-methanesulfinyl-benzonitrile (**1c)** (33.0 mg, 0.2 mmol), 2-cyclohexyl-ethanol (**2g**) (51.3 mg, 0.4 mmol), KO^{t}Bu (44.9 mg, 0.4 mmol) in 0.4 mL DME at 110 ℃ for 12 h. The crude product was purified by flash chromatography on silica gel (eluted with hexanes:EtOAc = 10:1) to give the product **4c** (40.0 mg, 85% yield) as a pale yellow solid; R$_f$ = 0.6 (hexanes:EtOAc = 5:1); m.p. = 39–40 ℃; ^{1}H NMR (400 MHz, $CDCl_3$) δ 7.58 – 7.53 (m, 2H), 6.95 – 6.89 (m, 2H), 4.02 (t, J = 6.7 Hz, 2H), 1.77 – 1.63 (m, 7H), 1.54 – 1.43 (m, 1H), 1.30 – 1.13 (m, 3H), 1.02 – 0.91 (m, 2H); ^{13}C NMR (100 MHz, $CDCl_3$) δ 162.5, 134.0, 119.4, 115.3, 103.7, 66.5, 36.4, 34.5, 33.3, 26.5, 26.3; IR (thin film): 2926, 1460, 1606, 1509, 1259, 1172, 985, 838 cm^{-1}; HRMS calculated for $C_{15}H_{20}NO$ 230.1539, found 230.1537 [M+H]$^+$.

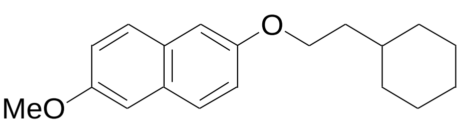

2-(2-Cyclohexyl-ethoxy)-6-methoxy-naphthalene (4d): The reaction was performed following the General Procedure with **1d** (44.0 mg, 0.2 mmol), 2-cyclohexyl-ethanol (**2g**) (76.9 mg, 0.6

mmol), and KO^{t}Bu (67.3 mg, 0.6 mmol) in 0.4 mL DME at 110 ℃ for 24 h. The crude product was purified by flash chromatography on silica gel (eluted with hexanes:DCM = 7:1) to give the product **4d** (31.8 mg, 56% yield) as a colorless solid; R$_f$ = 0.5 (hexanes:EtOAc = 40:1); m.p. = 100–102 ℃; ^{1}H NMR (400 MHz, $CDCl_3$) δ 7.65 – 7.59 (m, 2H), 7.15 – 7.07 (m, 4H), 4.08 (t, J = 6.7 Hz, 2H), 3.90 (s, 3H), 1.83 – 1.64 (m, 7H), 1.59 – 1.52 (m, 1H), 1.31 – 1.16 (m, 3H), 1.06 – 0.95 (m, 2H); ^{13}C NMR (100 MHz, $CDCl_3$) δ 156.1, 155.8, 129.9, 129.8, 128.2, 128.2, 119.4, 119.0, 107.1, 106.2, 66.2, 55.5, 36.8, 34.8, 33.5, 26.7, 26.4; IR (thin film): 2924, 2850, 1601, 1506, 1453, 1393, 1229, 1162, 1113, 1081, 850, 801 cm^{-1}; HRMS calculated for $C_{19}H_{25}O_2$ 285.1849, found 285.1848 [M+H]$^+$.

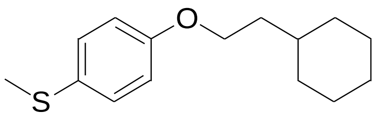

1-(2-Cyclohexyl-ethoxy)-4-methylsulfanyl-benzene (4e): The reaction was performed following the General Procedure with 1-methanesulfinyl-4-methylsulfanyl-benzene (**1e)** (18.6 mg, 0.1 mmol), 2-cyclohexyl-ethanol (**2g**) (38.5 mg, 0.3 mmol), and KO^{t}Bu (33.6 mg, 0.3 mmol) in 0.2 mL DME at 110 ℃ for 12 h. The crude product was purified by flash chromatography on silica gel (eluted with hexanes) to give the product **4e** (15.1 mg, 69% yield) as a colorless solid; R$_f$ = 0.4 (hexanes); m.p. = 49–50 ℃; ^{1}H NMR (400 MHz, $CDCl_3$) δ 7.32 (d, J = 7.8 Hz, 2H), 6.90 (d, J = 7.9 Hz, 2H), 4.03 (t, J = 6.4 Hz, 2H), 2.50 (s, 3H), 1.83 – 1.70 (m, 7H), 1.58 – 1.53 (m, 1H), 1.37 – 1.19 (m, 3H), 1.08 – 0.96 (m, 2H); ^{13}C NMR (100 MHz, $CDCl_3$) δ 157.9, 130.3, 128.5, 115.3, 66.2, 36.7, 34.6, 33.4, 26.6, 26.3, 18.3; IR (thin film): 2916, 2849, 1592, 1494, 1477, 1282, 1239, 1182, 1018, 826, 812, 797, 664 cm^{-1}; HRMS calculated for $C_{15}H_{22}OS$ 251.1464, found 251.1467 [M+H]$^+$.

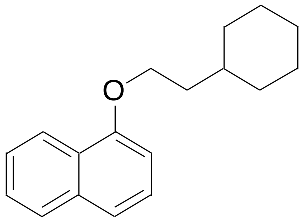

1-(2-Cyclohexyl-ethoxy)-naphthalene (4f): The reaction was performed following the General Procedure with 1-methanesulfinyl-naphthalene (**1f)** (38.0 mg, 0.2 mmol), 2-cyclohexyl-ethanol (**2g**) (51.3 mg, 0.4 mmol), and KO^{t}Bu (44.9 mg, 0.4 mmol) in 0.4 mL DME at 110 ℃ for 12 h. The crude product was purified by flash chromatography on silica gel (eluted with hexanes) to give the product **4f** (47.8 mg, 94% yield) as a pale yellow solid; R$_f$ = 0.6 (hexanes); m.p. = 53–54 ℃; ^{1}H NMR (400 MHz, $CDCl_3$) δ 8.34 – 8.27 (m, 1H), 7.84 – 7.77 (m, 1H), 7.55 – 7.45 (m, 2H), 7.45 – 7.35 (m, 2H), 6.82 (d, J = 7.3 Hz, 1H), 4.19 (t, J = 6.5 Hz, 2H), 1.90 – 1.81 (m, 4H), 1.79 – 1.59 (m, 4H), 1.36 – 1.15 (m, 3H), 1.11 – 0.99 (m, 2H); ^{13}C NMR (100 MHz, $CDCl_3$) δ 155.1, 134.6, 127.5, 126.4, 126.1, 125.9, 125.2, 122.3, 120.0, 104.6, 66.3, 36.8, 35.0, 33.5, 26.7, 26.4; IR (thin film): 2918, 2850, 2360, 1386, 1265, 1238, 1098, 1067, 991, 789, 768, 732 cm^{-1}; HRMS calculated for $C_{18}H_{23}O$ 255.1743, found 255.1742 [M+H]$^+$.

1-(2-Cyclohexyl-ethoxy)-3-vinyl-benzene (4g): The reaction was performed following the General Procedure with 1-methanesulfinyl-3-vinyl-benzene (**1g)** (28.8 mg, 0.2 mmol), 2-cyclohexyl-ethanol (**2g**) (51.3 mg, 0.4 mmol), and KO^{t}Bu (44.9 mg, 0.4 mmol) in 0.4 mL DME at 110 ℃ for 12 h. The crude product was purified by

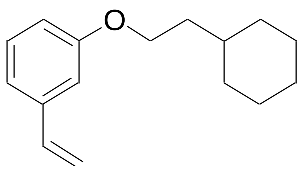

flash chromatography on silica gel (eluted with hexanes) to give the product **4g** (26.1 mg, 57% yield) as a colorless viscous oil; R_f = 0.4 (hexanes); ^{1}H NMR (400 MHz, $CDCl_3$) δ 7.23 (t, *J* = 7.9 Hz, 1H), 7.01 – 6.93 (m, 2H), 6.83 – 6.78 (m, 1H), 6.69 (dd, *J* = 17.6, 10.9 Hz, 1H), 5.74 (dd, *J* = 17.6, 0.8 Hz, 1H), 5.24 (dd, *J* = 10.9, 0.8 Hz, 1H), 4.01 (t, *J* = 6.7 Hz, 2H), 1.81 – 1.65 (m, 7H), 1.57 – 1.46 (m, 1H), 1.31 – 1.18 (m, 3H), 1.04 – 0.92 (m, 2H); ^{13}C NMR (100 MHz, $CDCl_3$) δ 159.5, 139.1, 137.0, 129.6, 118.9, 114.1, 112.3, 66.0, 36.8, 34.7, 33.5, 26.7, 26.4; IR (thin film): 3736, 3055, 2926, 2854, 2361, 1577, 1260, 1558, 1067, 989, 788, 670 cm^{-1}; HRMS calculated for $C_{16}H_{23}O$ 231.1743, found 231.1742 [M+H]$^+$.

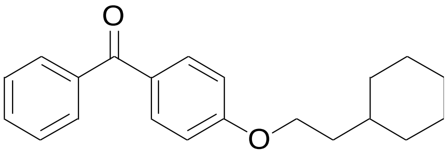

4-(2-Cyclohexyl-ethoxy)-phenyl]-phenyl-methanone (4h): The reaction was performed following the General Procedure with (4-methanesulfinyl-phenyl)-phenyl-methanone (**1h)** (48.8 mg, 0.2 mmol), 2-cyclohexyl-ethanol (**2g**) (51.3 mg, 0.4 mmol), and KO^{t}Bu (44.9 mg, 0.4 mmol) in 0.4 mL DME at 110 ℃ for 12 h. The crude product was purified by flash chromatography on silica gel (eluted with hexanes:EtOAc = 5:1) to give the product **4h** (35.2 mg, 57% yield) as a pale yellow solid; R_f = 0.6 (hexanes:EtOAc = 5:1); m.p. = 73–74 ℃; ^{1}H NMR (400 MHz, $CDCl_3$) δ 7.85 – 7.79 (m, 2H), 7.78 – 7.72 (m, 2H), 7.59 – 7.53 (m, 1H), 7.47 (t, *J* = 7.5 Hz, 1H), 6.98 – 6.91 (m, 2H), 4.08 (t, *J* = 6.7 Hz, 2H), 1.81 – 1.64 (m, 7H), 1.58 – 1.46 (m, 1H), 1.30 – 1.15 (m, 3H), 1.04 – 0.92 (m, 2H); ^{13}C NMR (100 MHz, $CDCl_3$) δ 195.7, 163.0, 138.5, 132.7, 132.0, 130.0, 129.8, 128.3, 114.1, 66.4, 36.6, 34.6, 33.4, 26.6, 26.4; IR (thin film): 2921, 2850, 2300, 1598, 1253, 1171, 842, 793, 696 cm^{-1}; HRMS calculated for $C_{21}H_{25}O_2$ 309.1849, found 309.1849 [M+H]$^+$.

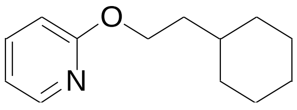

2-(2-Cyclohexyl-ethoxy)-pyridine (4i): The reaction was performed following the General Procedure with 2-methanesulfinyl-pyridine (**1i)** (28.2 mg, 0.2 mmol), 2-cyclohexyl-ethanol (**2g**) (51.3 mg, 0.4 mmol), and KO^{t}Bu (44.9 mg, 0.4 mmol) in 0.4 mL DME at 110 ℃ for 12 h. The crude product was purified by flash chromatography on silica gel (eluted with hexanes:EtOAc = 10:1) to give the product **4i** (41.0 mg, 99% yield) as a viscous oil; R_f = 0.1 (hexanes); ^{1}H NMR (400 MHz, $CDCl_3$) δ 8.16 – 8.12 (m, 1H), 7.57 – 7.51 (m, 1H), 6.85 – 6.80 (m, 1H), 6.73 – 6.69 (m, 1H), 4.31 (t, *J* = 6.9 Hz, 2H), 1.81 – 1.73 (m, 2H), 1.73 – 1.62 (m, 5H), 1.53 – 1.43 (m, 1H), 1.30 – 1.13 (m, 3H), 1.02 – 0.91 (m, 2H); ^{13}C NMR (100 MHz, $CDCl_3$) δ 164.2, 147.0, 138.5, 116.5, 111.2, 64.2, 36.6, 34.8, 33.5, 26.7, 26.4; IR (thin film): 2923, 1595, 1454, 1288, 1004, 780 cm^{-1}; HRMS calculated for $C_{13}H_{20}NO$ 206.1539, found 206.1539 [M+H]$^+$.

2-(2-Cyclohexyl-ethoxy)-6-trifluoromethyl-pyridine (4j): The reaction was performed following the General Procedure with 2-methanesulfinyl-

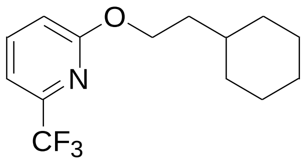

6-trifluoromethyl- pyridine (**1j**) (41.8 mg, 0.2 mmol), 2-cyclohexyl-ethanol (**2g**) (51.3 mg, 0.4 mmol), and KO^{t}Bu (44.9 mg, 0.4 mmol) in 0.4 mL DME at 110 ℃ for 12 h. The crude product was purified by flash chromatography on silica gel (eluted with hexanes) to give the product **4j** (43.7 mg, 80% yield) as a colorless viscous oil; R$_f$ = 0.5 (hexanes); ^{1}H NMR (400 MHz, $CDCl_3$) δ 7.71 – 7.63 (m, 1H), 7.22 (d, *J* = 7.3 Hz, 1H), 6.88 (d, *J* = 8.4 Hz, 1H), 4.38 (t, *J* = 6.8 Hz, 2H), 1.82 – 1.62 (m, 7H), 1.53 – 1.41 (m, 1H), 1.31 – 1.14 (m, 1H), 1.03 – 0.91 (m, 1H); ^{13}C NMR (100 MHz, $CDCl_3$) δ 164.2, 145.7 (q, $J^2_{C\text{-}F}$ = 34.5 Hz), 139.3, 121.6 (q, $J^1_{C\text{-}F}$ = 273.7 Hz), 114.7, 113.0 (q, $J^3_{C\text{-}F}$ = 3.2 Hz), 64.8, 36.4, 34.7, 33.5, 26.7, 26.4; IR (thin film): 2925, 2361, 1607, 1459, 1348, 1287, 1187, 1145, 988, 813 cm^{-1}; HRMS calculated for $C_{14}H_{19}F_3NO$ 274.1413, found 274.1413 $[M+H]^+$.

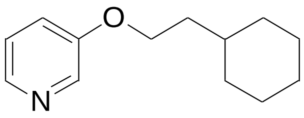

3-(2-Cyclohexyl-ethoxy)-pyridine (4k): The reaction was performed following the General Procedure with 3-methanesulfinyl-pyridine (**1k**) (28.2 mg, 0.2 mmol), 2-cyclohexyl-ethanol (**2g**) (51.3 mg, 0.4 mmol), and KO^{t}Bu (44.9 mg, 0.4 mmol) in 0.4 mL DME at 110 ℃ for 12 h. The crude product was purified by flash chromatography on silica gel (eluted with hexanes:EtOAc = 10:1) to give the product **4k** (36.4 mg, 88% yield) as a pale yellow solid; R$_f$ = 0.1 (hexanes); m.p. = 57–58 ℃; ^{1}H NMR (400 MHz, $CDCl_3$) δ 8.32 – 8.27 (m, 1H), 8.21 – 8.17 (m, 1H), 7.22 – 7.15 (m, 2H), 4.03 (t, *J* = 6.7 Hz, 2H), 1.78 – 1.66 (m, 7H), 1.55 – 1.45 (m, 1H), 1.29 – 1.14 (m, 3H), 1.03 – 0.91 (m, 2H); ^{13}C NMR (100 MHz, $CDCl_3$) δ 155.4, 142.0, 138.2, 123.9, 121.2, 66.4, 36.6, 34.6, 33.4, 26.6, 26.4; IR (thin film): 2925, 2853, 1577, 1283, 1235, 984, 707 cm^{-1}; HRMS calculated for $C_{18}H_{23}O$ 206.1539, found 206.1537 $[M+H]^+$.

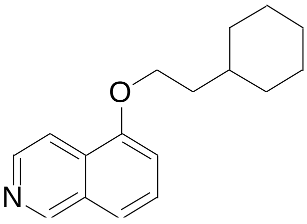

5-(2-Cyclohexyl-ethoxy)-isoquinoline (4l): The reaction was performed following the General Procedure with 5-methanesulfinyl-isoquinoline (**1l**) (38.2 mg, 0.2 mmol), 2-cyclohexyl-ethanol (**2g**) (51.3 mg, 0.4 mmol), and KO^{t}Bu (44.9 mg, 0.4 mmol) in 0.4 mL DME at 110 ℃ for 12 h. The crude product was purified by flash chromatography on silica gel (eluted with hexanes:EtOAc = 5:1) to give the product **4l** (40.8 mg, 80% yield) as a colorless solid; R$_f$ = 0.3 (hexanes:EtOAc = 5:1); m.p. = 53–54 ℃; ^{1}H NMR (400 MHz, $CDCl_3$) δ 9.19 (s, 1H), 8.52 (d, *J* = 5.8 Hz, 2H), 8.02 (d, *J* = 5.8 Hz, 1H), 7.53 – 7.44 (m, 2H), 6.97 (dd, *J* = 7.0, 1.5 Hz, 1H), 4.16 (t, *J* = 6.5 Hz, 2H), 1.88 – 1.77 (m, 4H), 1.77 – 1.54 (m, 4H), 1.33 – 1.16 (m, 3H), 1.09 – 0.97 (m, 2H); ^{13}C NMR (100 MHz, $CDCl_3$) δ 154.1, 151.9, 142.7, 129.6, 128.7, 127.5, 119.1, 115.3, 108.4, 66.5, 36.6, 34.9, 33.5, 26.6, 26.4; IR (thin film): 2924, 1589, 1392, 1279, 1250, 1112, 982, 831, 749 cm^{-1}; HRMS calculated for $C_{17}H_{22}NO$ 256.1696, found 256.1692 $[M+H]^+$.

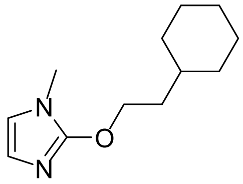

2-(2-Cyclohexyl-ethoxy)-1-methyl-1*H*-imidazole (4m): The reaction was performed following the General Procedure with 2-methanesulfinyl-1-methyl-1*H*-imidazole (**1m)** (28.8 mg, 0.2 mmol), 2-cyclohexyl-ethanol (**2g**) (51.3 mg, 0.4 mmol), and KO^{t}Bu (44.9 mg, 0.4 mmol) in 0.4 mL DME at 110 ℃ for 12 h. The crude product was purified by flash chromatography on silica gel (eluted with hexanes:EtOAc = 1:1) to give the product **4m** (27.5 mg, 66% yield) as a viscous oil; R_f = 0.1 (hexanes:EtOAc = 1:1); ^{1}H NMR (400 MHz, $CDCl_3$) δ 6.57 (d, *J* = 1.6 Hz, 1H), 6.46 (d, *J* = 1.6 Hz, 1H), 4.33 (t, *J* = 6.8 Hz, 2H), 3.36 (s, 3H), 1.81 – 1.59 (m, 7H), 1.49 – 1.38 (m, 1H), 1.26 – 1.11 (m, 3H), 1.00 – 0.88 (m, 2H); ^{13}C NMR (100 MHz, $CDCl_3$) δ 153.0, 122.7, 115.9, 67.9, 36.6, 34.7, 33.3, 30.5, 26.6, 26.3; IR (thin film): 2360, 2342, 2176, 2033, 1734, 1717, 1653, 1558, 1542, 1522, 1508, 1457, 1036, 716, 682, 631, 614 cm^{-1}; HRMS calculated for $C_{12}H_{21}N_2O$ 209.1648, found 209.1647 $[M+H]^+$.

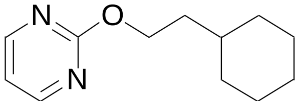

2-(2-Cyclohexyl-ethoxy)-pyrimidine (4n): The reaction was performed following the General Procedure with 2-methanesulfinyl-pyrimidine (**1n)** (28.4 mg, 0.1 mmol), 2-cyclohexyl-ethanol (**2g**) (25.6 mg, 0.2 mmol), and KO^{t}Bu (22.4 mg, 0.2 mmol) in 0.2 mL DME at 40 ℃ for 12 h. The crude product was purified by flash chromatography on silica gel (eluted with hexanes:EtOAc = 10:1) to give the product **4n** (30.0 mg, 73% yield) as a viscous oil; R_f = 0.5 (hexanes:EtOAc = 5:1); ^{1}H NMR (400 MHz, $CDCl_3$) δ 8.49 (d, *J* = 4.8 Hz, 2H), 6.89 (t, *J* = 4.8 Hz, 1H), 4.37 (t, *J* = 6.8 Hz, 2H), 1.79 – 1.60 (m, 7H), 1.58 – 1.48 (m, 1H), 1.27 – 1.11 (m, 3H), 1.01 – 0.90 (m, 2H); ^{13}C NMR (100 MHz, $CDCl_3$) δ 165.5, 159.2, 114.7, 65.8, 36.2, 34.4, 33.3, 26.6, 26.3; IR (thin film): 2921, 2850, 1578, 1562, 1472, 1380, 1323, 965, 808, 669 cm^{-1}; HRMS calculated for $C_{12}H_{18}N_2O$ 207.1492, found 207.1492 $[M+H]^+$.

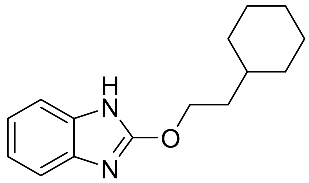

2-(2-Cyclohexyl-ethoxy)-1H-benzoimidazole (4o): The reaction was performed following the General Procedure with 2-methanesulfinyl-1H-benzoimidazole (**1o)** (18.0 mg, 0.1 mmol), 2-cyclohexyl-ethanol (**2g**) (38.5 mg, 0.3 mmol), and KO^{t}Bu (44.8 mg, 0.4 mmol) in 0.2 mL DME at 100 ℃ for 30 h. The crude product was purified by flash chromatography on silica gel (eluted with hexanes:EtOAc = 10:1) to give the product **4o** (17.0 mg, 70% yield) as a colorless solid; R_f = 0.6 (hexanes:EtOAc = 5:1); m.p. = 183–187 ℃; ^{1}H NMR (400 MHz, $CDCl_3$) δ 8.97 (s, 1H), 7.54 (d, *J* = 7.4 Hz, 1H), 7.24 – 7.05 (m, 3H), 4.56 (t, *J* = 6.8 Hz, 2H), 1.81 – 1.63 (m, 7H), 1.51 – 1.40 (m, 1H), 1.27 – 1.12 (m, 3H), 1.01 – 0.91 (m, 2H); ^{13}C NMR (100 MHz, $CDCl_3$) δ 158.3, 141.2, 132.0, 121.7, 121.4, 117.6, 109.5, 68.5, 36.4, 34.6, 33.3, 26.5, 26.3; IR (thin film): 2917, 2849, 1494, 1478, 1282, 1239, 1182, 1122, 1051, 1018, 1008, 965, 826, 797, 696, 664 cm^{-1}; HRMS calculated for $C_{15}H_{20}N_2O$ 245.1648, found 245.1650 $[M+H]^+$.

5-(2-Cyclohexyl-ethoxy)-quinoline (4p): The reaction was performed following the

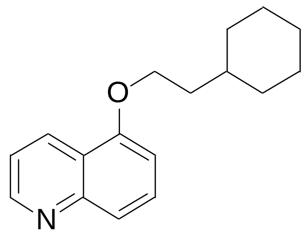

General Procedure with 5-methanesulfinyl-quinoline (**1p)** (38.2 mg, 0.2 mmol), 2-cyclohexyl-ethanol (**2g**) (51.3 mg, 0.4 mmol), and KO^{t}Bu (44.9 mg, 0.4 mmol) in 0.4 mL DME at 110 °C for 12 h. The crude product was purified by flash chromatography on silica gel (eluted with hexanes:EtOAc = 5:1) to give the product **4p** (50.5 mg, 99% yield) as a colorless solid; R_f = 0.3 (hexanes:EtOAc = 5:1); m.p. = 68–69 °C; ^{1}H NMR (400 MHz, $CDCl_3$) δ 8.89 (m, 1H), 8.59 (dd, *J* = 8.4, 1.1 Hz, 1H), 7.67 (m, 1H), 7.62 – 7.56 (m, 1H), 7.37 (dd, *J* = 8.4, 4.2 Hz, 1H), 6.84 (d, *J* = 7.6 Hz, 1H), 4.17 (t, *J* = 6.6 Hz, 2H), 1.87 – 1.77 (m, 4H), 1.76 – 1.64 (m, 3H), 1.63 – 1.54 (m, 1H), 1.33 – 1.15 (m, 3H), 1.09 – 0.97 (m, 2H); ^{13}C NMR (100 MHz, $CDCl_3$) δ 154.8, 150.7, 149.3, 131.1, 129.6, 121.4, 121.1, 120.2, 105.0, 66.6, 36.7, 35.0, 33.5, 26.6, 26.4; IR (thin film): 2920, 1629, 1587, 1464, 1388, 1258, 1204, 1170, 1089, 1009, 839, 819, 791, 742 cm^{-1}; HRMS calculated for $C_{17}H_{22}NO$ 256.1696, found 256.1691 $[M+H]^+$.

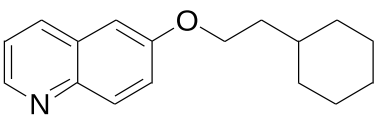

6-(2-Cyclohexyl-ethoxy)-quinoline (4q): The reaction was performed following the General Procedure with 6-methanesulfinyl-quinoline (**1q)** (38.2 mg, 0.2 mmol), 2-cyclohexyl-ethanol (**2g**) (51.3 mg, 0.4 mmol), and KO^{t}Bu (44.9 mg, 0.4 mmol) in 0.4 mL DME at 110 °C for 12 h. The crude product was purified by flash chromatography on silica gel (eluted with hexanes:EtOAc = 5:1) to give the product **4q** (46.4 mg, 91% yield) as a viscous oil; R_f = 0.3 (hexanes:EtOAc = 5:1); ^{1}H NMR (400 MHz, $CDCl_3$) δ 8.74 (dd, *J* = 4.2, 1.5 Hz, 1H), 8.04 – 7.95 (m, 2H), 7.39 – 7.29 (m, 2H), 7.04 (d, *J* = 2.7 Hz, 1H), 4.09 (t, *J* = 6.7 Hz, 2H), 1.83 – 1.63 (m, 7H), 1.60 – 1.47 (m, 1H), 1.31 – 1.14 (m, 3H), 1.05 – 0.93 (m, 2H); ^{13}C NMR (100 MHz, $CDCl_3$) δ 157.4, 147.9, 144.4, 134.8, 130.9, 129.5, 122.7, 121.4, 105.9, 66.4, 36.6, 34.7, 33.4, 26.6, 26.4; IR (thin film): 2925, 1628, 1504, 1230, 1010, 835 cm^{-1}; HRMS calculated for $C_{17}H_{22}NO$ 256.1696, found 256.1691 $[M+H]^+$.

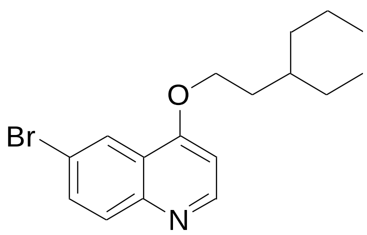

6-Bromo-4-(2-cyclohexyl-ethoxy)-quinoline (4r): The reaction was performed following the General Procedure with 6-bromo-4-methanesulfinyl-quinoline (**1r)** (26.9 mg, 0.1 mmol), 2-cyclohexyl-ethanol (**2g**) (25.6 mg, 0.2 mmol), and KO^{t}Bu (22.4 mg, 0.2 mmol) in 0.2 mL DME at 40 °C for 12 h. The crude product was purified by flash chromatography on silica gel (eluted with hexanes:EtOAc = 5:1) to give the product **4r** (28.6 mg, 86% yield) as a pale yellow solid; R_f = 0.2 (hexanes:EtOAc = 5:1); m.p. = 104–106 °C; ^{1}H NMR (400 MHz, $CDCl_3$) δ 8.72 (d, *J* = 3.2 Hz, 1H), 8.34 (s, 1H), 7.90 – 7.87 (m, 1H), 7.75 (d, *J* = 8.9 Hz, 1H), 6.73 (d, *J* = 3.3 Hz, 1H), 4.22 (t, *J* = 5.8 Hz, 2H), 1.87 – 1.67 (m, 7H), 1.63 – 1.52 (m, 1H), 1.33 – 1.20 (m, 3H), 1.10 – 0.97 (m, 2H); ^{13}C NMR (100 MHz, $CDCl_3$) δ 160.9, 151.8, 147.9, 133.2, 130.8, 124.5, 122.8, 119.5, 101.4, 67.0, 36.2, 34.7, 33.3, 26.5, 26.3; IR (thin film): 2923, 2844, 2342, 1583, 567, 1495, 1450, 1350, 1305, 1117, 973, 840, 669 cm^{-1}; HRMS calculated for $C_{17}H_{20}BrNO$ 334.0801, found 334.0800 $[M+H]^+$.

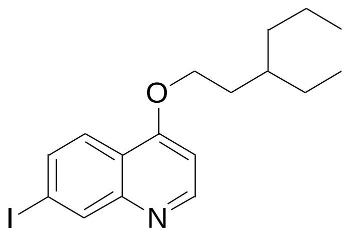

4-(2-Cyclohexyl-ethoxy)-7-iodo-quinoline (4s): The reaction was performed following the General Procedure with 7-iodo-4-methanesulfinyl-quinoline (**1s**) (31.7 mg, 0.1 mmol), 2-cyclohexyl-ethanol (**2g**) (25.6 mg, 0.2 mmol), and KO^{t}Bu (22.4 mg, 0.2 mmol) in 0.2 mL DME at 40 ℃ for 12 h. The crude product was purified by flash chromatography on silica gel (eluted with hexanes:EtOAc = 7:1) to give the product **4s** (35.1 mg, 92% yield) as a yellow solid; R_f = 0.3 (hexanes:EtOAc = 5:1); m.p. = 105–106 ℃; ^{1}H NMR (400 MHz, $CDCl_3$) δ 8.72 – 8.70 (m, 1H), 8.55 (s, 1H), 7.91 (d, *J* = 8.9 Hz, 1H), 7.73 (d, *J* = 8.9 Hz, 1H), 6.71 (d, *J* = 5.1 Hz, 1H), 4.20 (t, *J* = 6.6 Hz, 2H), 1.87 – 1.66 (m, 7H), 1.61 – 1.53 (m, 1H), 1.34 – 1.17 (m, 3H), 1.09 – 0.97 (m, 2H); ^{13}C NMR (100 MHz, $CDCl_3$) δ 160.6, 152.0, 148.2, 138.5, 131.2, 130.8, 123.3, 101.3, 90.9, 67.0, 36.2, 34.7, 33.3, 26.5, 26.3; IR (thin film): 2921, 2845, 2359, 2342, 1579, 1488, 1470, 1442, 1350, 1301, 1114, 981, 829, 731 cm^{-1}; HRMS calculated for $C_{17}H_{20}INO$ 382.0662, found 382.0658 $[M+H]^+$.

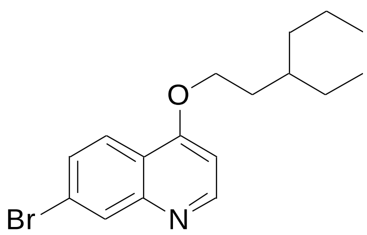

7-Bromo-4-(2-cyclohexyl-ethoxy)-quinoline (4t): The reaction was performed following the General Procedure with 7-bromo-4-methanesulfinyl-quinoline (**1t**) (26.9 mg, 0.1 mmol), 2-cyclohexyl-ethanol (**2g**) (25.6 mg, 0.2 mmol), and KO^{t}Bu (22.4 mg, 0.2 mmol) in 0.2 mL DME at 40 ℃ for 12 h. The crude product was purified by flash chromatography on silica gel (eluted with hexanes:EtOAc = 10:1) to give the product **4t** (32.2 mg, 96% yield) as a colorless solid; R_f = 0.5 (hexanes:EtOAc = 5:1); m.p. = 104–105 ℃; ^{1}H NMR (400 MHz, $CDCl_3$) δ 8.70 (d, *J* = 5.2 Hz, 1H), 8.19 (d, *J* = 1.9 Hz, 1H), 8.06 (d, *J* = 8.9 Hz, 1H), 7.58 – 7.55 (m, 1H), 6.71 (d, *J* = 5.3 Hz, 1H), 4.21 (t, *J* = 6.6 Hz, 2H), 1.86 – 1.65 (m, 7H), 1.61 – 1.54 (m, 1H), 1.31 – 1.18 (m, 3H), 1.08 – 0.98 (m, 2H); ^{13}C NMR (100 MHz, $CDCl_3$) δ 161.9, 152.5, 150.0, 131.2, 129.0, 124.1, 123.7, 120.4, 101.1, 66.9, 36.2, 34.8, 33.4, 26.5, 26.3; IR (thin film): 2930, 2916, 2842, 1565, 1427, 1374, 1306, 1117, 1008, 981, 881, 837, 814, 798, 634 cm^{-1}; HRMS calculated for $C_{17}H_{20}BrNO$ 334.0801, found 334.0797 $[M+H]^+$.

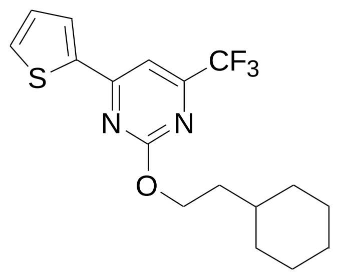

2-(2-Cyclohexyl-ethoxy)-4-thiophen-2-yl-6-trifluoromethyl-pyrimidine (4u): The reaction was performed following the General Procedure with 2-methanesulfinyl-4-thiophen-2-yl-6-trifluoromethyl-pyrimidine (**1u**) (116.8 mg, 0.4 mmol), 2-cyclohexyl-ethanol (**2g**) (12.8 mg, 0.1 mmol), and KO^{t}Bu (22.4 mg, 0.2 mmol) in 0.4 mL DME at 40 ℃ for 48 h. The crude product was purified by flash chromatography on silica gel (eluted with hexanes) to give the product **4u** (26.0 mg, 72% yield) as a viscous oil; R_f = 0.1 (hexanes); ^{1}H NMR (400 MHz, $CDCl_3$) δ 7.84 (d, *J* = 3.7 Hz, 1H), 7.58 (d, *J* = 4.9 Hz, 1H), 7.46 (s, 1H), 7.17

(m, 1H), 4.50 (t, J = 6.8 Hz, 2H), 1.83 – 1.61 (m, 7H), 1.59 – 1.48 (m, 1H), 1.32 – 1.11 (m, 3H), 1.04 – 0.92 (m, 2H); ^{13}C NMR (100 MHz, $CDCl_3$) δ 165.8, 163.7, 158.0 (q, J_{C-F} = 35.8 Hz), 141.2, 131.7, 129.2, 128.7, 120.4 (q, J_{C-F} = 275.3 Hz), 104.6 (q, J_{C-F} = 2.6 Hz), 66.7, 36.2, 34.2, 33.3, 26.6, 26.3; IR (thin film): 2923, 2852, 2362, 2343, 1591, 1441, 1336, 1265, 1194, 1157, 712 cm^{-1}; HRMS calculated for $C_{17}H_{20}F_3N_2OS$ 357.1243, found 357.1244 $[M+H]^+$.

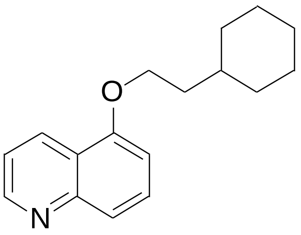

5-(2-Cyclohexyl-ethoxy)-quinoline (4p) (Gram scale): To an oven-dried 50 mL Schlenk flask equipped with a stir bar was added KO^{t}Bu (1.23 g, 11.0 mmol, 2 equiv) and 5-methanesulfinyl-quinoline (**1p**) (1.05 g, 5.5 mmol, 1 equiv). Then the flask was evacuated and back-filled with argon (this process was repeated three times). To the above flask was added 2-cyclohexyl-ethanol (**2g**) (1.41 g, 11.0 mmol, 2 equiv) and DME (11.0 mL) in sequence via syringe. The reaction mixture was heated to 110 ℃ in an oil bath and stirred for 12 h. Upon completion of the reaction, the flask was cooled to room temperature, opened to air, and the reaction mixture was passed through a short pad of silica gel. The pad was then rinsed with 10:1 dichloromethane:methanol. The resulting solution was subjected to reduced pressure to remove the volatile materials and yielded a viscous oil. The residue was purified by flash chromatography on silica gel (eluted with hexanes:EtOAc = 5:1) to give the product **4p** (1.48 g, 99% yield) as a pale yellow solid.

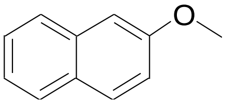

2-Methoxy-naphthalene (3a) (from 2-ethanesulfinyl-naphthalene): The reaction was performed following the General Procedure with 2-ethanesulfinyl-naphthalene (**5a**) (40.8 mg, 0.2 mmol), and methanol (**2a**) (16.2 μL, 0.4 mmol), KO^{t}Bu (44.9 mg, 0.4 mmol) in 0.4 mL DME at 110 ℃ for 12 h. 45% yield of product **3a** was detected by ^{1}H NMR by using 1.0 equiv. of CH_2Br_2 as an internal standard.

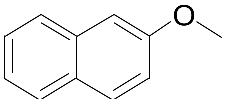

2-Methoxy-naphthalene (3a) (from 2-(2-methyl-propane-2-sulfinyl)-naphthalene): The reaction was performed following the General Procedure with 2-(2-methyl-propane-2-sulfinyl)-naphthalene (**5b**) (46.4 mg, 0.2 mmol), and methanol (**2a**) (16.2 μL, 0.4 mmol), KO^{t}Bu (44.9 mg, 0.4 mmol) in 0.4 mL DME at 110 ℃ for 12 h. Trace amount of product **3a** was detected by ^{1}H NMR by using 1.0 equiv. of CH_2Br_2 as an internal standard.

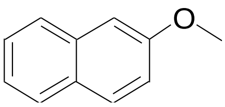

2-Methoxy-naphthalene (3a) (from 2-phenylmethanesulfinyl-naphthalene): The reaction was performed following the General Procedure with 2-phenylmethanesulfinyl-naphthalene (**5c**) (50.4 mg, 0.2 mmol), and methanol (**2a**) (16.2 μL, 0.4 mmol), KO^{t}Bu (44.9 mg, 0.4 mmol) in 0.4 mL DME at 110 ℃ for 12 h. 6% yield of product **3a** was detected by ^{1}H NMR by using 1.0 equiv. of CH_2Br_2 as an internal standard.

Synthetic applications

Synthesis of 6-(1,3,3-Trimethyl-bicyclo[2.2.1]hept-2-yloxy)-naphthalene-2-carbonitrile (6b)

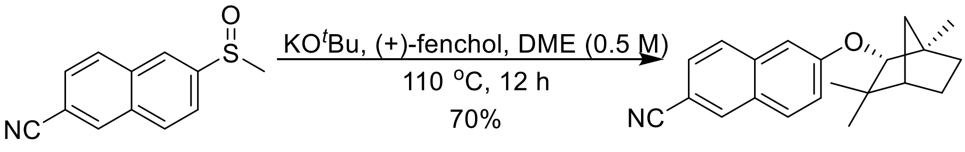

The reaction was performed following the General Procedure with 6-methanesulfinyl-naphthalene-2-carbonitrile (**6a)** (43.0 mg, 0.2 mmol), 1,3,3-trimethyl-bicyclo[2.2.1]heptan-2-ol (**2m**) (33.9 mg, 0.22 mmol), and KO^{t}Bu (24.7 mg, 0.22 mmol) in 0.4 mL DME at 110 ℃ for 12 h. The crude product was purified by flash chromatography on silica gel (eluted with hexanes) to give the product **6b** (42.7 mg, 70% yield) as a viscous oil; R_f = 0.7 (hexanes:EtOAc = 5:1); ^{1}H NMR (400 MHz, $CDCl_3$) δ 8.10 (s, 1H), 7.76 – 7.73 (d, *J* = 16.0, 1H), 7.74 – 7.70 (d, *J* = 15.4 Hz,1H), 7.54 – 7.51 (m, 1H), 7.28 –7.25 (m, 1H), 7.16 –7.15 (m, 1H), 4.08 (d, *J* = 15.6 Hz, 1H), 2.10 – 2.00 (m, 1H), 1.83 – 1.74 (m, 2H), 1.65 (dd, *J* = 10.4, 1.4 Hz, 1H), 1.57 – 1.45 (m, 1H), 1.27 – 1.25 (m, 4H), 1.14 – 1.12 (m, 4H), 0.85 (s, 3H); ^{13}C NMR (100 MHz, $CDCl_3$) δ 160.7, 136.5, 133.8, 130.0, 127.7, 127.6, 127.0, 121.5, 119.8, 108.2, 106.5, 90.3, 49.7, 49.2, 41.5, 40.3, 30.7, 26.5, 25.9, 20.4, 19.9; IR (thin film): 2966, 2361, 2341, 2227, 1624, 1613, 1600, 1476, 1391, 1266, 1229, 1163, 1101, 854 cm^{-1}; HRMS calculated for $C_{21}H_{24}NO$ 306.1852, found 306.1851 $[M+H]^+$.

Synthesis of 6-(1,3,3-Trimethyl-bicyclo[2.2.1]hept-2-yloxy)-naphthalene-2-carboxylic acid (6c)

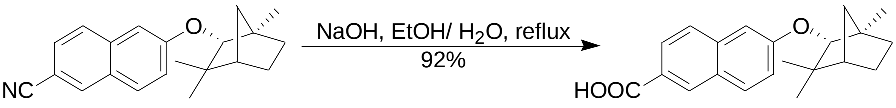

To a Schlenk flask charged with a stir bar was added **6b** (92.0 mg, 0.3 mmol), NaOH (133.2 mg, 3.3 mmol), 0.6 mL water and 0.9 mL EtOH. The mixture was then heated to reflux and stirred for 10 h. The reaction was monitored by TLC till the substrate was fully consumed. The mixture was then cooled down to room temperature. To the above mixture was added 2.0 mL 1 N HCl and then diluted with 10 mL water. The mixture was extracted with dichloromethane (3x10 mL). The combined organic phase was washed with water and brine in sequence and dried over Na_2SO_4. The solvent was concentrated to obtain the product **6c** (93.8 mg, 92% yield) as a yellow solid; R_f = 0.4 (DCM:MeOH = 50:1); m.p. = 165–167 ℃; ^{1}H NMR (400 MHz, $CDCl_3$) δ 8.62 (s, 1H), 8.08 –8.05 (m, 1H), 7.86 (d, *J* = 9.0 Hz, 1H), 7.73 (d, *J* = 8.7 Hz, 1H), 7.26 –7.23 (m, 1H), 7.19 –7.18 (m, 1H), 4.09 (d, *J* = 0.7 Hz, 1H), 2.13 – 2.03 (m, 1H), 1.85 – 1.76 (m, 2H), 1.70 – 1.64 (m, 1H), 1.57 – 1.46 (m, 1H), 1.29 – 1.25 (m, 4H), 1.14 – 1.11 (m, 4H), 0.87 (s, 3H); ^{13}C NMR (100 MHz, $CDCl_3$) δ 172.8, 160.6, 137.8, 132.0, 131.2, 127.7, 126.9, 126.1, 124.2, 120.6, 108.1, 90.2, 49.7, 49.3,

41.6, 40.3, 30.7, 26.5, 25.9, 20.4, 20.0; IR (thin film): 2956, 2925, 2871, 2855, 2360, 1676, 1621, 1475, 1416, 1388, 1286, 1267, 1257, 1204, 1162, 1130, 1046, 1010, 939, 915, 855, 823, 810, 769, 751, 734 cm^{-1}; HRMS calculated for $C_{21}H_{25}O_3$ 325.1798, found 325.1802 $[M+H]^+$.

Synthesis of [6-(1,3,3-Trimethyl-bicyclo[2.2.1]hept-2-yloxy)-naphthalen-2-yl]-methanol (6d)

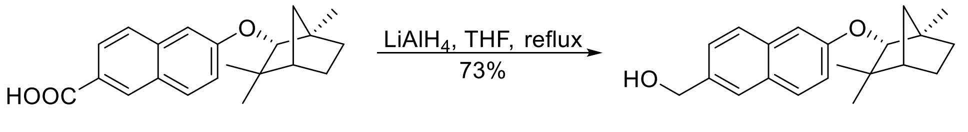

6c **6d**

To a Schlenk flask charged with a stir bar was added **6c** (68.0 mg, 0.2 mmol), and THF (2.0 mL, 0.1 M). To the above solution was added $LiAlH_4$ (30.4 mg, 0.8 mmol) at 0 °C. Then the mixture was heated to reflux for 6 h. The reaction was monitored by TLC till the substrate was fully consumed. Then the mixture was cooled down to room temperature. To the above mixture was added 1.0 mL 1 N HCl slowly. The mixture was extracted with dichloromethane (3x10 mL). The combined organic phase was washed with brine and dried over Na_2SO_4. The solvent was concentrated to obtain a residue which was further purified by flash chromatography on silica gel (eluted with petroleum ether: EtOAc =4:1) to give the product **6d** (45.2 mg, 73% yield) as a colorless soild; R_f = 0.4 (petroleum ether: EtOAc = 2:1); m.p. = 95–97 ℃; ^{1}H NMR (600 MHz, $CDCl_3$) δ 7.73 – 7.67 (m, 3H), 7.44 – 7.41 (m, 1H), 7.22 – 7.20 (m, 1H), 7.16 (d, *J* = 2.0 Hz, 1H), 4.80 (s, 2H), 4.05 (s, 1H), 2.12 – 2.05 (m, 1H), 1.83 – 1.77 (m, 2H), 1.68 – 1.63 (m, 1H), 1.55 – 1.46 (m, 1H), 1.27 – 1.24 (m, 4H), 1.16 – 1.12 (m, 4H), 0.88 (s, 3H); ^{13}C NMR (150 MHz, $CDCl_3$) δ 158.4, 135.9, 134.2, 129.3, 128.6, 127.2, 125.8, 125.6, 120.0, 108.4, 90.1, 65.7, 49.7, 49.3, 41.6, 40.2, 30.7, 26.5, 26.0, 20.4, 20.0; IR (thin film): 2949, 2923, 2869, 1633, 1605, 1581, 1460, 1418, 1389, 1364, 1262, 1239, 1216, 1174, 1162, 1048, 1009, 931, 898, 850, 818 cm^{-1}; HRMS calculated for $C_{21}H_{27}O_2$ 311.2006, found 311.2006 $[M+H]^+$.

Synthesis of [6-(1,3,3-Trimethyl-bicyclo[2.2.1]hept-2-yloxy)-naphthalen-2-yl]-methanol (6e)

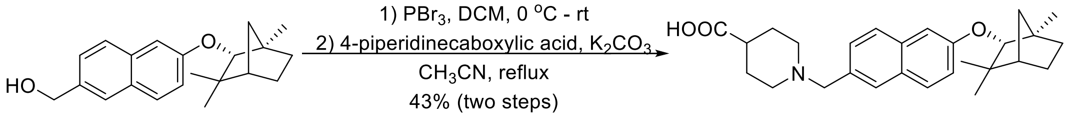

6d **6e**

To a Schlenk flask charged with a stir bar was added **6d** (93.0 mg, 0.3 mmol), and DCM (6.0 mL, 0.05 M). To the above solution was added PBr_3 (162.5 mg, 0.6 mmol) at 0 °C. Then the mixture was stirring for 3 h. The reaction was monitored by TLC till the substrate was fully consumed. Then the mixture was diluted with 10.0 mL water and extracted with dichloromethane (3x10 mL). The combined organic phase was washed with brine and dried over Na_2SO_4. The solvent was removed under vacuum. The residue was used directly for the next step without further purification.

To a Schlenk flash charged with a stir bar was added the above residue, piperidine-4-carboxylic acid (77.5 mg, 0.6 mmol), K_2CO_3 (124.4 mg, 0.9 mmol), and CH_3CN (6.0 mL, 0.05 M). The mixture was then heated to reflux and kept stirred for 12 h. The solvent was removed under vacuum. The crude product was purified by flash chromatography on silica gel (eluted with DCM:MeOH = 20:1) to give the product **6e** (54.4 mg, 43% yield) as a colorless solid; R_f = 0.4 (DCM:MeOH = 10:1); m.p. = 100–102 ℃; ^{1}H NMR (600 MHz, $CDCl_3$) δ 7.85 (s, 1H), 7.71 – 7.62 (m, 2H), 7.57 (d, J = 8.3 Hz, 1H), 7.16 (d, J = 9.0 Hz, 1H), 7.11 (s, 1H), 4.23 (s, 2H), 4.01 (s, 1H), 3.30 (s, 1H), 2.88 (s, 1H), 2.47 (s, 1H), 2.11 – 2.04 (m, 4H), 2.01 (s, 1H), 1.74 (s, 2H), 1.63 (d, J = 10.2 Hz, 1H), 1.53 – 1.44 (m, 1H), 1.25 – 1.18 (m, 7H) 1.09 (s, 3H), 0.82 (s, 3H); ^{13}C NMR (150 MHz, $CDCl_3$) δ 177.3, 159.2, 135.0, 131.1, 129.7, 128.4, 128.3, 127.7, 123.8, 120.6, 108.1, 90.1, 60.7, 49.6, 49.2, 41.5, 40.2, 34.5, 30.7, 29.4, 26.5, 25.9, 25.4, 20.4, 20.0; IR (thin film): 3820, 3750, 3735, 3696, 3587, 2954, 2925, 2871, 2359, 2341, 1732, 1717, 1699, 1636, 1606, 1480, 1457, 1436, 1395, 1264, 1236, 1221, 1179, 1161, 1122, 1050, 1010, 994, 916, 851 cm^{-1}; HRMS calculated for $C_{27}H_{36}NO_3$ 422.2690, found 422.2692 $[M+H]^+$.

Synthesis of 3-Phenoxy-propylamine (7a)

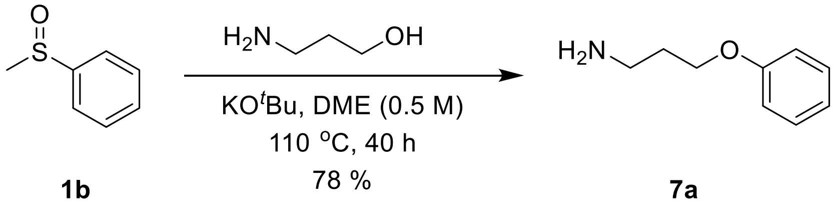

1b **7a**

The reaction was performed following the General Procedure with methanesulfinyl-benzene (**1b)** (140.0 mg, 1.0 mmol), 3-amino-propan-1-ol (**2k**) (225.3 mg, 3.0 mmol), and KO^{t}Bu (336.7 mg, 3.0 mmol) in 2.0 mL DME at 110 ℃ for 40 h. The crude product was purified by flash chromatography on silica gel (eluted with EtOAc:methanol = 7:1) to give the product **7a** (118.5 mg, 78% yield) as a viscous oil; R_f = 0.1 (EtOAc:methanol = 10:1). The spectroscopic data match the previously reported data.[10]

Synthesis of (5-Nitro-pyridin-2-yl)-(3-phenoxy-propyl)-amine (7b)

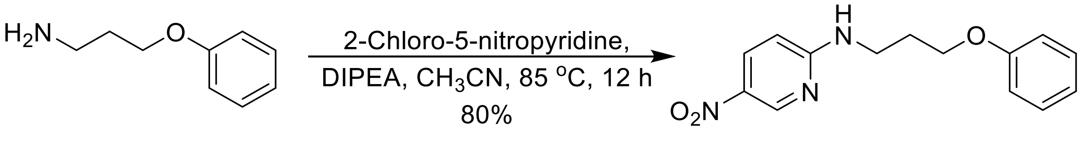

7a **7b**

To a Schlenk flask charged with a stir bar was added 3-phenoxy-propylamine (**7a**) (0.2 mmol, 31.2 mg), 2-Chloro-5-nitropyridine (0.24 mmol, 38.0 mg), DIPEA (0.4 mmol, 51.7 mg) and CH_3CN (2.0 mL, 0.1 M). The mixture was then heated to 85 ^{o}C and kept stirring for 12 h. The solvent was removed under vacuum. The crude product was purified by flash chromatography on silica gel (eluted with DCM) to give the product **7b** (44.0 mg, 80% yield) as a yellow-green solid; R_f = 0.5 (DCM); m.p. =

110–111 ℃; ^{1}H NMR (400 MHz, $CDCl_3$) δ 9.00 (d, *J* = 2.5 Hz, 1H), 8.17 – 8.15 (m, 1H), 7.32 – 7.28 (m, 2H), 6.97 (t, *J* = 7.4 Hz, 1H), 6.91 (d, *J* = 7.9 Hz, 2H), 6.37 (d, *J* = 9.3 Hz, 1H), 5.78 (br s, 1H), 4.11 (t, *J* = 5.6 Hz, 2H), 3.66 (d, *J* = 5.3 Hz, 2H), 2.20 – 2.10 (m, 2H); ^{13}C NMR (100 MHz, $CDCl_3$) δ 161.3, 158.5, 147.1, 135.8, 133.0, 129.6, 121.2, 114.5, 65.8, 40.0, 28.7; IR (thin film): 3361, 2923, 2871, 1601, 1544, 1496, 1466, 1322, 1274, 1239, 1104, 1081, 1063, 1001, 965, 950, 803, 760, 728, 689, 667 cm^{-1}; HRMS calculated for $C_{14}H_{16}N_3O_3$ 274.1186, found 274.1185 $[M+H]^+$.

Synthesis of *N*^{2}-(3-Phenoxy-propyl)-pyridine-2,5-diamine (7c)

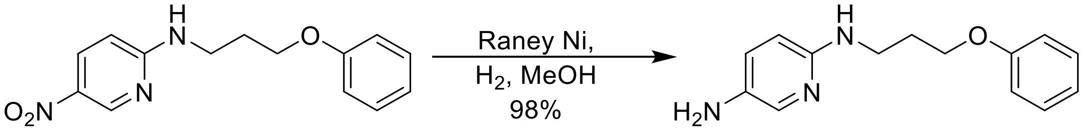

7b **7c**

To a Schlenk tube charged with a stir bar was added (**7b)** (0.1 mmol, 27.3 mg), 10% Raney Ni (2.7 mg) and MeOH (1.0 mL, 0.1 M). The tube was degassed with hydrogen for three times and then kept stirring under a H_2 balloon for 6 h. The reaction was monitored by TLC till the substrate was fully consumed. The mixture was diluted with 10 mL dichloromethane and then filtrated. The filtration was concentrated to yield a dark residue which was further purified by flash chromatography on basic aluminum oxide (eluted with DCM) to give the product **7c** (23.8 mg, 98% yield) as a viscous oil; R_f = 0.5 (DCM); ^{1}H NMR (400 MHz, $CDCl_3$) δ 7.68 (d, *J* = 2.8 Hz, 1H), 7.30 – 7.26 (m, 1H), 6.98 – 6.87 (m, 4H), 6.33 (d, *J* = 8.7 Hz, 1H), 4.32 (br s, 1H), 4.08 (t, *J* = 6.0 Hz, 2H), 3.43 (d, *J* = 5.2 Hz, 2H), 3.19 (br s, 2H), 2.17 – 2.00 (m, 2H); ^{13}C NMR (100 MHz, $CDCl_3$) δ 158.9, 153.4, 135.6, 133.7, 129.5, 127.0, 120.8, 114.6, 107.4, 65.9, 40.3, 29.4; IR (thin film): 1620, 1599, 1586, 1510, 1500, 1470, 1291, 1242, 1173, 1151, 824, 755, 692 cm^{-1}; HRMS calculated for $C_{14}H_{18}N_3O$ 244.1444, found 244.1446 $[M+H]^+$.

Supplementary Discussion

Mechanism Study:

Procedure for Standard Reaction Interfered with TEMPO

The standard reaction was performed following the General Procedure with **1a** (38.0 mg, 0.2 mmol), methanol (**2a**) (16.2 μL, 0.4 mmol), TEMPO (32.2 mg, 0.2 mmol), and KO^{t}Bu (44.9 mg, 0.4 mmol) in 0.4 mL DME at 110 ℃ for 12 h. Product **3a** was isolated in 87% yield.

Transition-Metal-Free Cross Coupling of Alkyl Aryl Sulfoxides Besides Methyl Sulfoxides as well as Methyl 2-Naphthyl Sulfide with Methanol.[a]

The reaction was performed following the General Procedure with **1a** (38.0 mg, 0.2 mmol), base (0.4 mmol), additives (0.4 mmol) and methanol (**2a**) (16.2 μL, 0.4 mmol)

in 0.4 mL DME at 110 ℃ for 12 h. Each yield of product **3a** was detected by ^{1}H NMR by using 1.0 equiv. of CH_2Br_2 as an internal standard.

Determination of MeSO$^-$ by HRMS

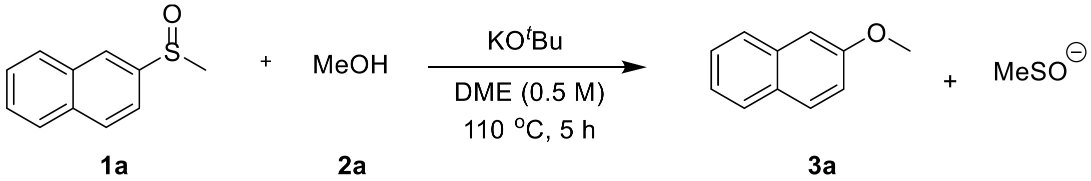

The reaction was performed following the General Procedure with **1a** (38.0 mg, 0.2 mmol), methanol (**2a**) (16.2 μL, 0.4 mmol), and KO^{t}Bu (44.9 mg, 0.4 mmol) in 0.4 mL DME at 110 ℃ for 5 h. The sealed vial was cooled to room temperature, and an aliquot of reaction mixture was taken and immediately analyzed by HRMS in negative ion mode. HRMS calculated for CH_3OS 62.9910, found 62.9912 [M]$^-$.

Details of Computational Studies for Determination of the Mechanism

Possible Mechanisms and Energy Diagram for Mechanism B

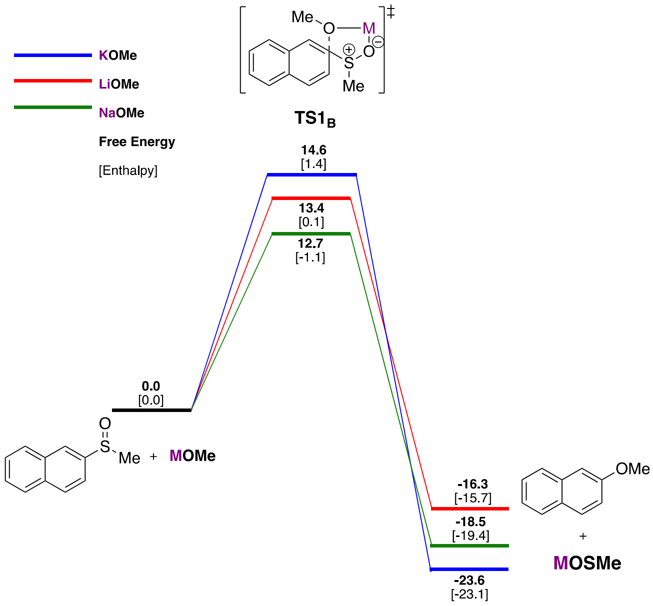

Supplementary Figure 1. Free energy profile for the reaction of **1a** with methoxide in presence of different counterions (M = Li, Na and K). Relative free energy values were calculated with UB3LYP/6-31+G(d).

Details of Computational Studies for Determination of the Mechanism

Optimizations of intermediates and transition states were performed using Gaussian 09[11] with unrestricted-spin DFT using B3LYP functional and basis set 6-31+G(d) all atoms in the gas phase. For all species, vibrational frequencies were also computed at the specified level of theory to obtain thermal Gibbs free energy corrections (at 298 K) and to characterize the stationary points as transition states or minima. Single point energy calculations were performed on the optimized geometries in 1,4-dioxane solvent using SMD solvation model, M06-2X functional, and basis set 6-31+G(d,p)

for all atoms. Calculated single-point energies were converted to the enthalpies and Gibbs free energies using corrections from gas-phase frequency calculations.

NMR Spectra of the products

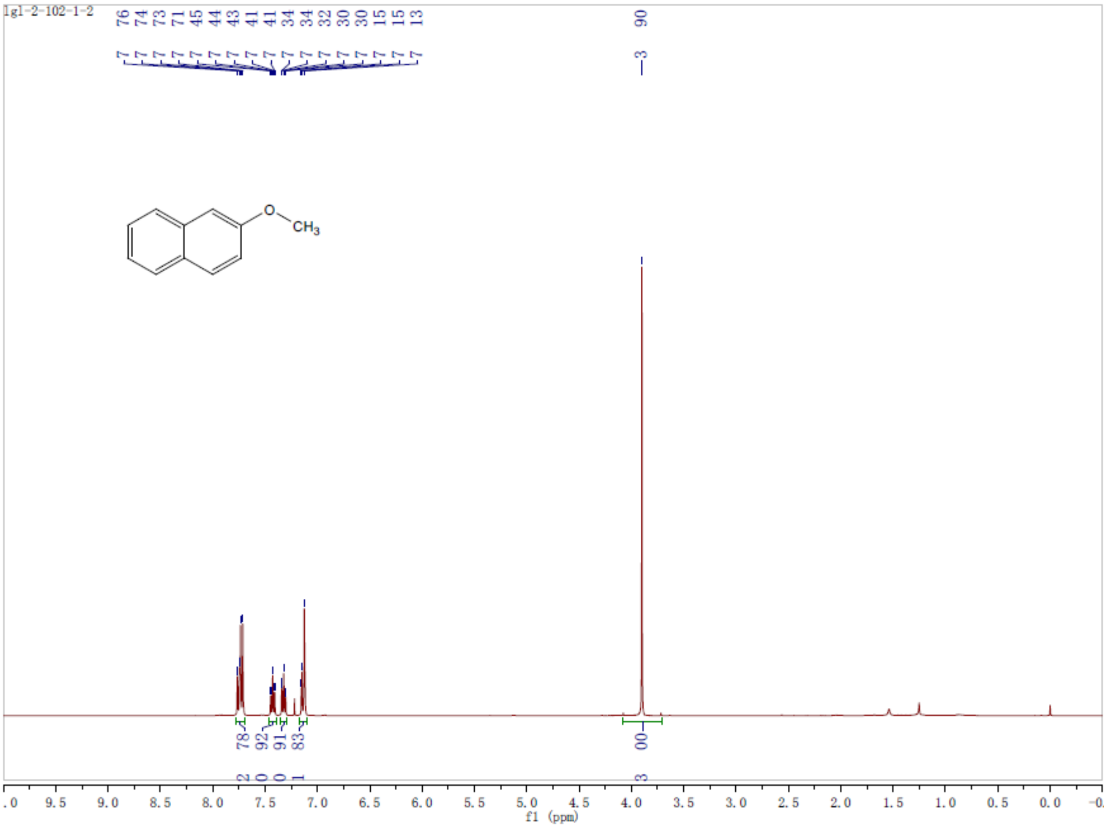

Supplementary Figure 2.^{1}H NMR spectra ($CDCl_3$, 400 MHz) of compound 3a

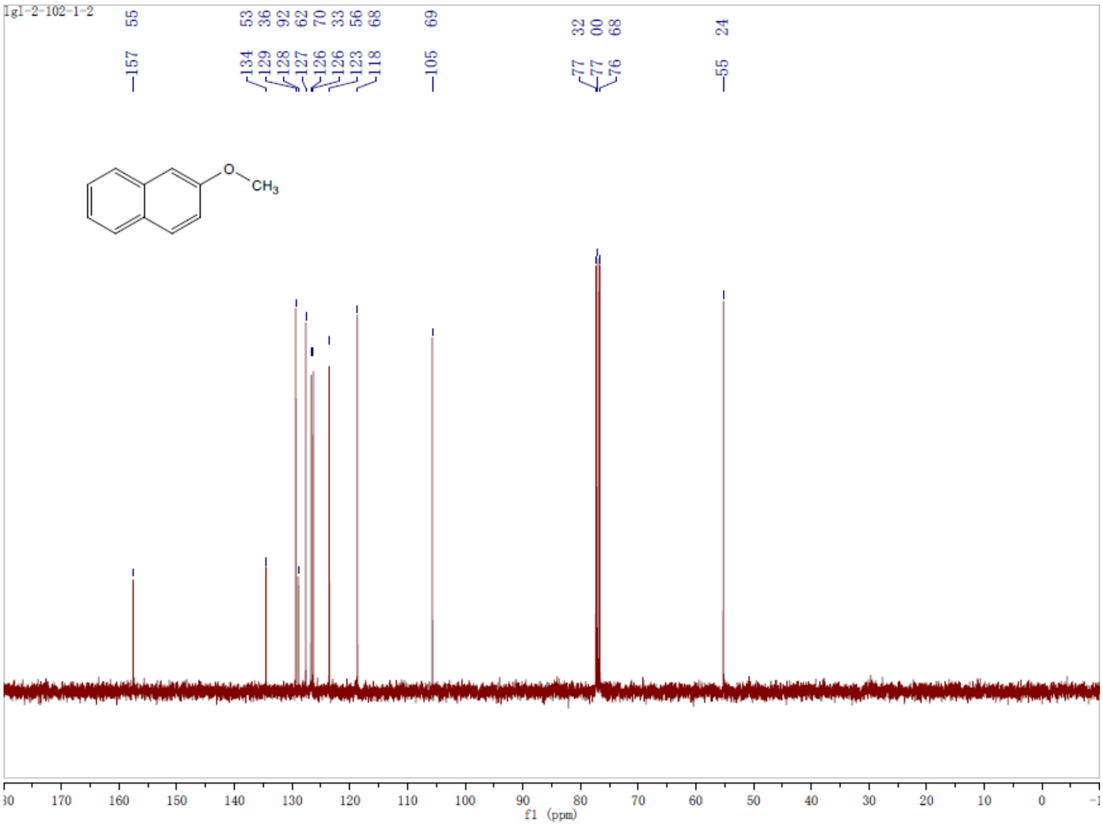

Supplementary Figure 3.^{13}C NMR spectra ($CDCl_3$, 100 MHz) of compound 3a

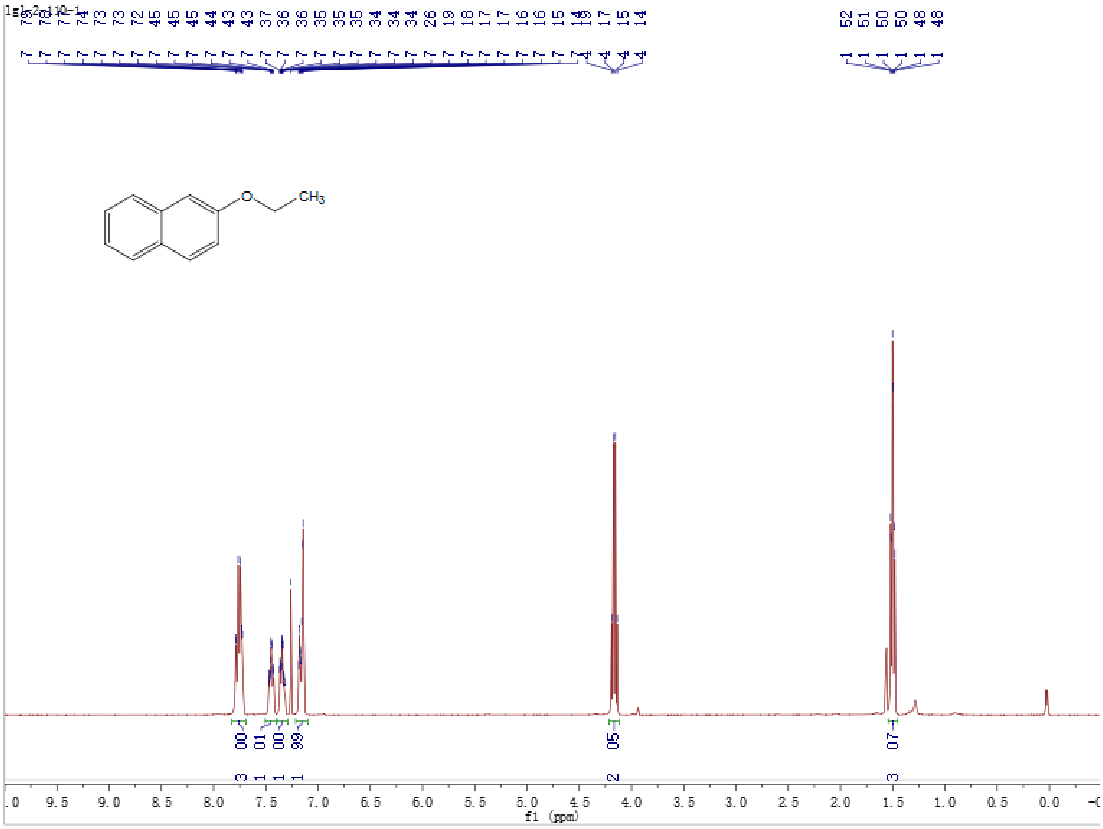

Supplementary Figure 4.^{1}H NMR spectra ($CDCl_3$, 400 MHz) of compound 3b

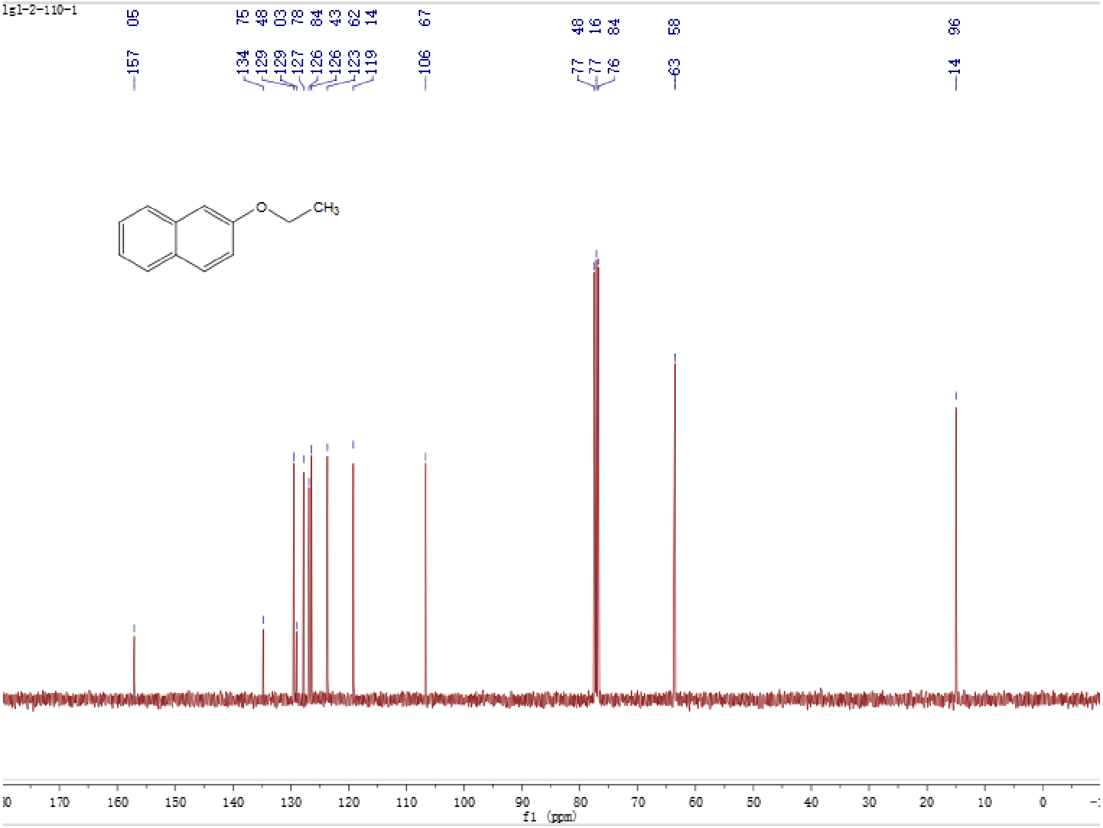

Supplementary Figure 5.^{13}C NMR spectra ($CDCl_3$, 100 MHz) of compound 3b

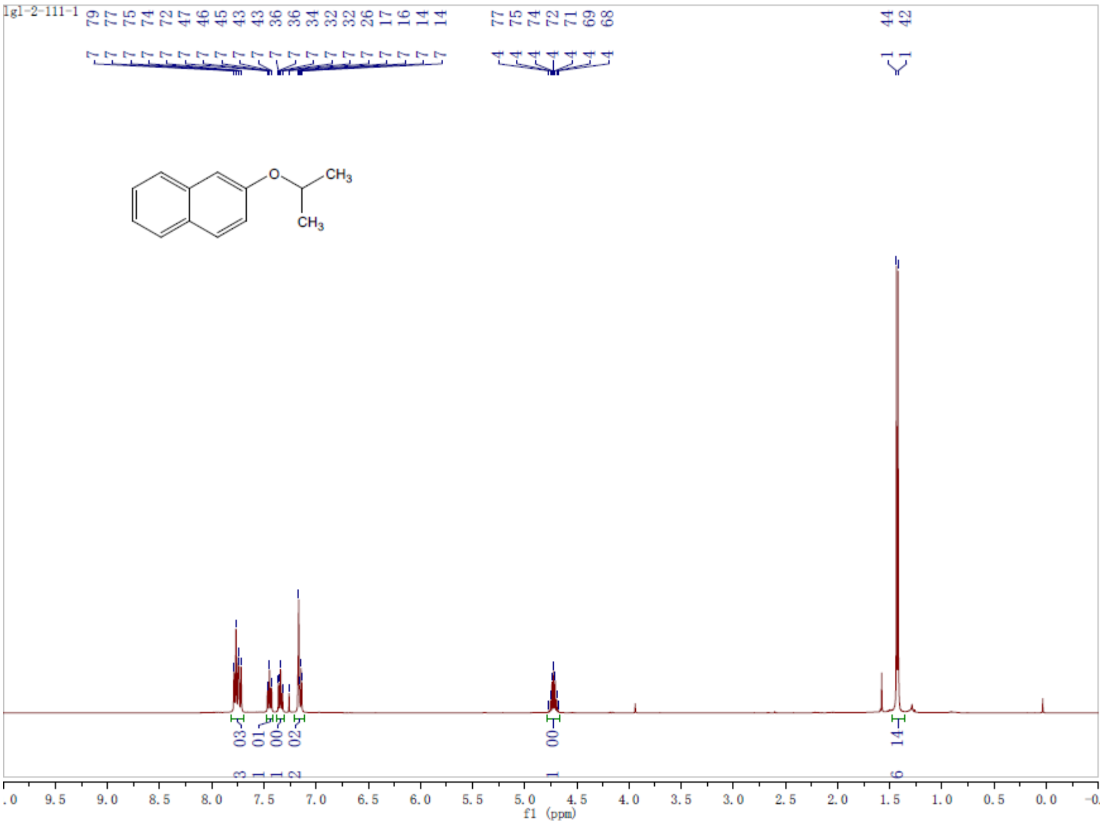

Supplementary Figure 6.^{1}H NMR spectra ($CDCl_3$, 400 MHz) of compound 3c

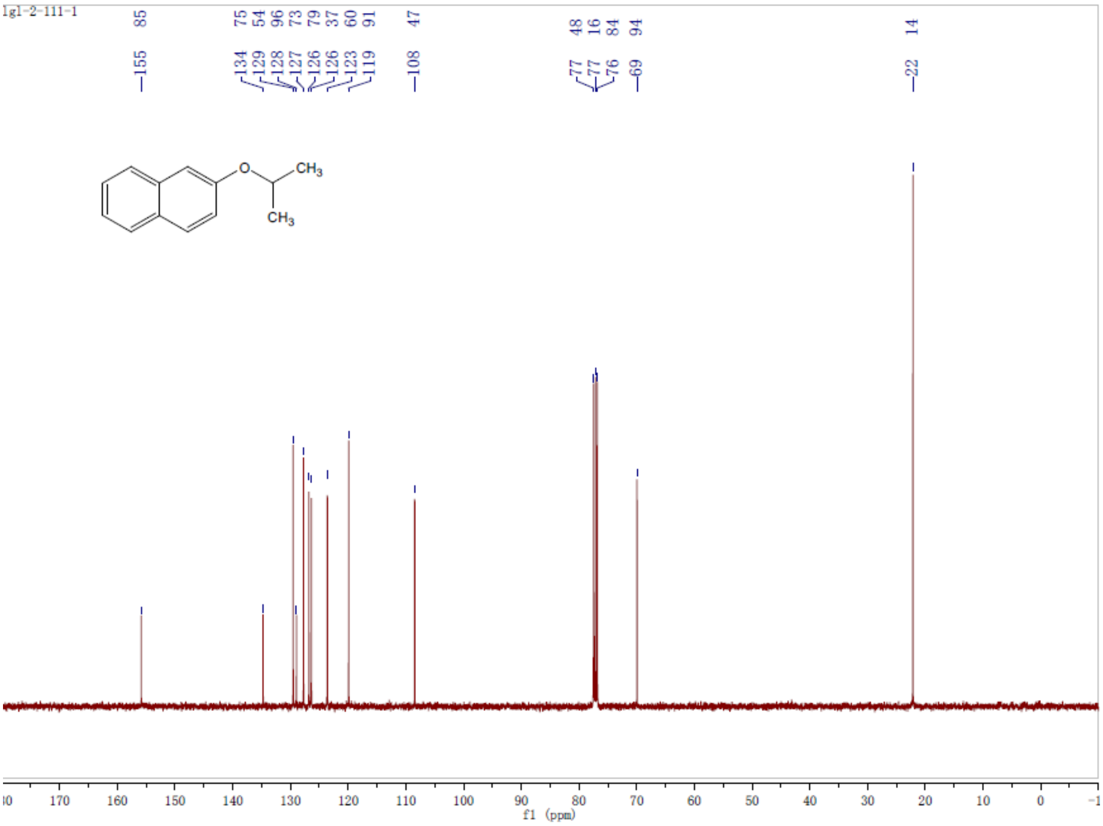

Supplementary Figure 7.^{13}C NMR spectra ($CDCl_3$, 100 MHz) of compound 3c

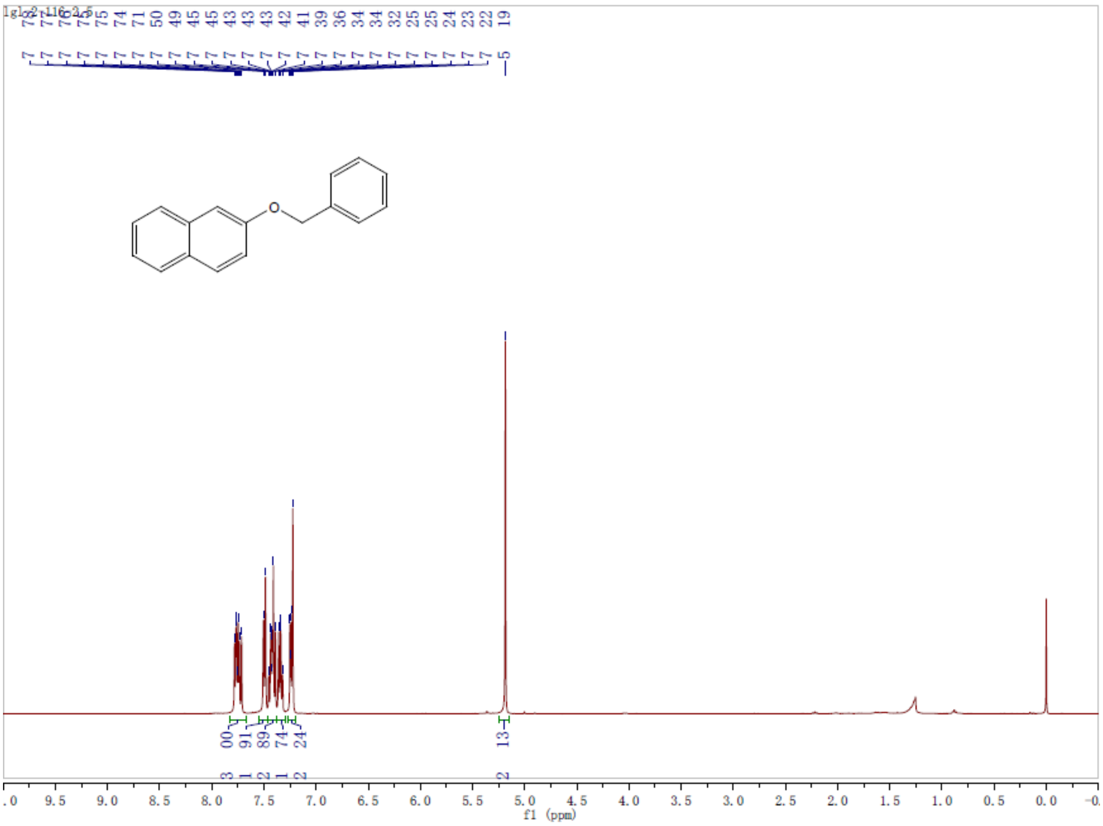

Supplementary Figure 8.^{1}H NMR spectra ($CDCl_3$, 400 MHz) of compound 3d

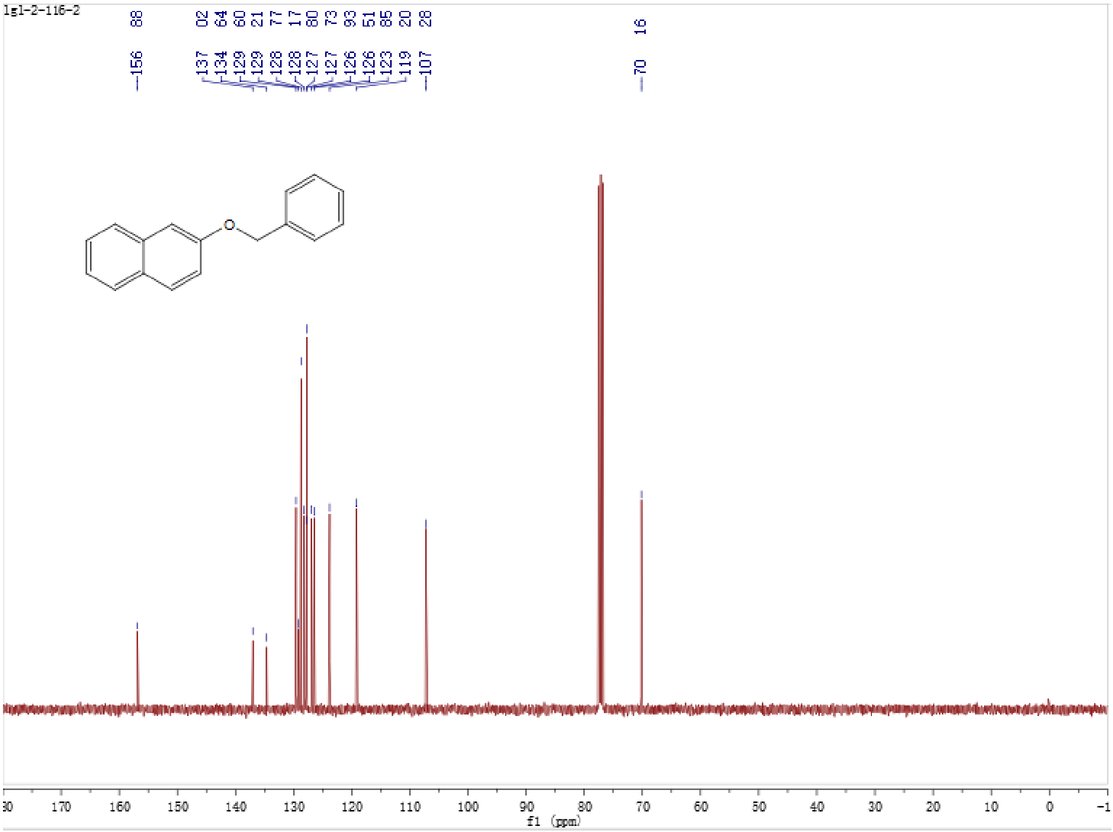

Supplementary Figure 9.^{13}C NMR spectra ($CDCl_3$, 100 MHz) of compound 3d

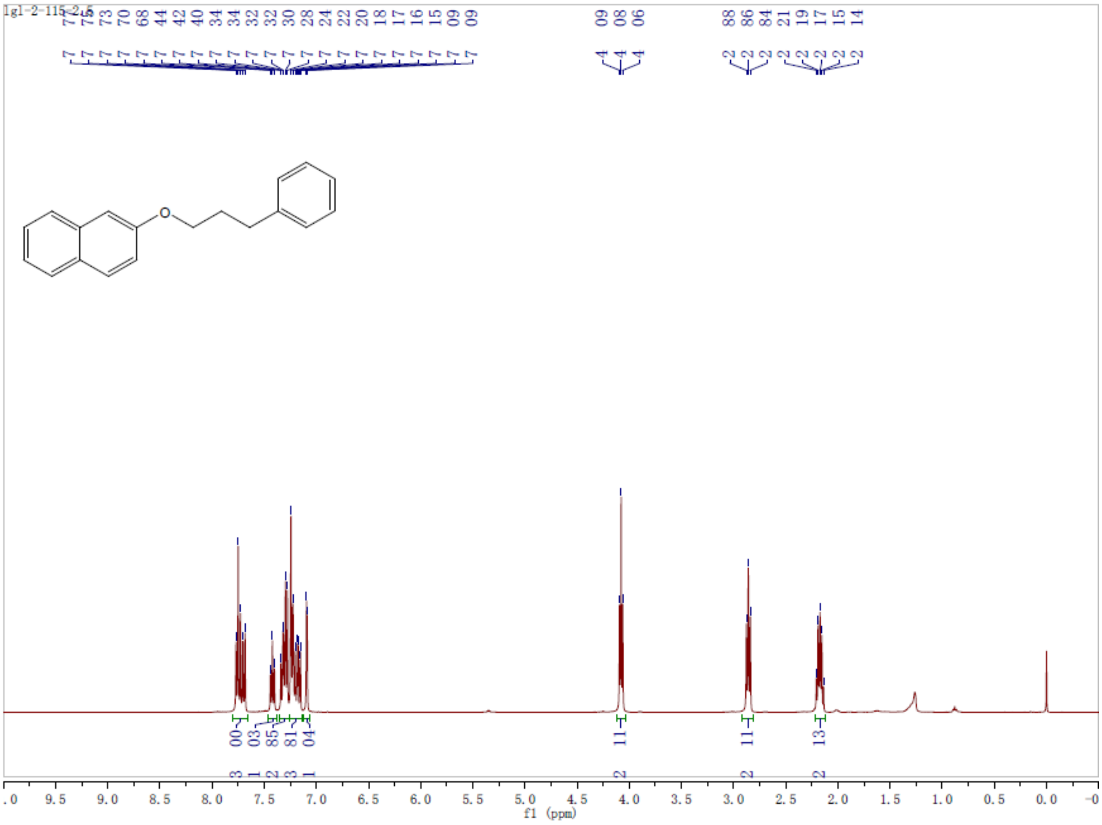

Supplementary Figure 10.^{1}H NMR spectra ($CDCl_3$, 400 MHz) of compound 3e

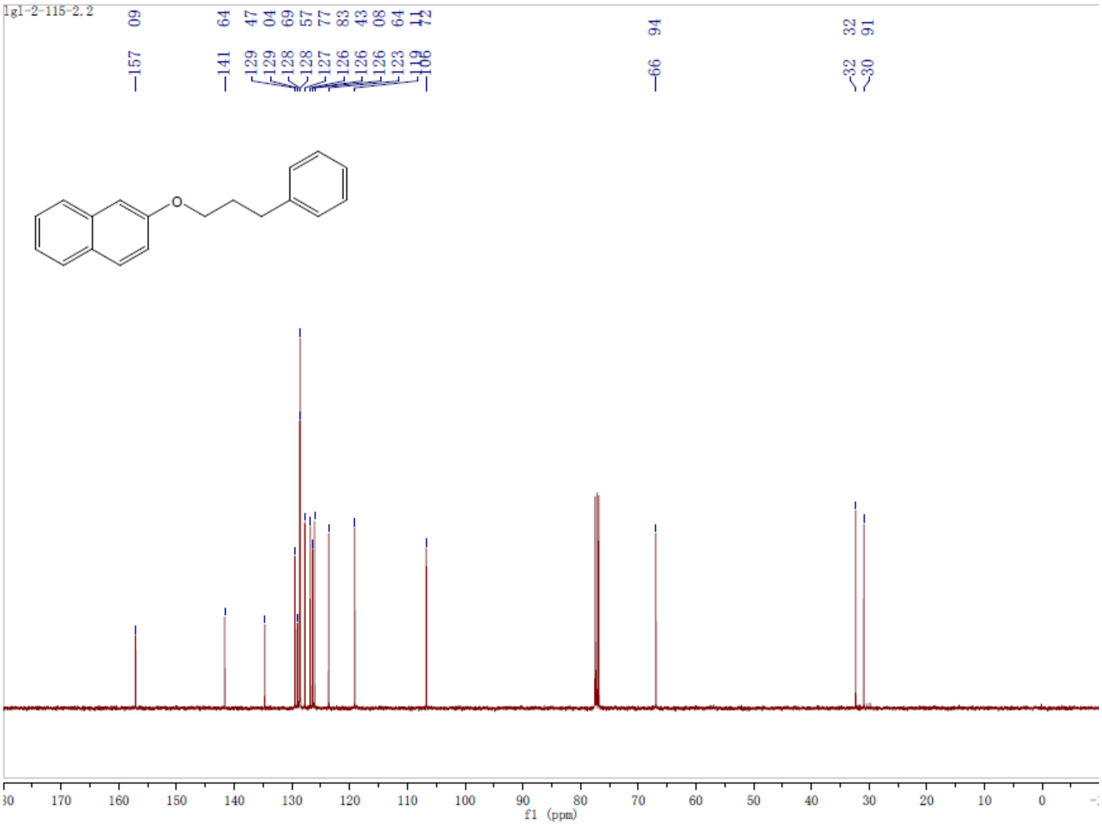

Supplementary Figure 11.^{13}C NMR spectra ($CDCl_3$, 100 MHz) of compound 3e

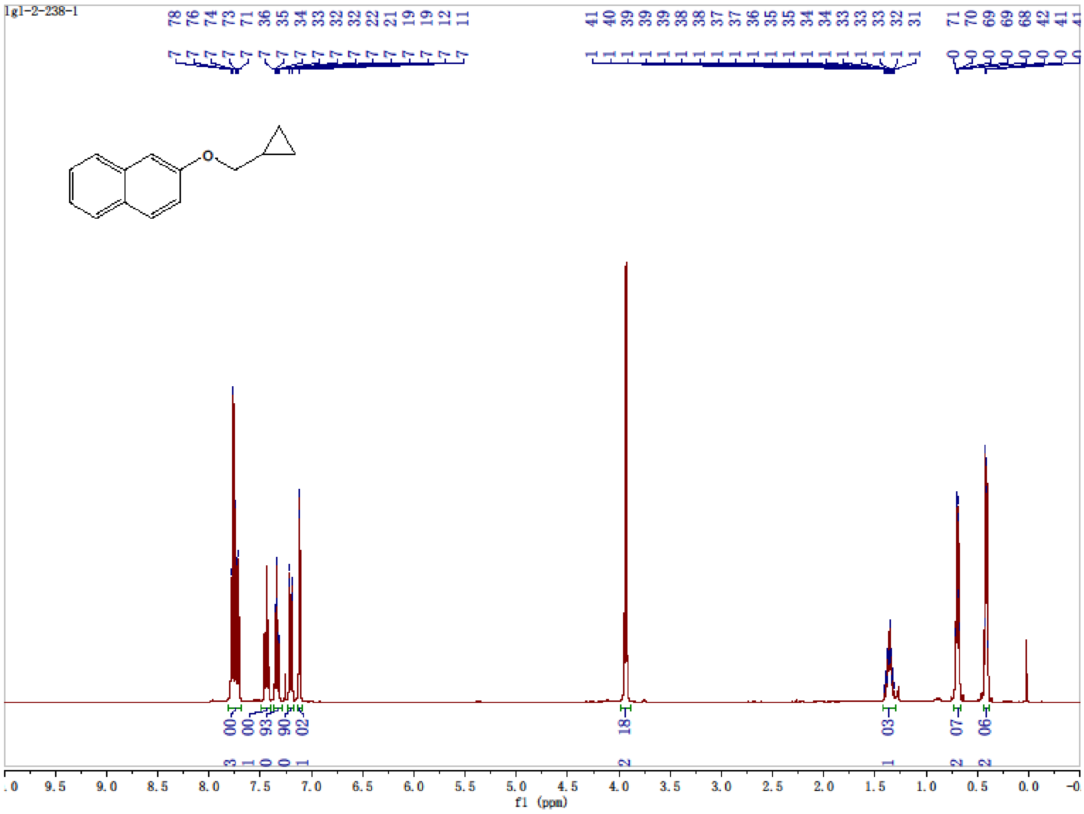

Supplementary Figure 12.^{1}H NMR spectra ($CDCl_3$, 400 MHz) of compound 3f

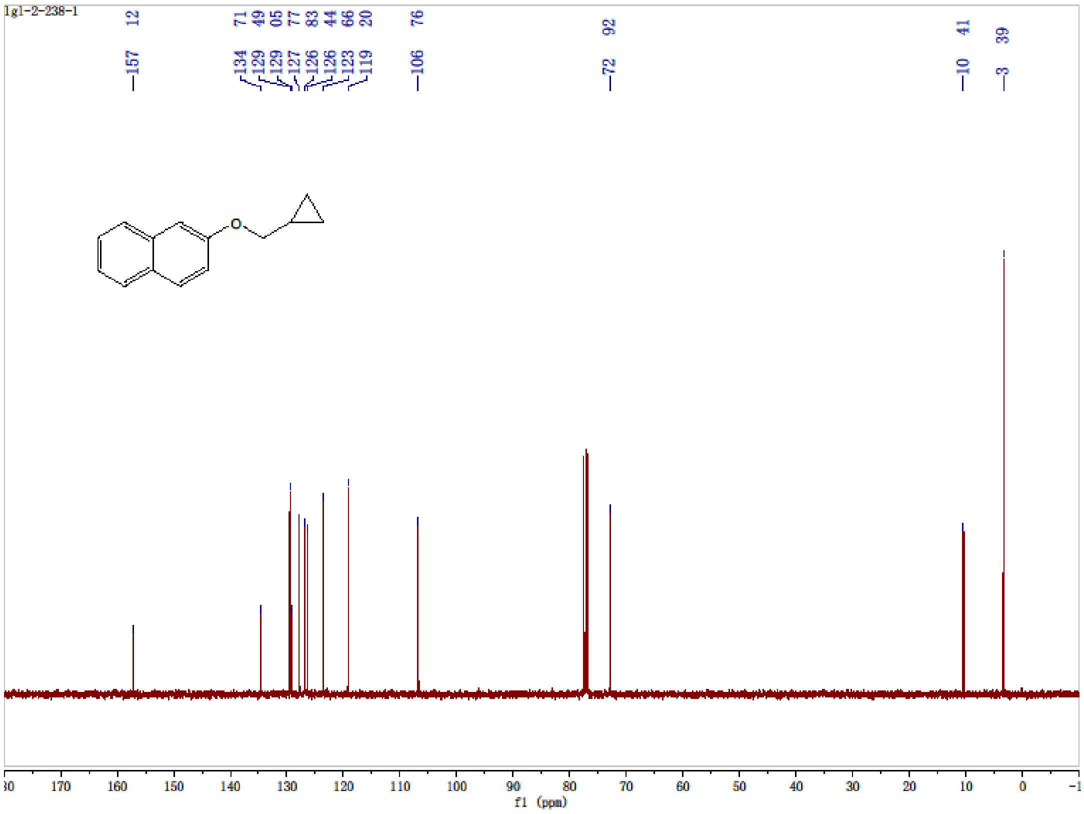

Supplementary Figure 13.^{13}C NMR spectra ($CDCl_3$, 100 MHz) of compound 3f

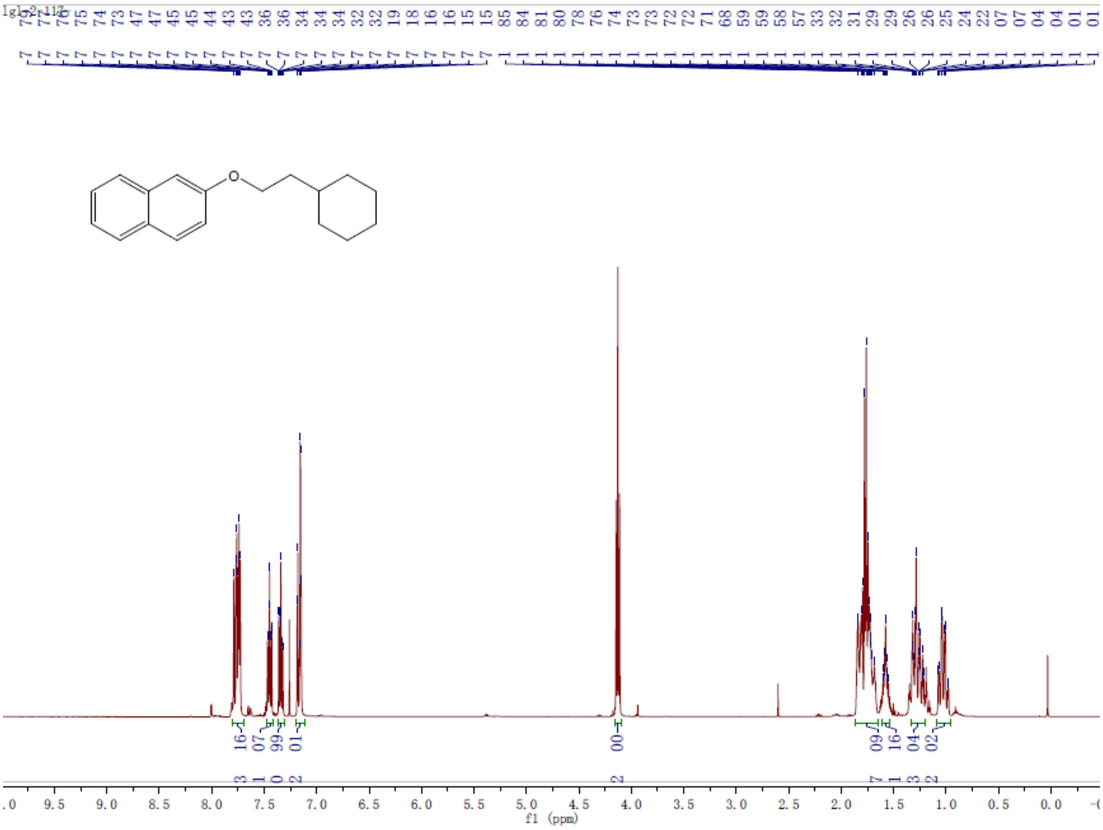

Supplementary Figure 14. ^{1}**H NMR spectra (CDCl$_3$, 400 MHz) of compound 3g**

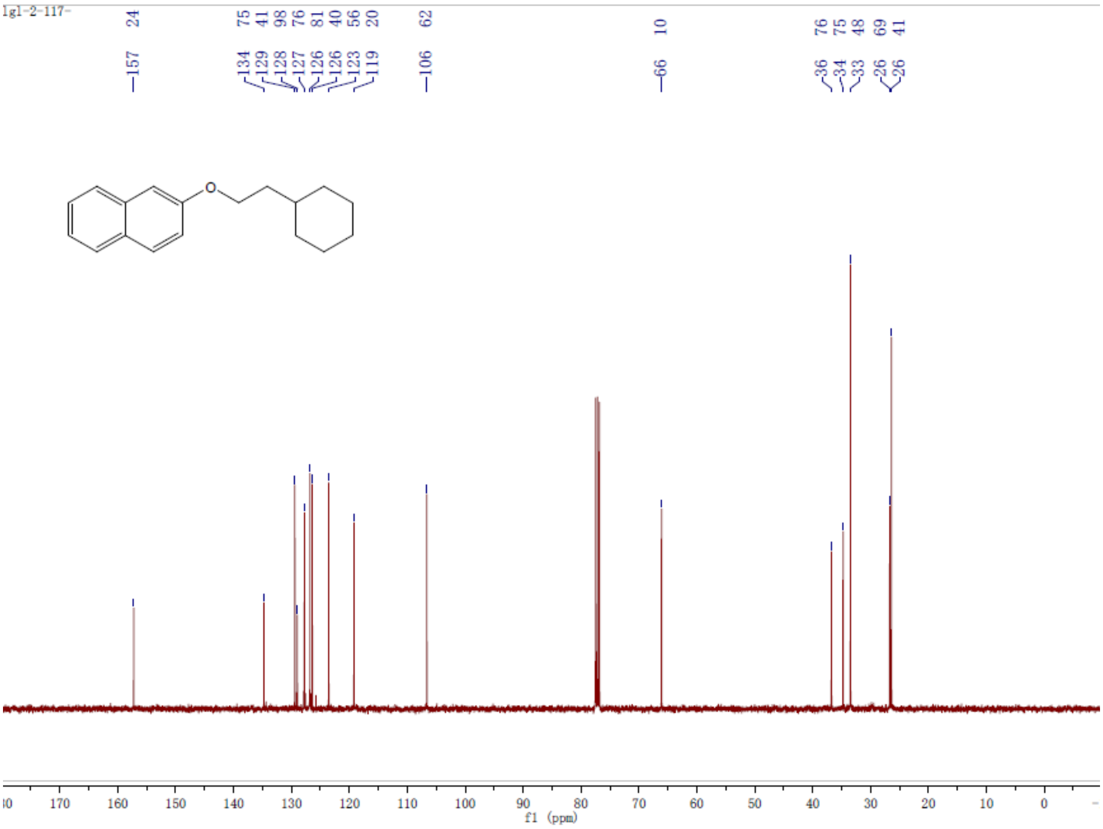

Supplementary Figure 15. ^{13}**C NMR spectra (CDCl$_3$, 100 MHz) of compound 3g**

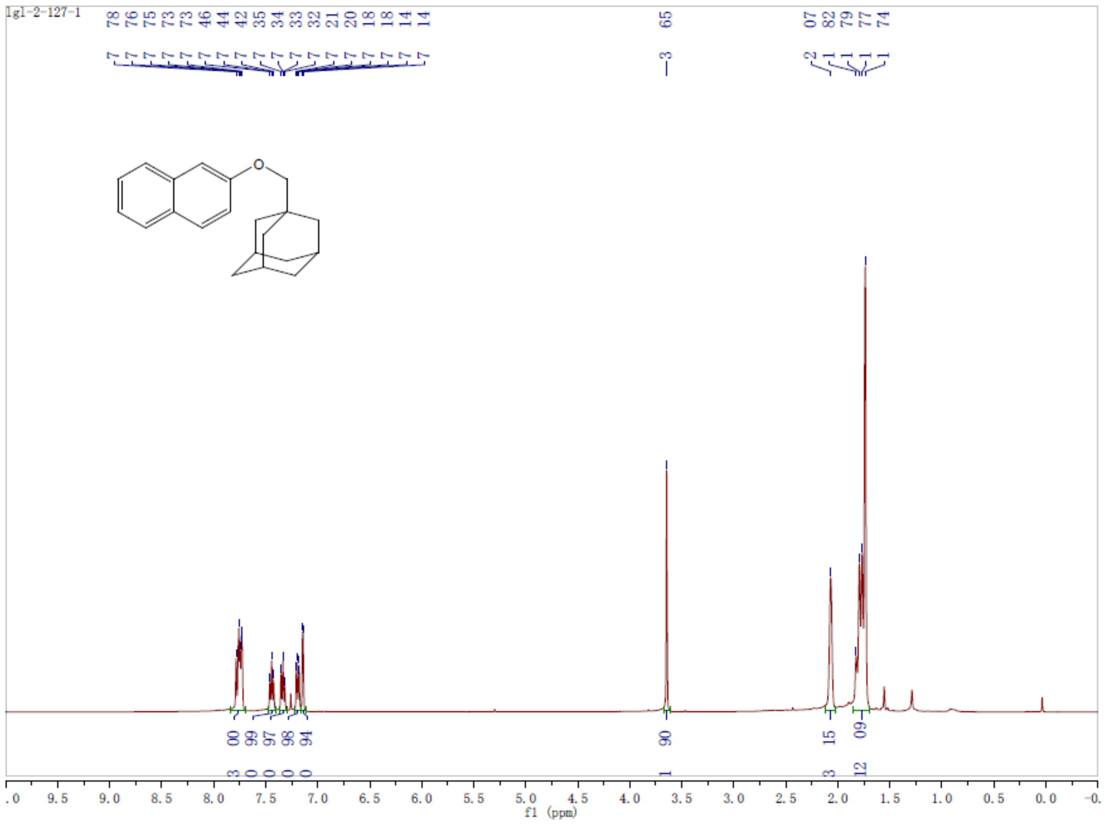

Supplementary Figure 16.^{1}H NMR spectra ($CDCl_3$, 400 MHz) of compound 3h

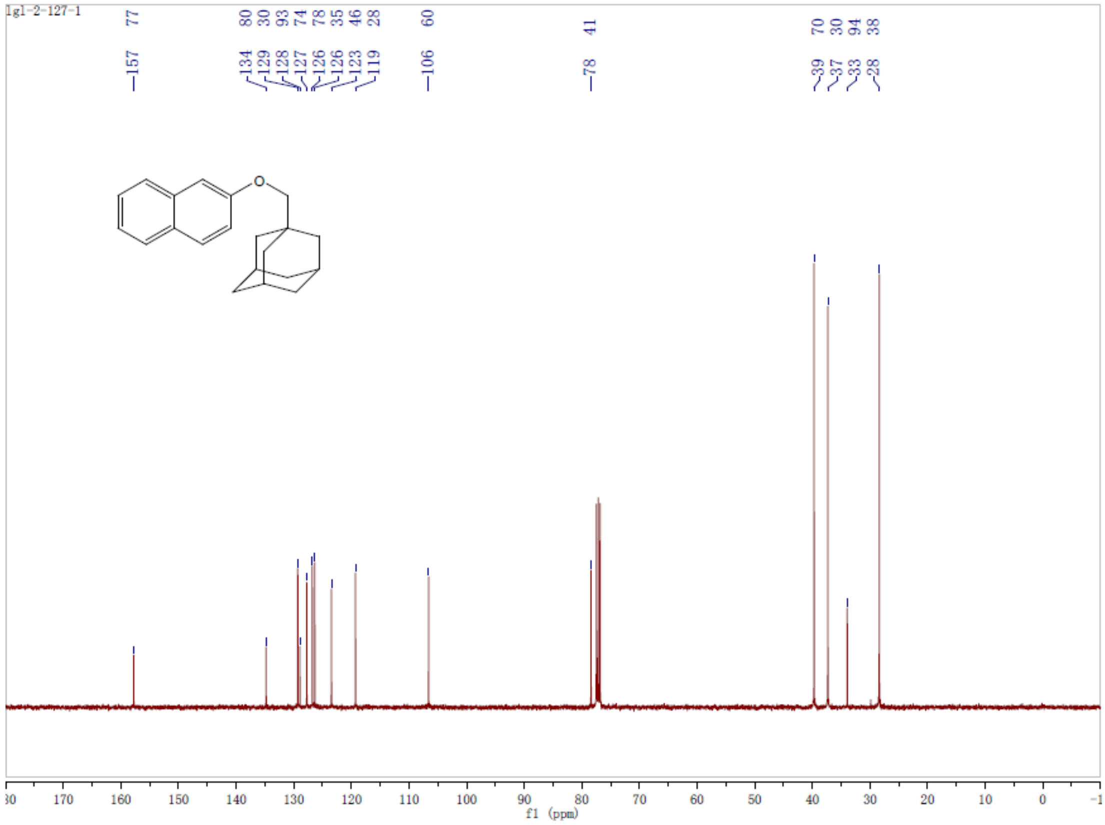

Supplementary Figure 17.^{13}C NMR spectra ($CDCl_3$, 100 MHz) of compound 3h

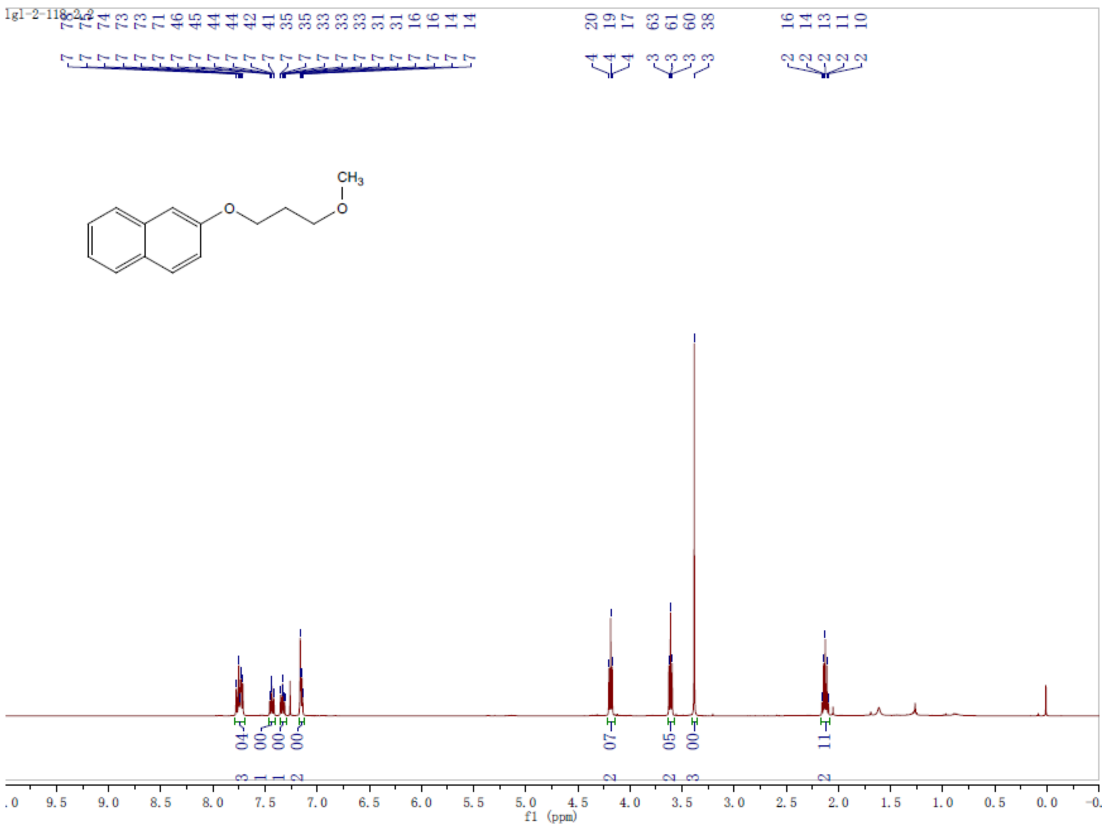

Supplementary Figure 18. ^{1}H **NMR spectra ($CDCl_3$, 400 MHz) of compound 3i**

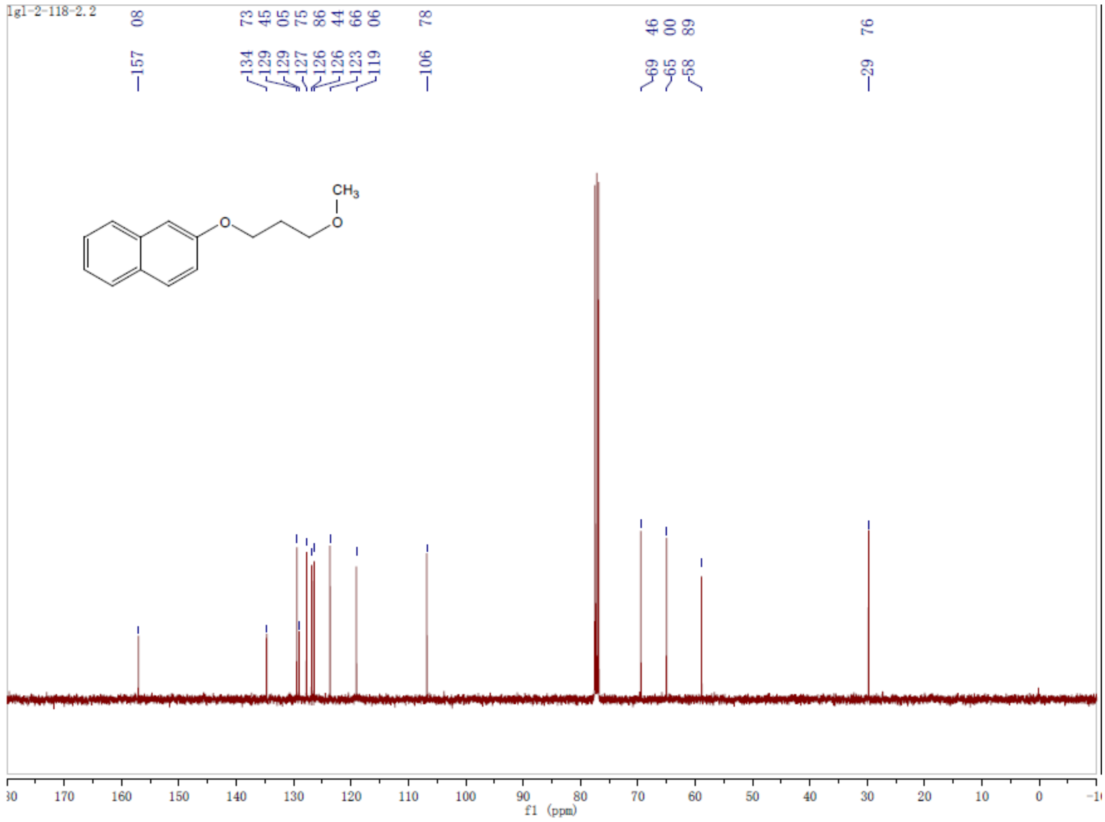

Supplementary Figure 19. ^{13}C **NMR spectra ($CDCl_3$, 100 MHz) of compound 3i**

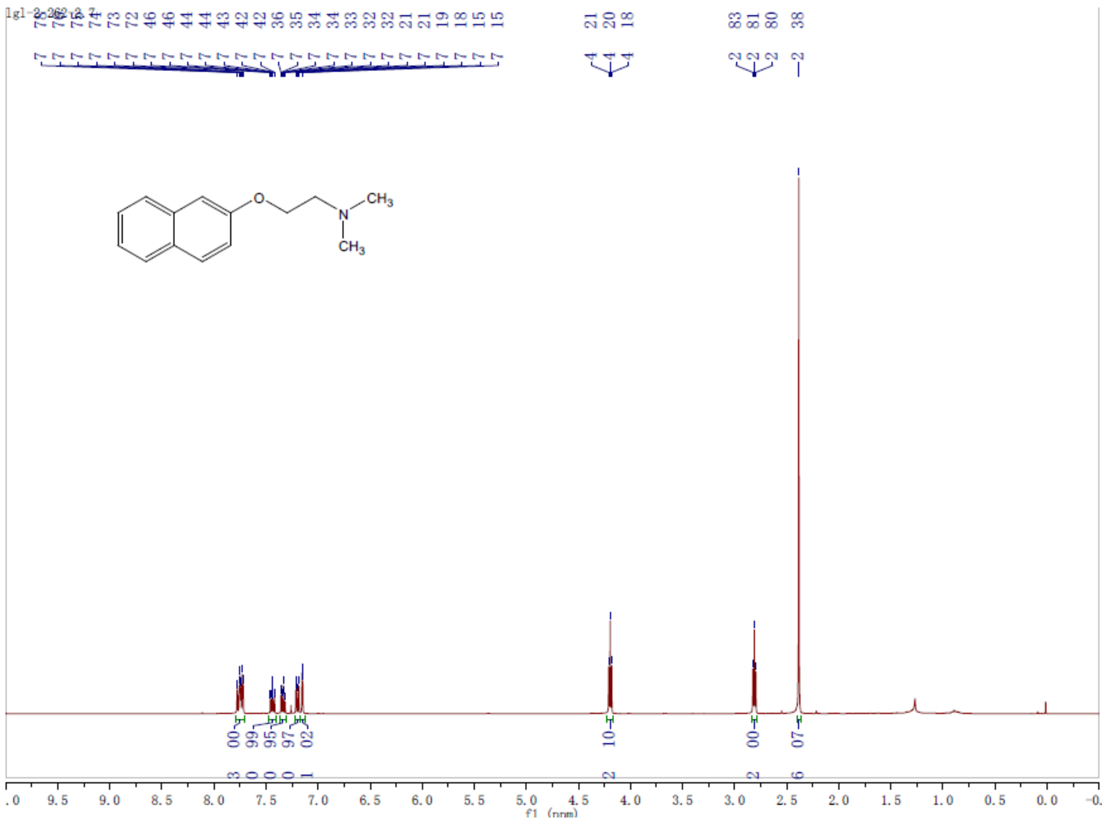

Supplementary Figure 20. ^{1}H NMR spectra ($CDCl_3$, 400 MHz) of compound 3j

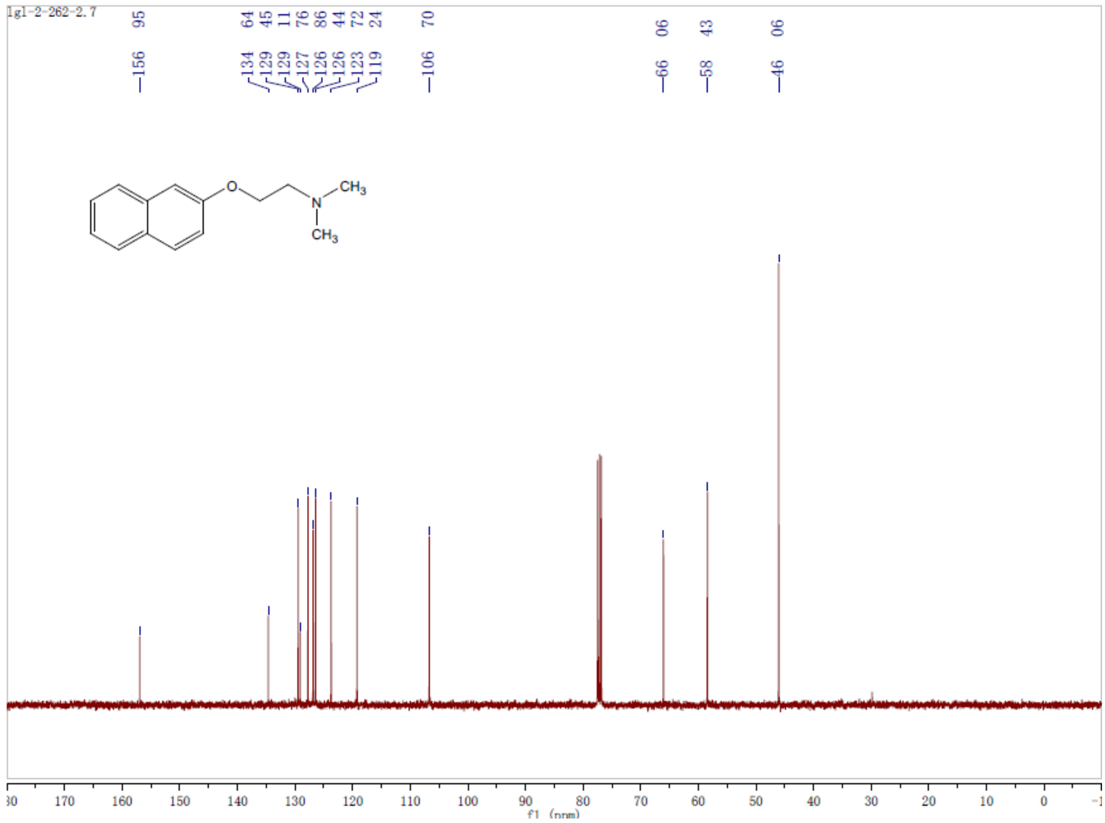

Supplementary Figure 21. ^{13}C NMR spectra ($CDCl_3$, 100 MHz) of compound 3j

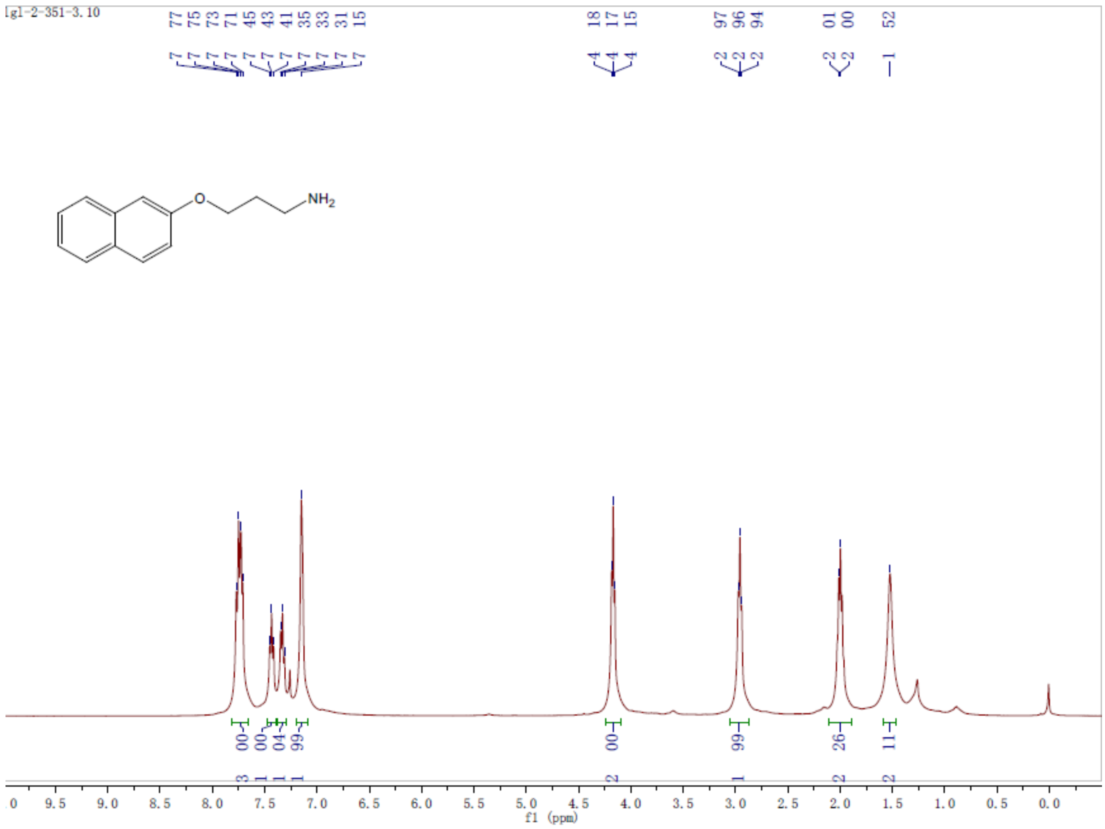

Supplementary Figure 22.^{1}H NMR spectra ($CDCl_3$, 400 MHz) of compound 3k

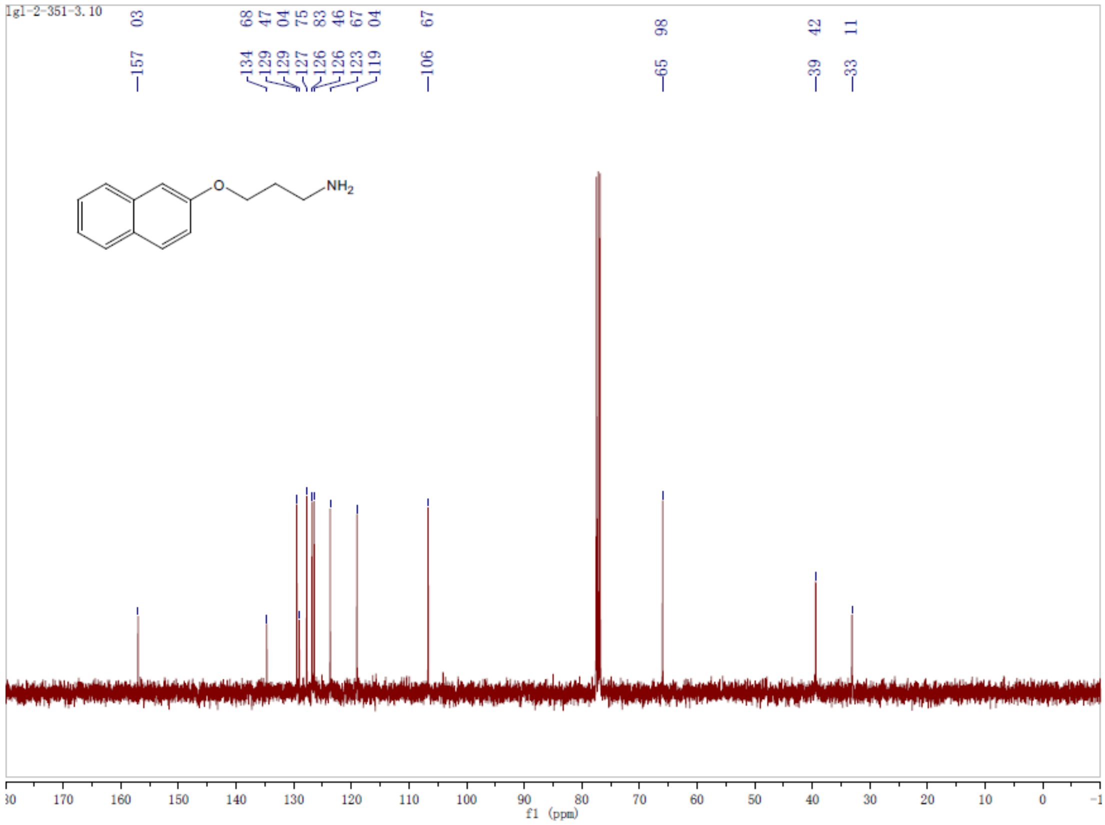

Supplementary Figure 23.^{13}C NMR spectra ($CDCl_3$, 100 MHz) of compound 3k

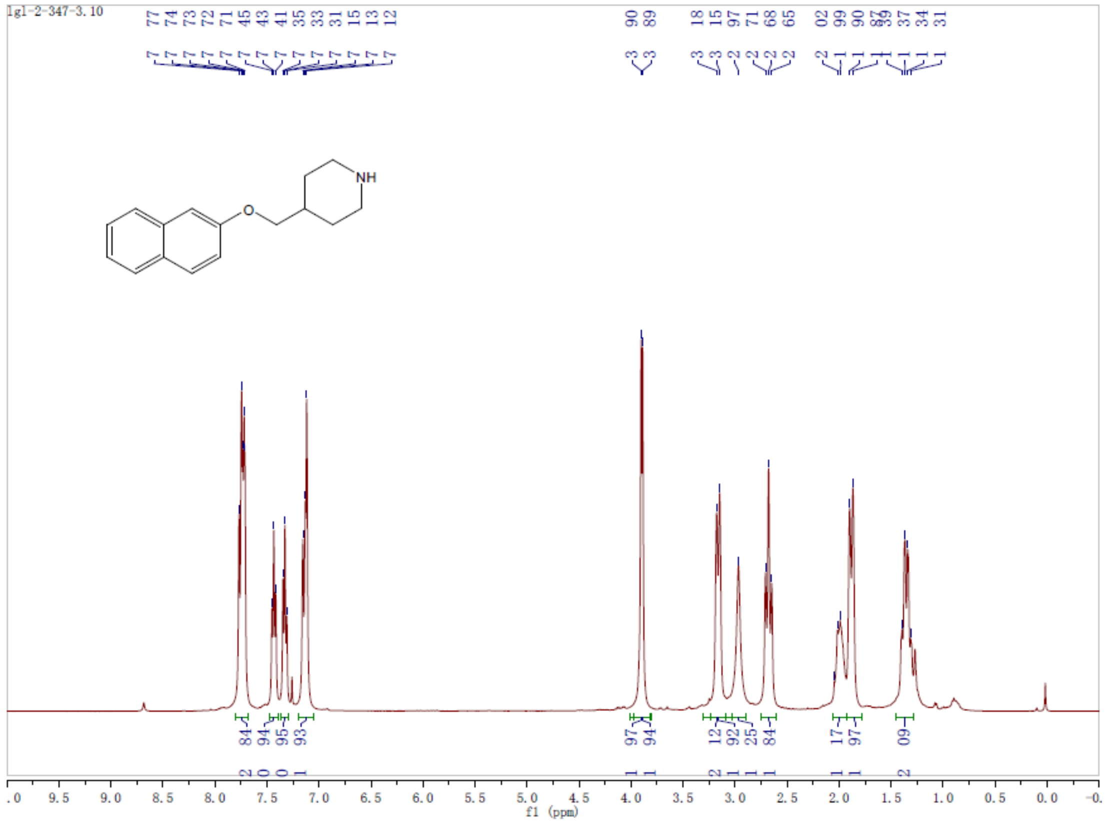

Supplementary Figure 24.^{1}H NMR spectra ($CDCl_3$, 400 MHz) of compound 3l

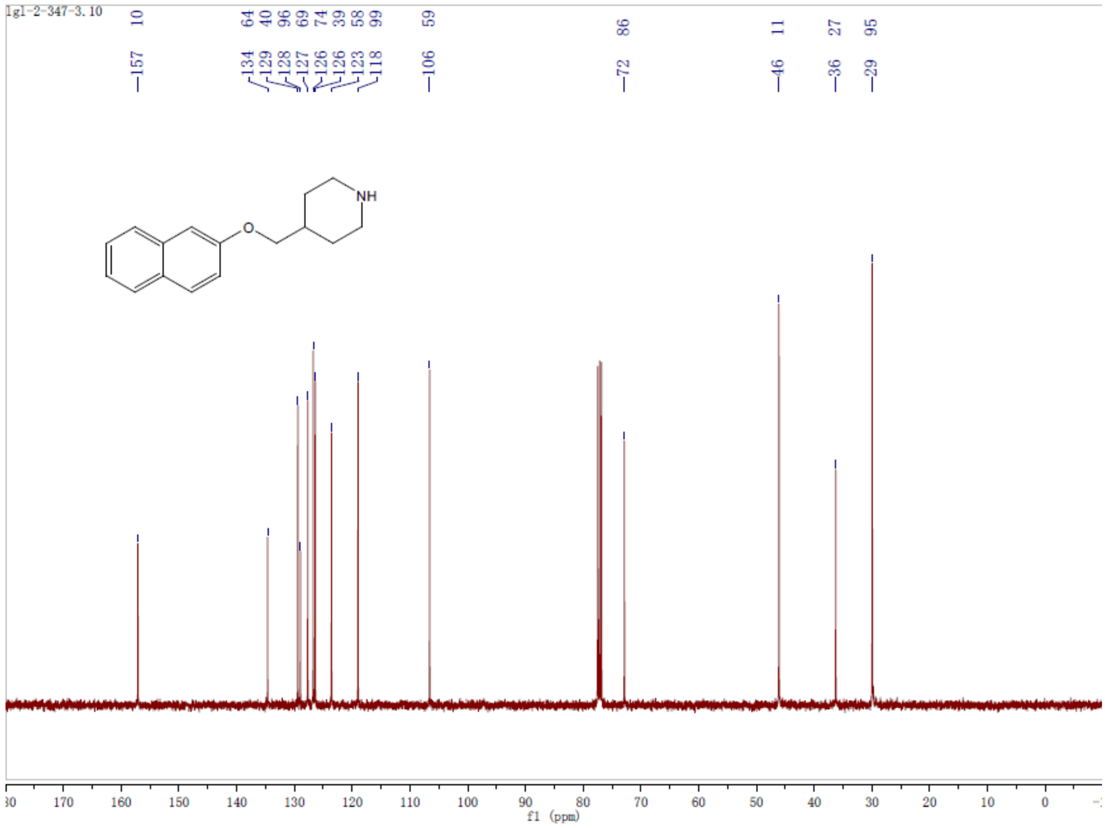

Supplementary Figure 25.^{13}C NMR spectra ($CDCl_3$, 100 MHz) of compound 3l

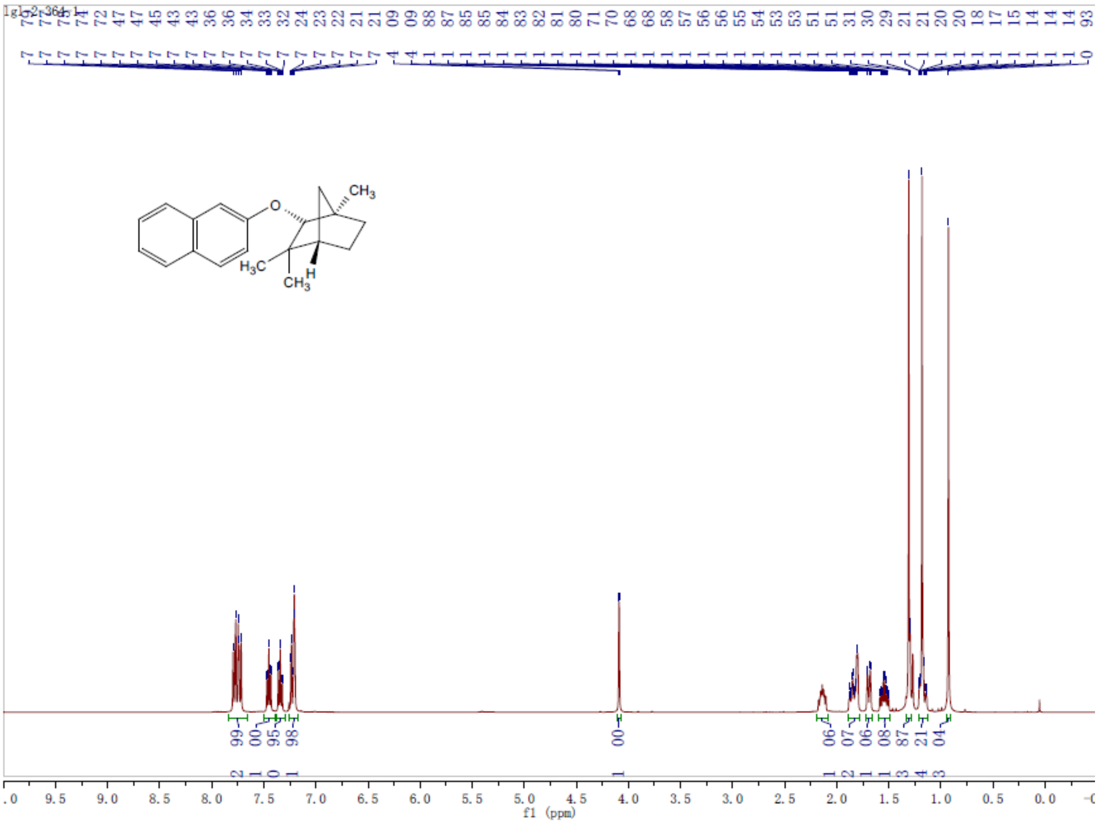

Supplementary Figure 26.^{1}H NMR spectra ($CDCl_3$, 400 MHz) of compound 3m

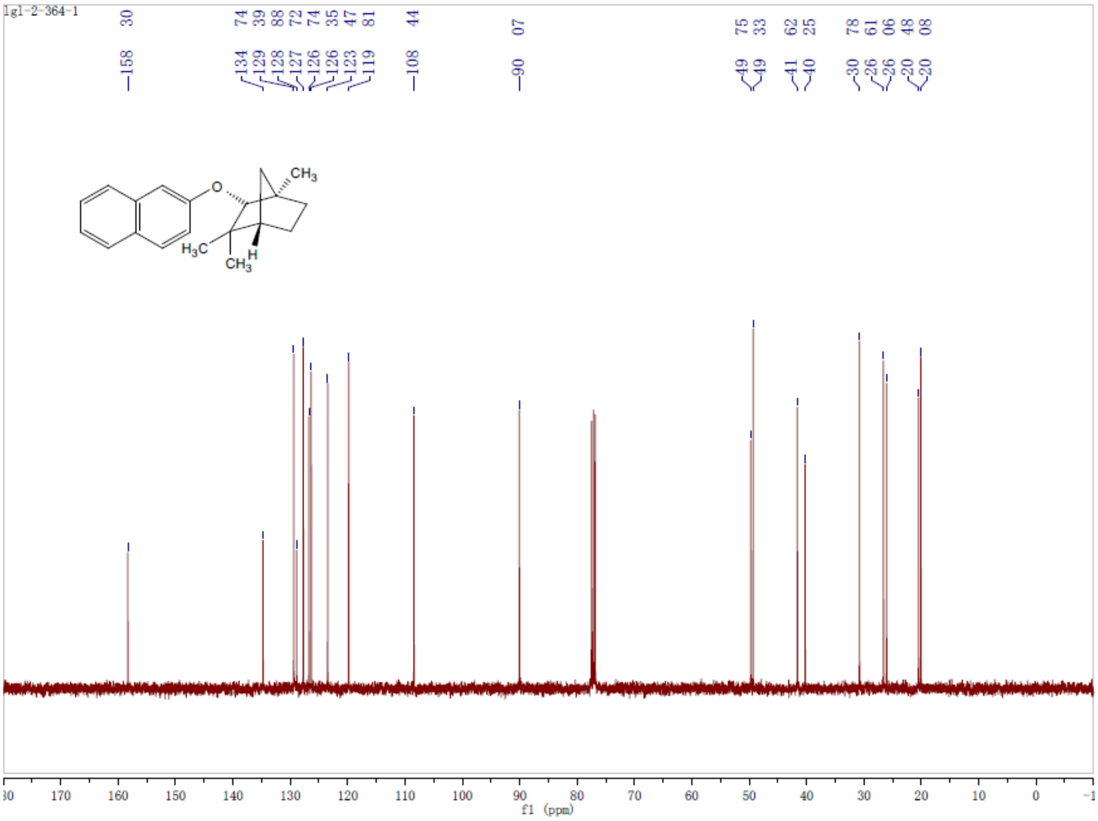

Supplementary Figure 27.^{13}C NMR spectra ($CDCl_3$, 100 MHz) of compound 3m

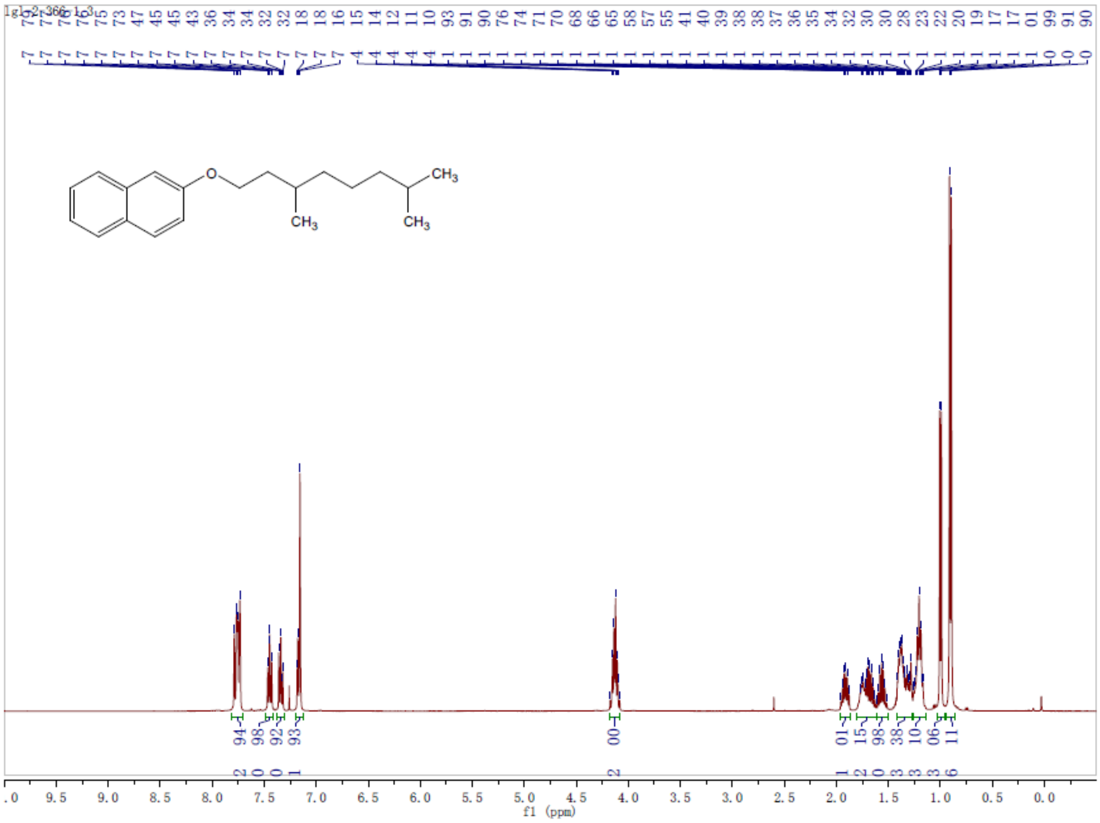

Supplementary Figure 28. ^{1}H **NMR spectra (** $CDCl_3$ **, 400 MHz) of compound 3n**

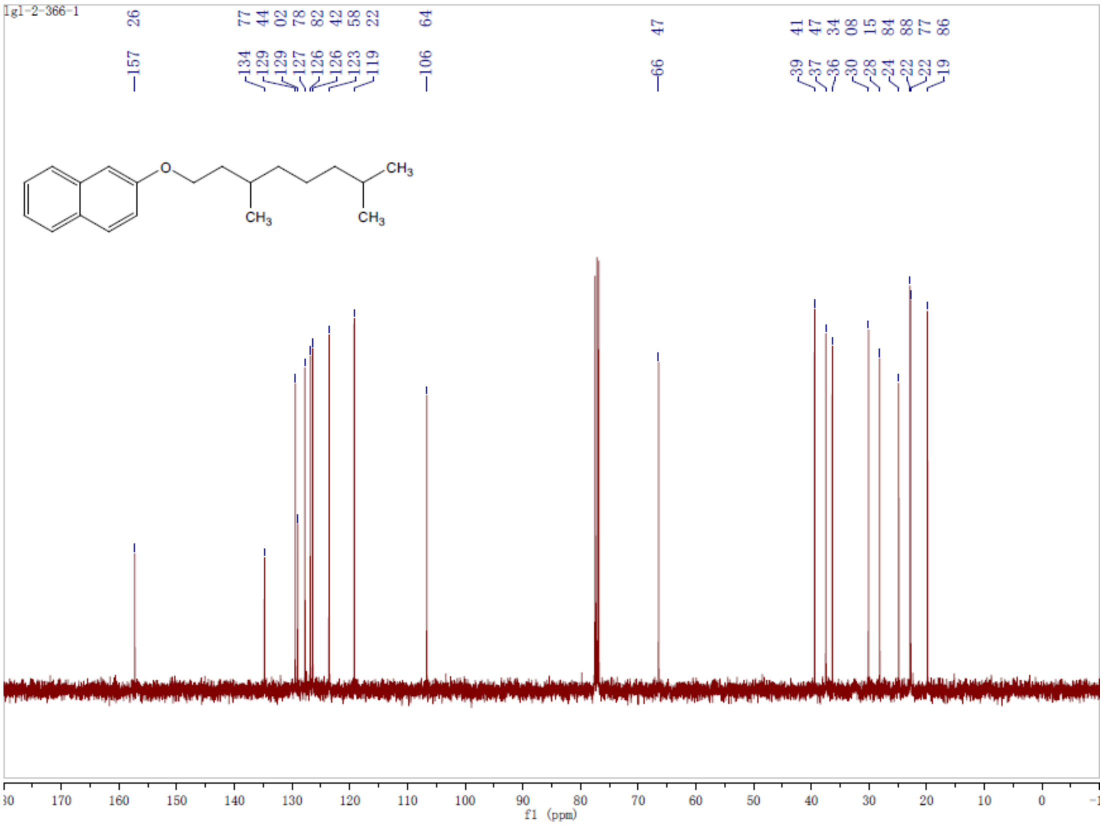

Supplementary Figure 29. ^{13}C **NMR spectra (** $CDCl_3$ **, 100 MHz) of compound 3n**

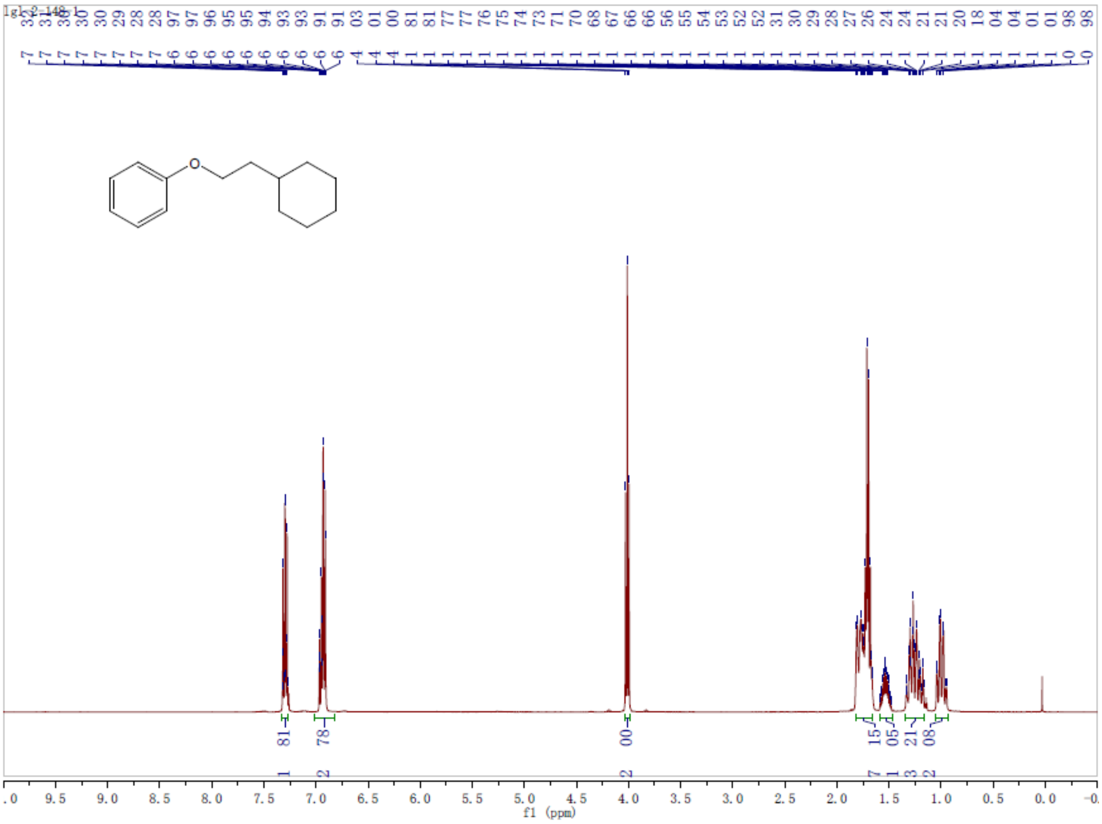

Supplementary Figure 30.^{1}H NMR spectra ($CDCl_3$, 400 MHz) of compound 4a

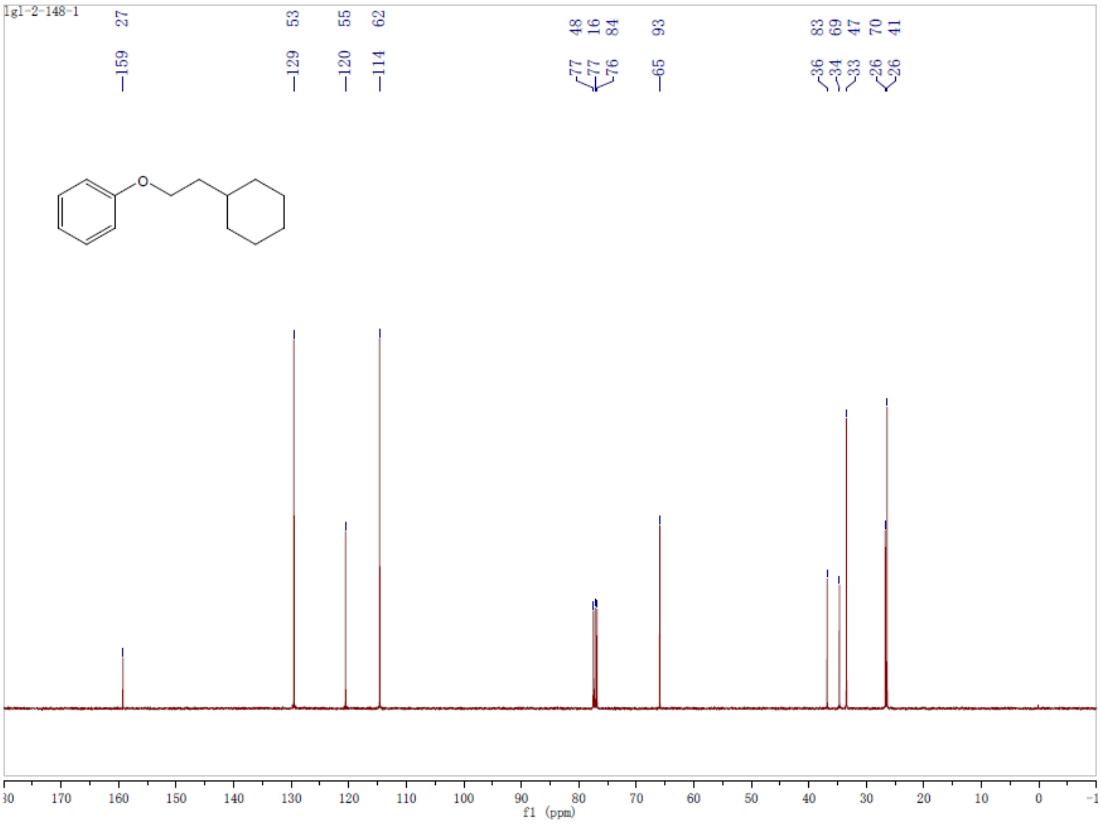

Supplementary Figure 31.^{13}C NMR spectra ($CDCl_3$, 100 MHz) of compound 4a

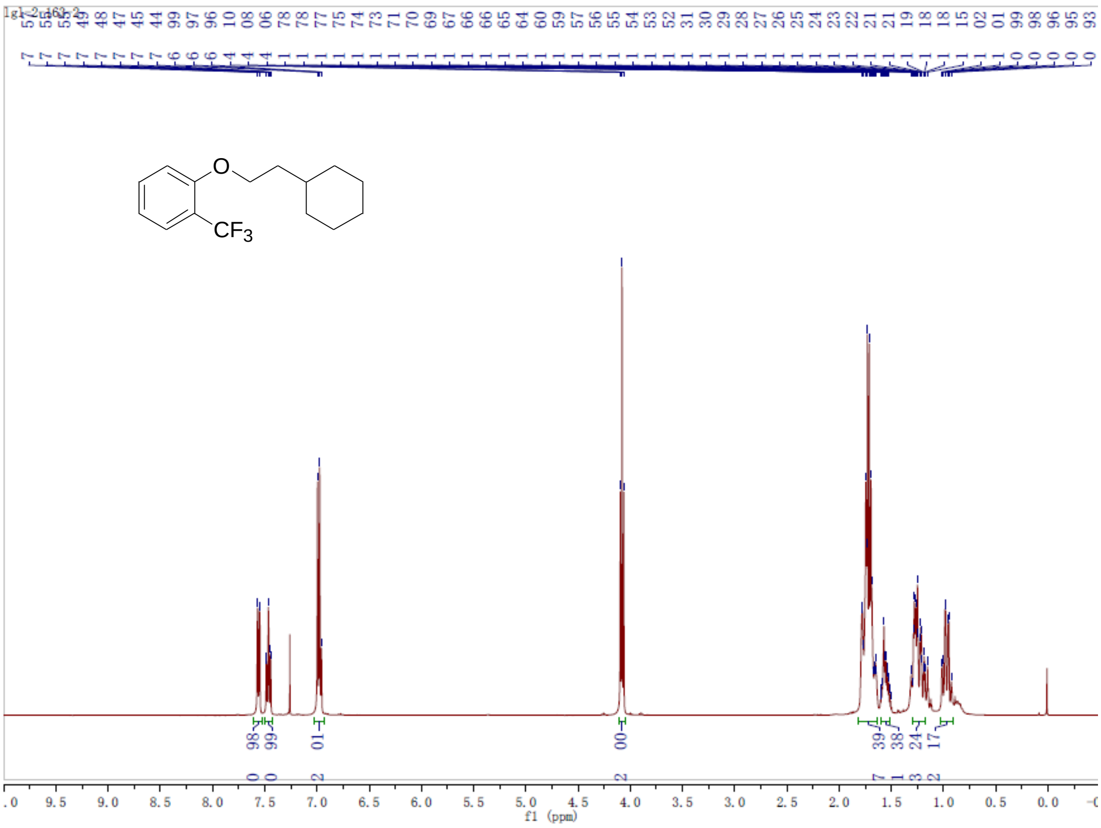

Supplementary Figure 32.^{1}H NMR spectra ($CDCl_3$, 400 MHz) of compound 4b

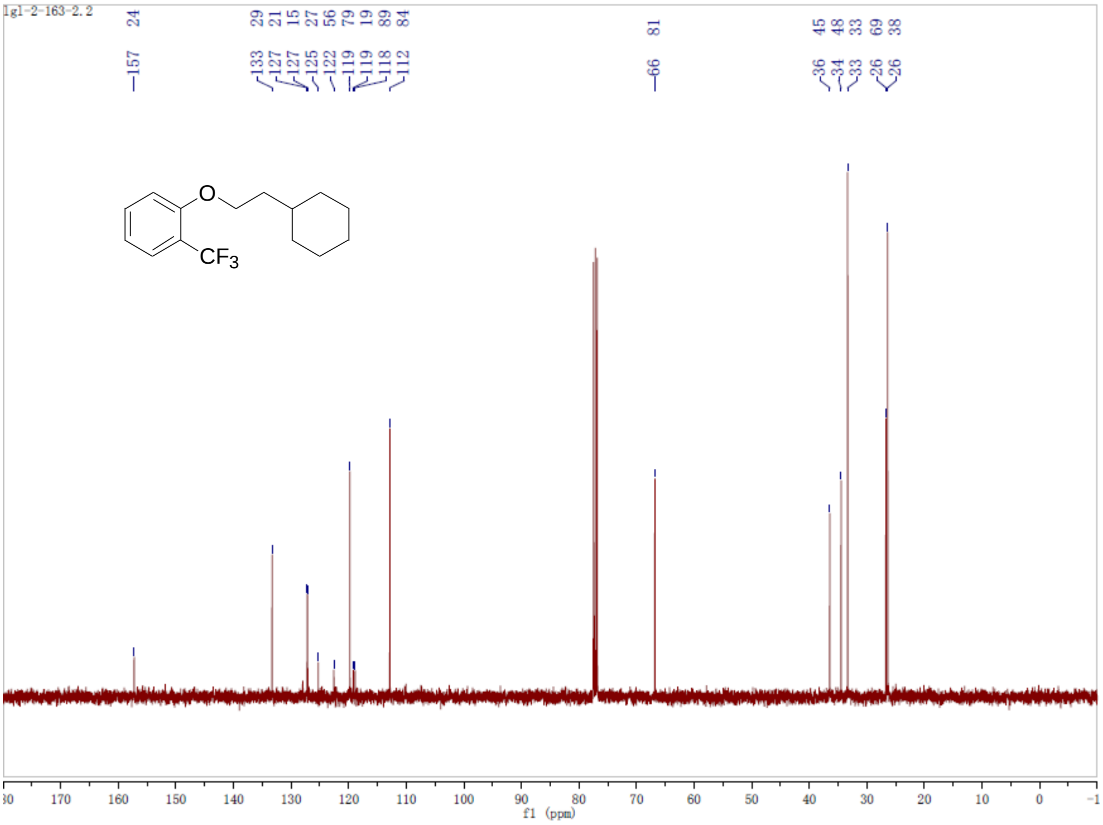

Supplementary Figure 33.^{13}C NMR spectra ($CDCl_3$, 100 MHz) of compound 4b

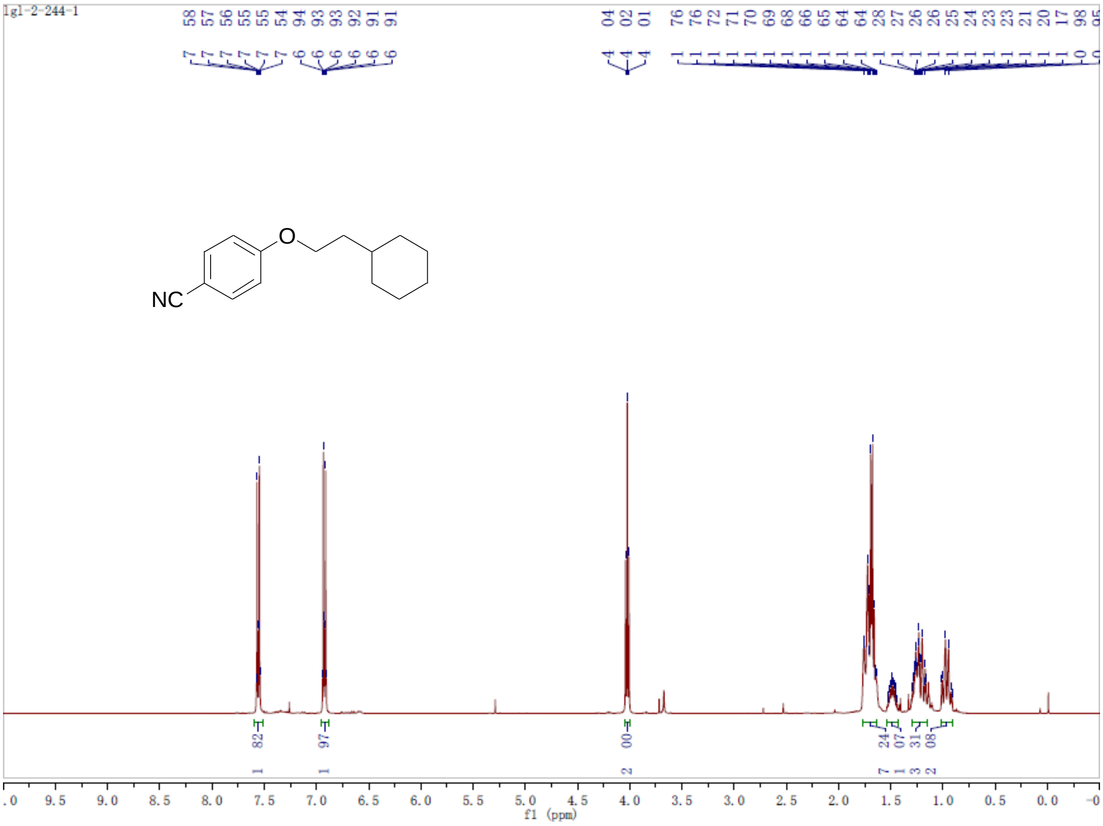

Supplementary Figure 34. ^{1}H **NMR spectra ($CDCl_3$, 400 MHz) of compound 4c**

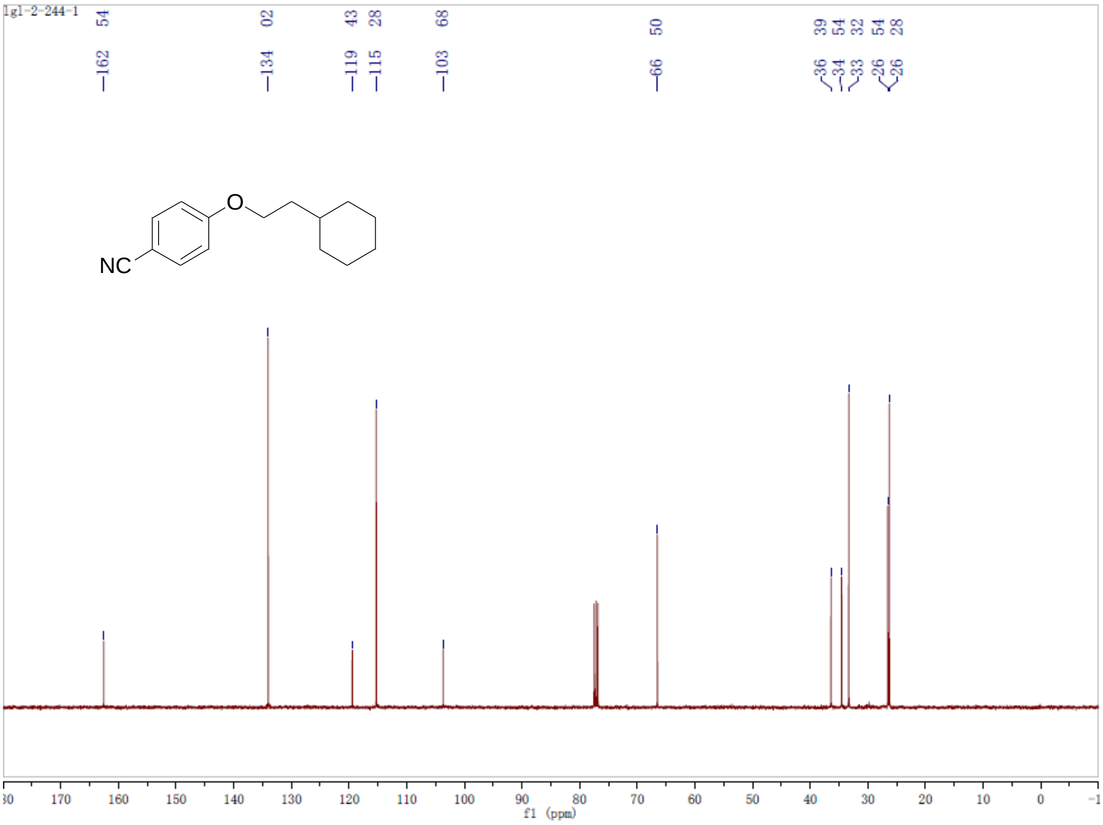

Supplementary Figure 35. ^{13}C **NMR spectra ($CDCl_3$, 100 MHz) of compound 4c**

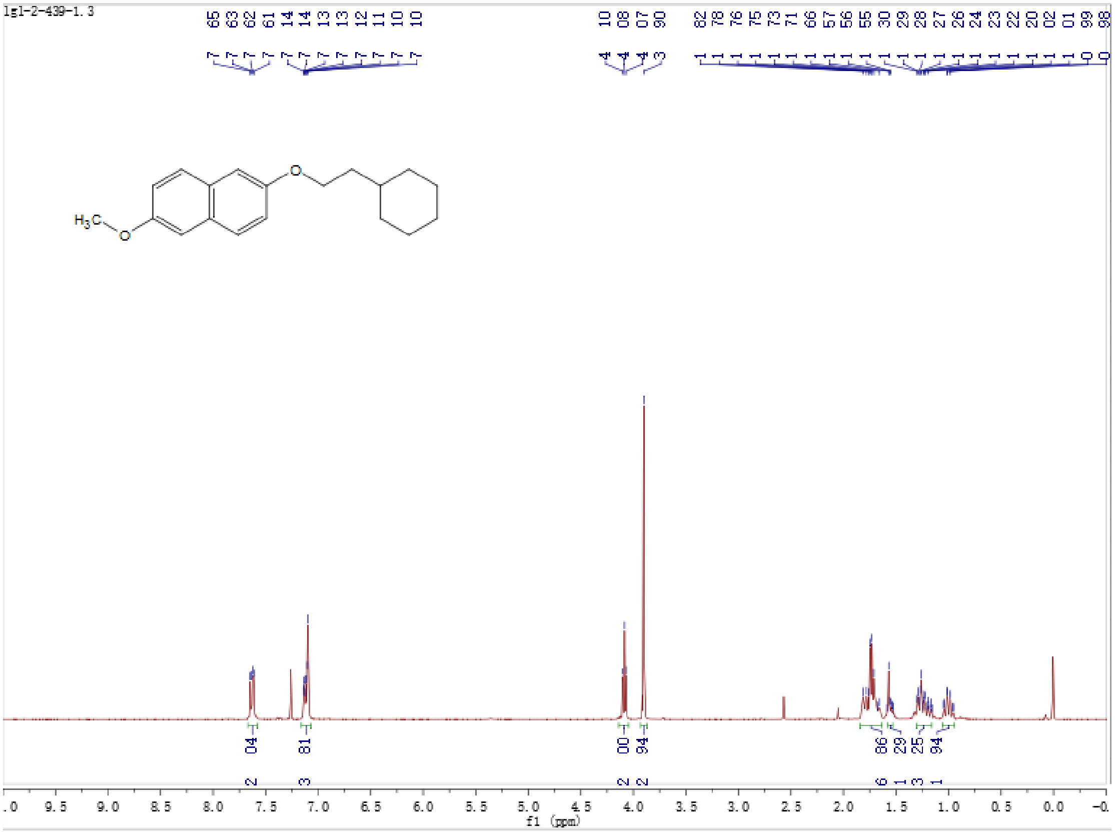

Supplementary Figure 36.^{1}H NMR spectra ($CDCl_3$, 400 MHz) of compound 4d

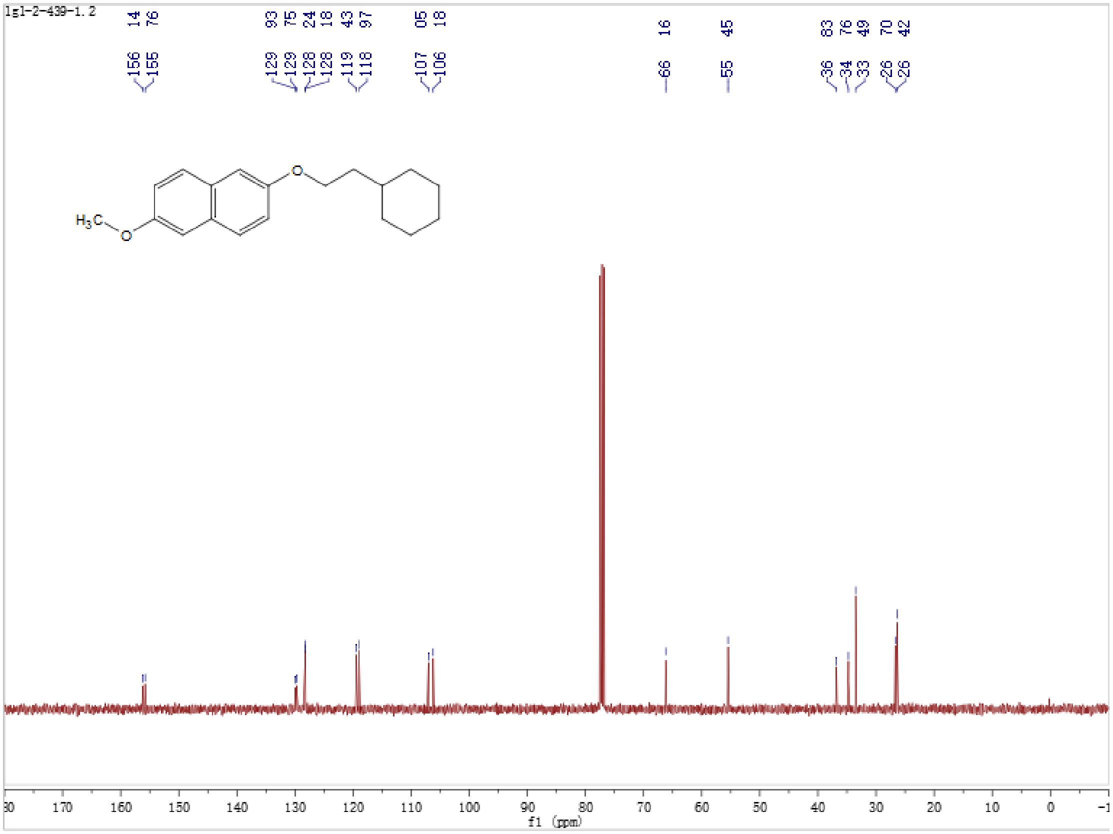

Supplementary Figure 37.^{13}C NMR spectra ($CDCl_3$, 100 MHz) of compound 4d

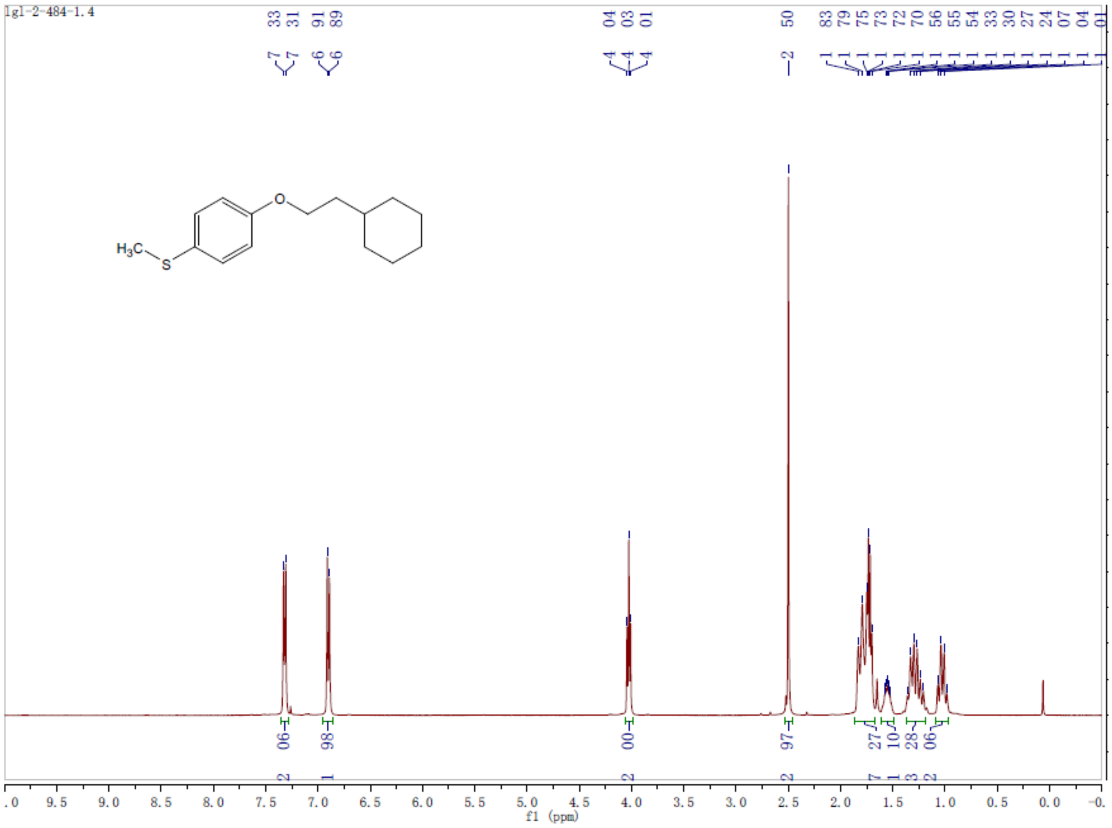

Supplementary Figure 38.^{1}H NMR spectra ($CDCl_3$, 400 MHz) of compound 4e

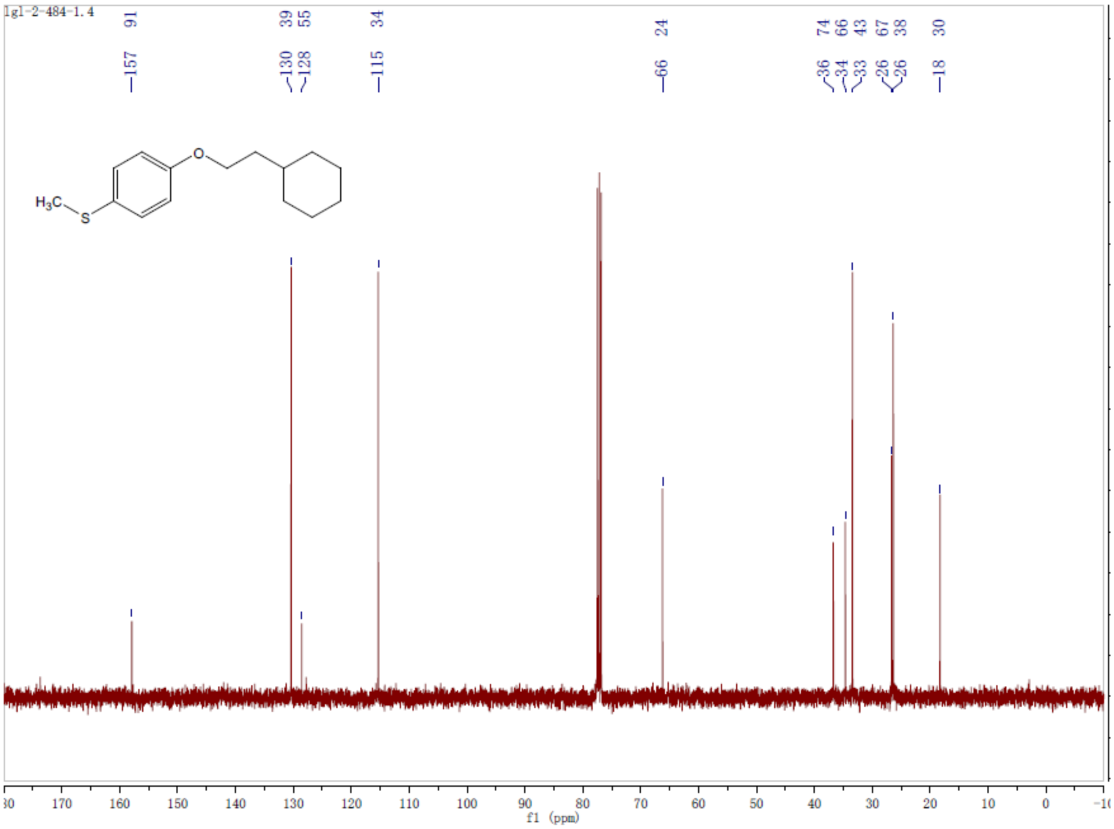

Supplementary Figure 39.^{13}C NMR spectra ($CDCl_3$, 100 MHz) of compound 4e

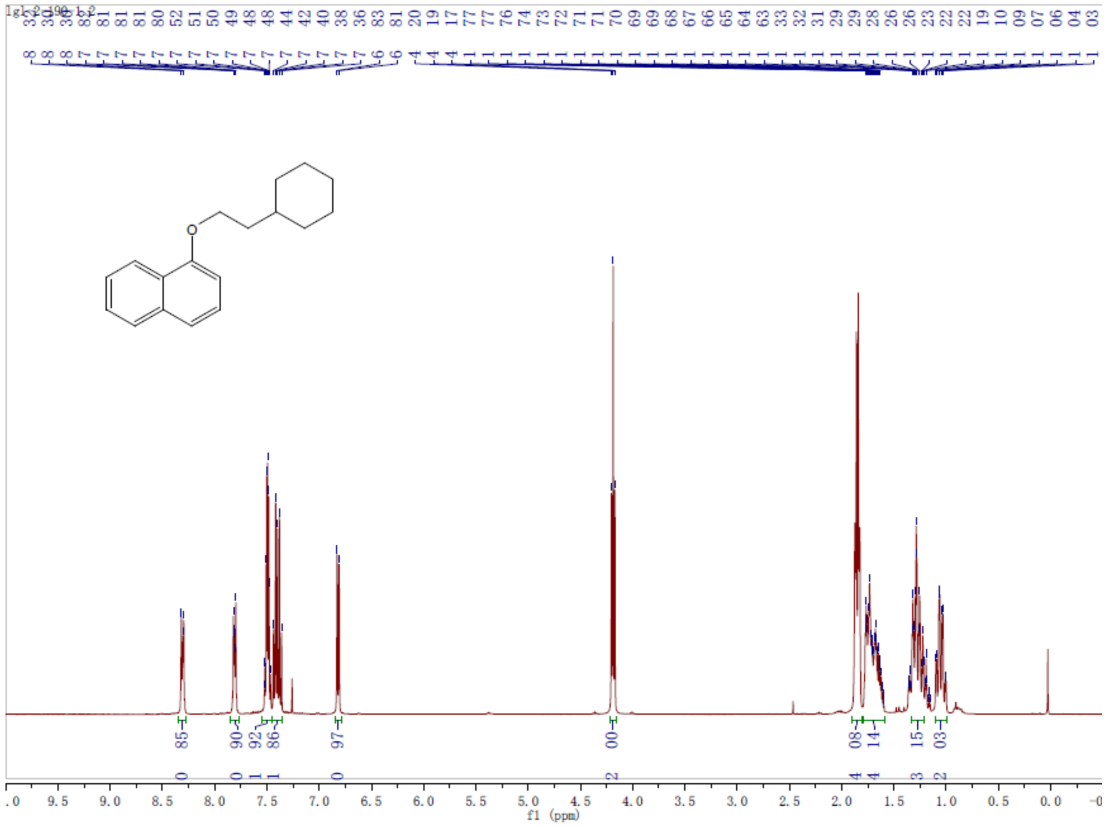

Supplementary Figure 40. ^{1}H **NMR spectra ($CDCl_3$, 400 MHz) of compound 4f**

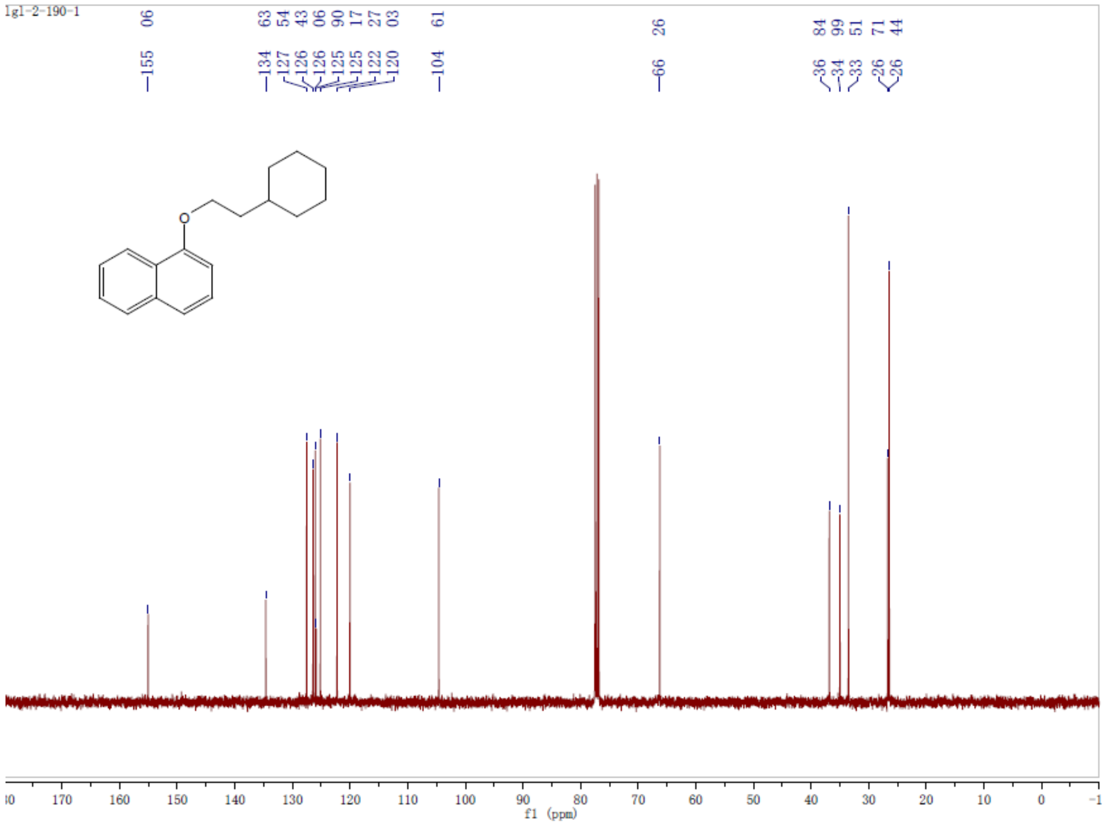

Supplementary Figure 41. ^{13}C **NMR spectra ($CDCl_3$, 100 MHz) of compound 4f**

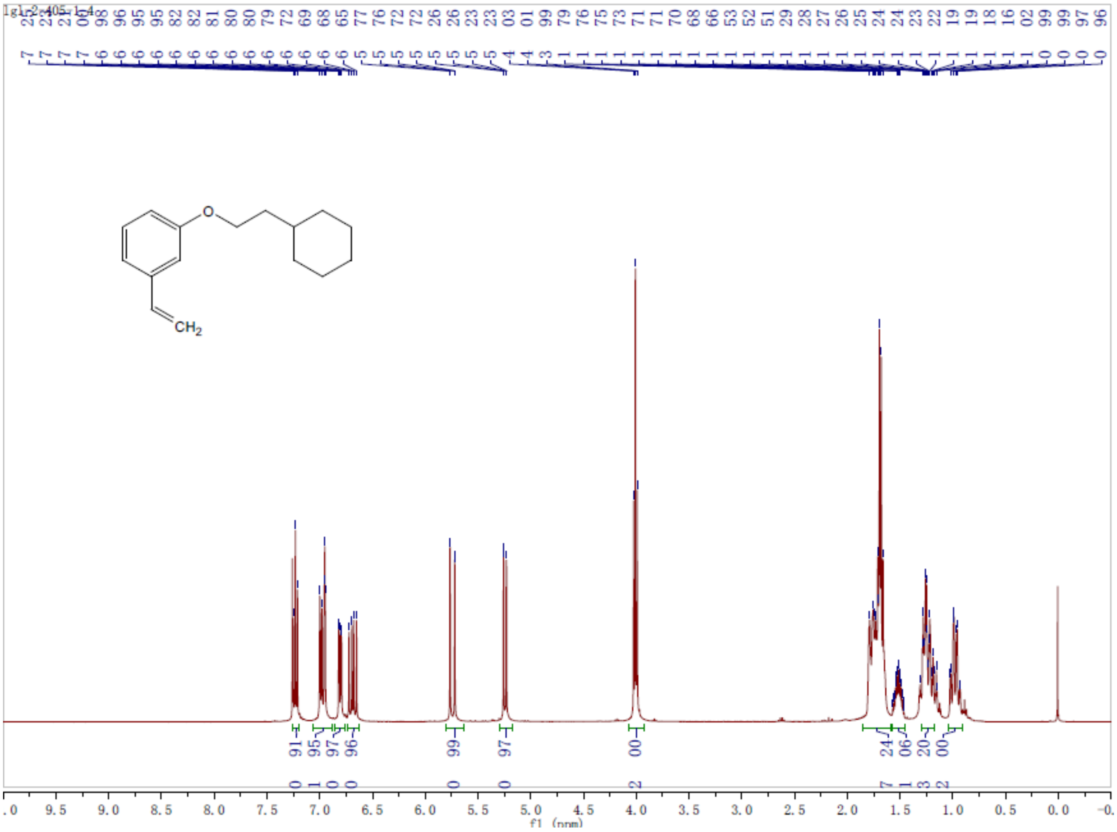

Supplementary Figure 42. ^{1}**H NMR spectra (CDCl$_3$, 400 MHz) of compound 4g**

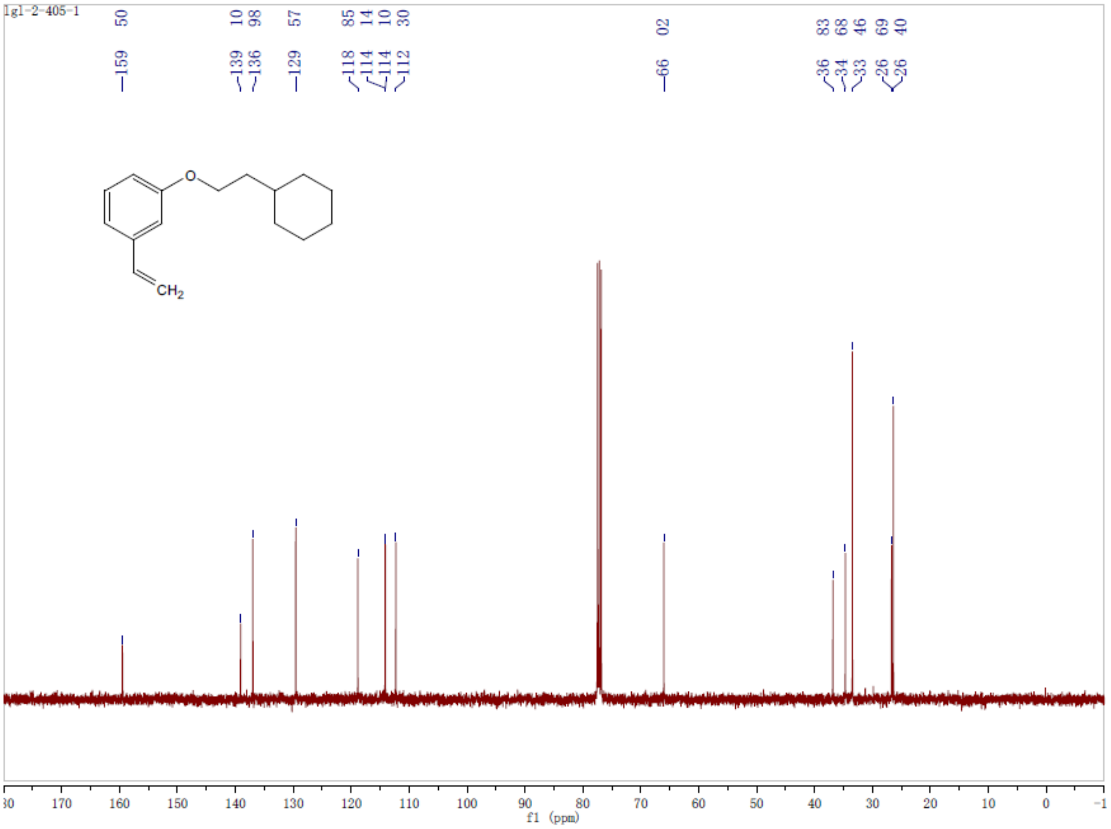

Supplementary Figure 43. ^{13}**C NMR spectra (CDCl$_3$, 100 MHz) of compound 4g**

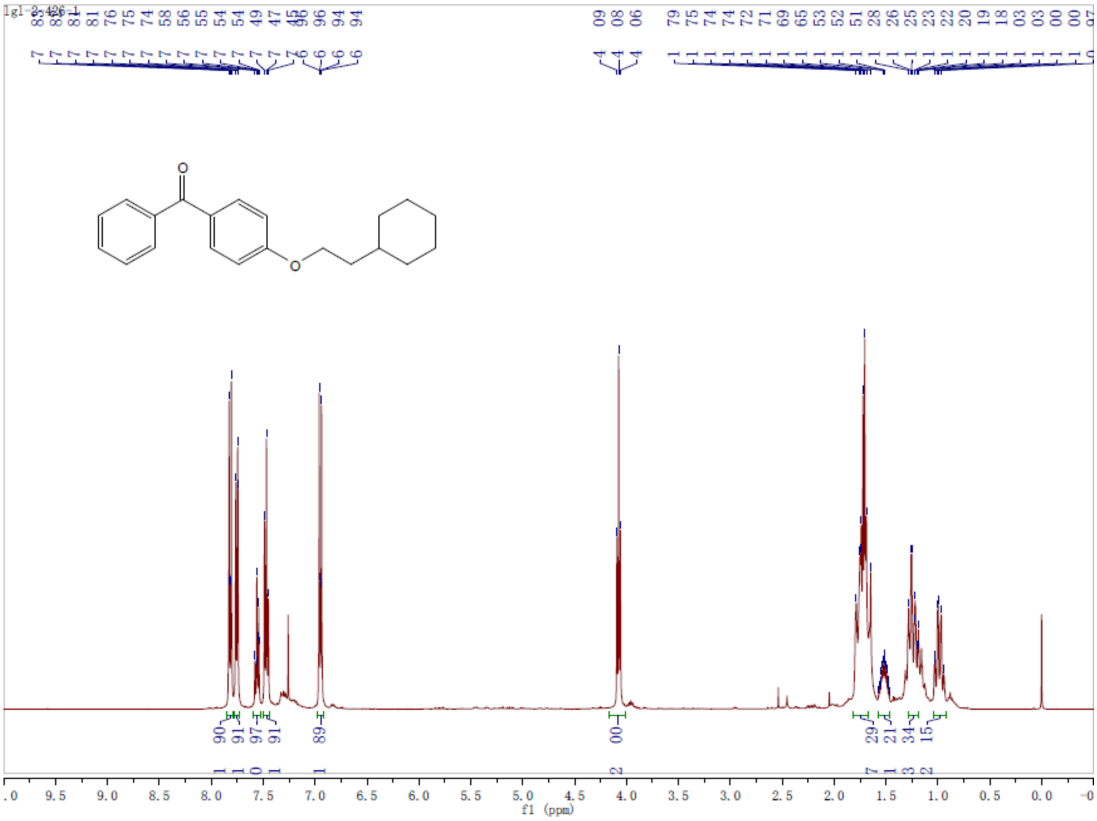

Supplementary Figure 44.^{1}H NMR spectra ($CDCl_3$, 400 MHz) of compound 4h

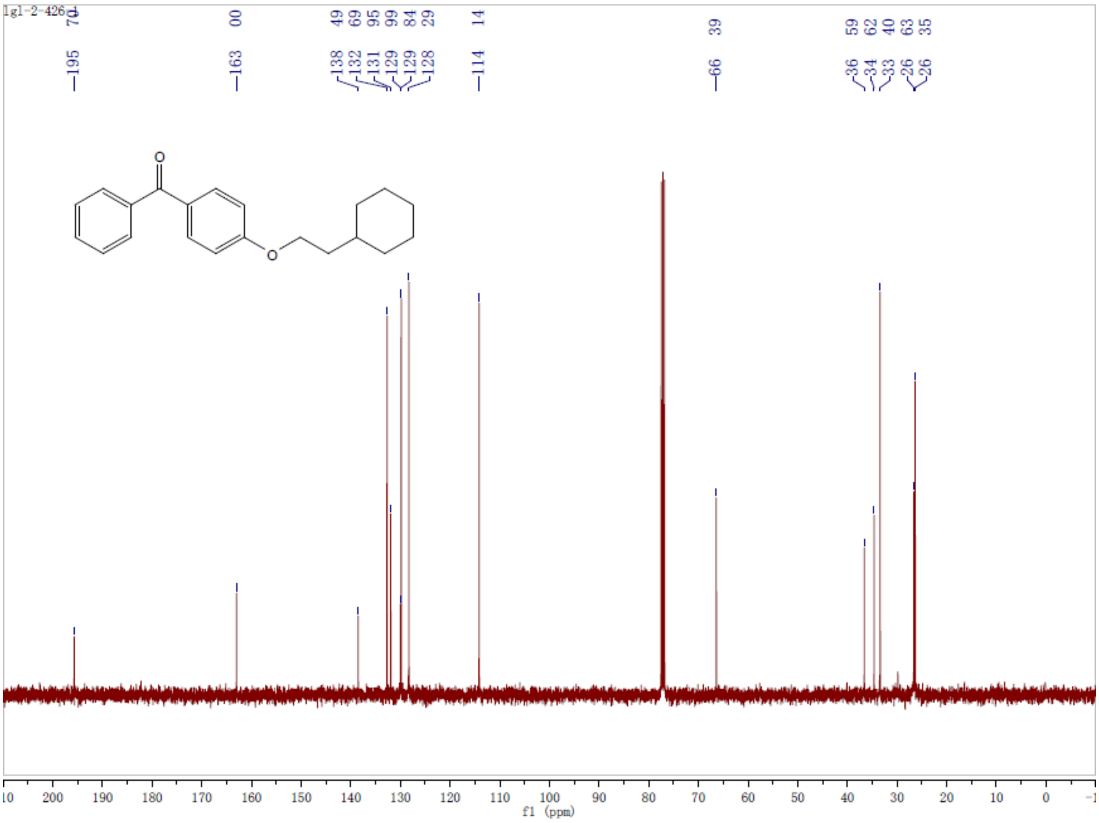

Supplementary Figure 45.^{13}C NMR spectra ($CDCl_3$, 100 MHz) of compound 4h

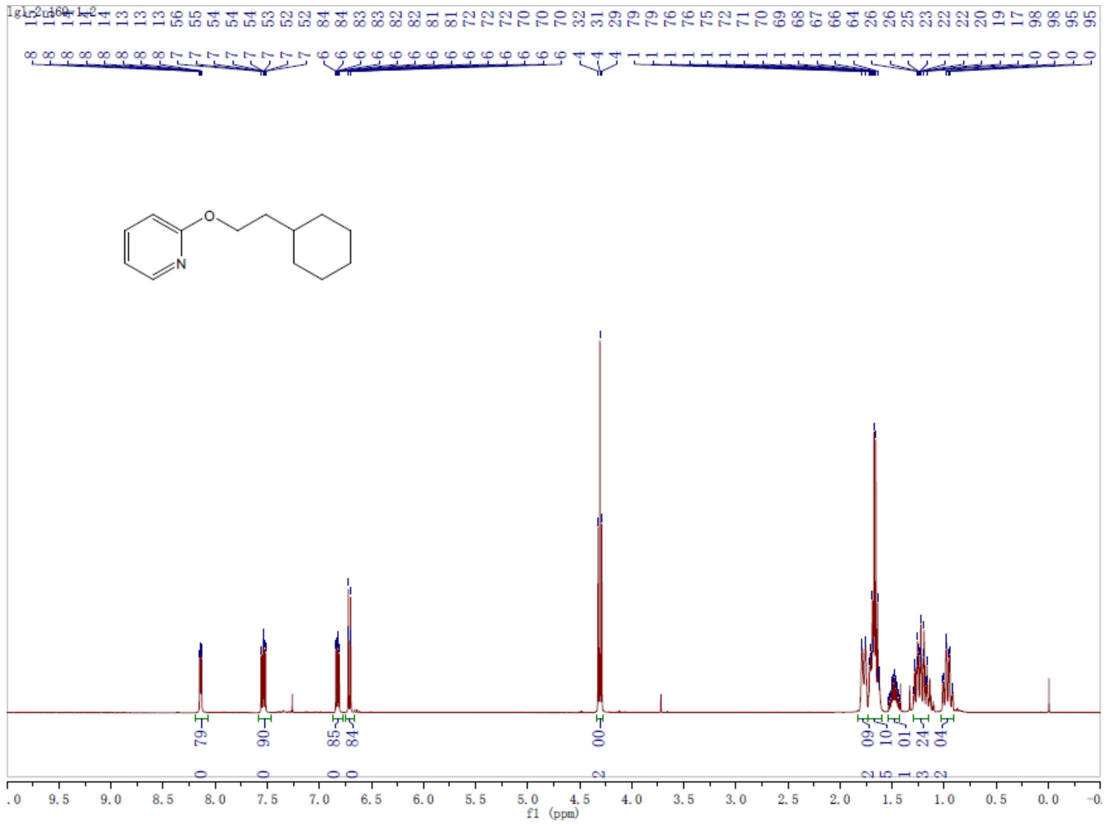

Supplementary Figure 46. ^{1}H NMR spectra ($CDCl_3$, 400 MHz) of compound 4i

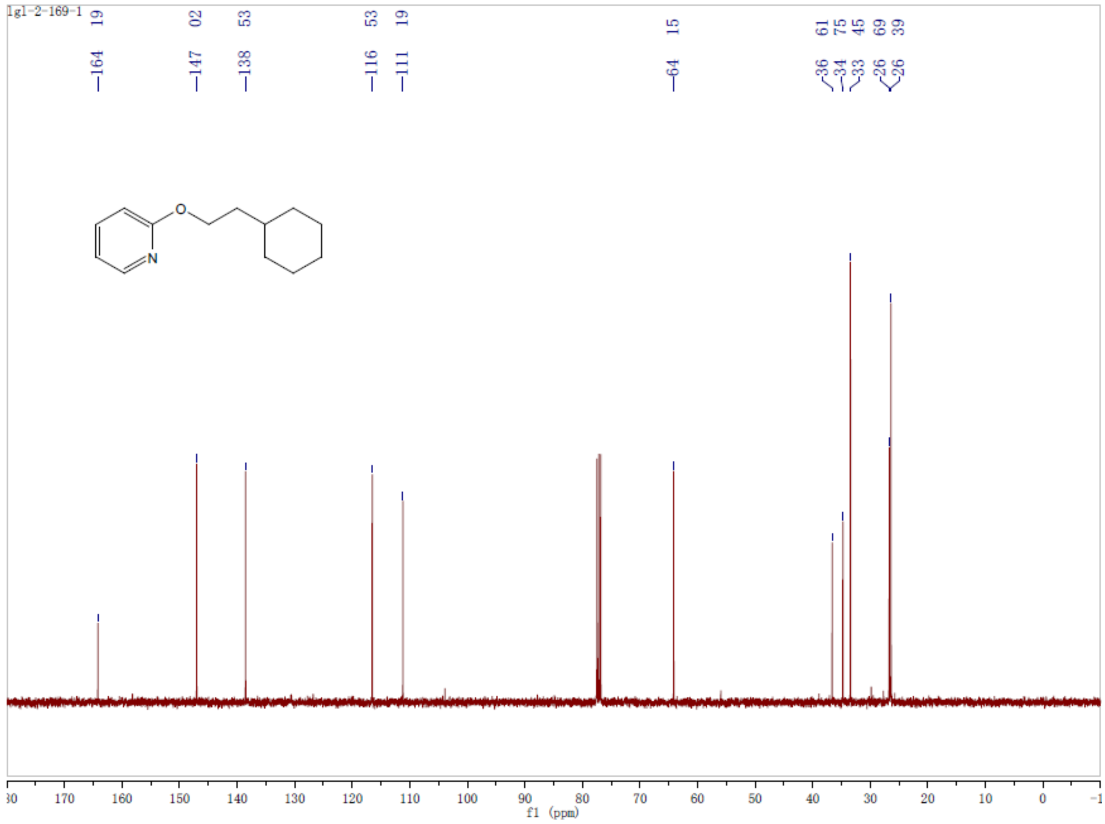

Supplementary Figure 47. ^{13}C NMR spectra ($CDCl_3$, 100 MHz) of compound 4i

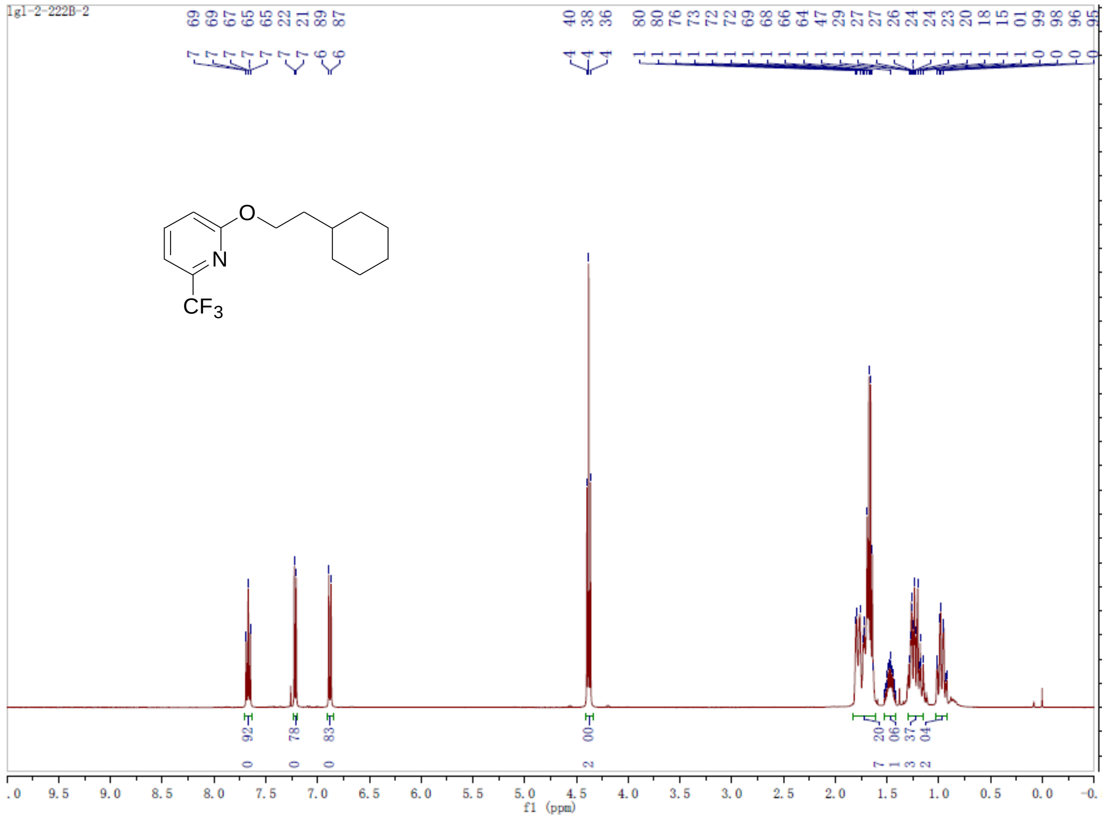

Supplementary Figure 48.^{1}H NMR spectra ($CDCl_3$, 400 MHz) of compound 4j

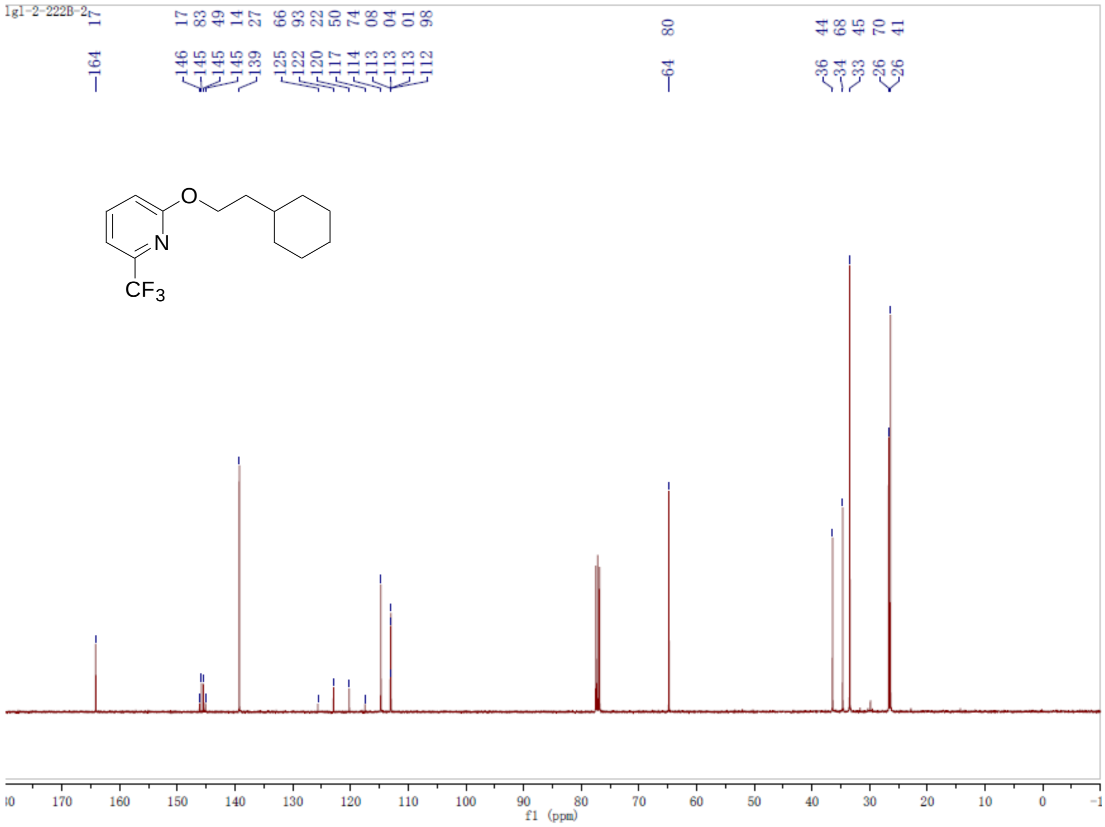

Supplementary Figure 49.^{13}C NMR spectra ($CDCl_3$, 100 MHz) of compound 4j

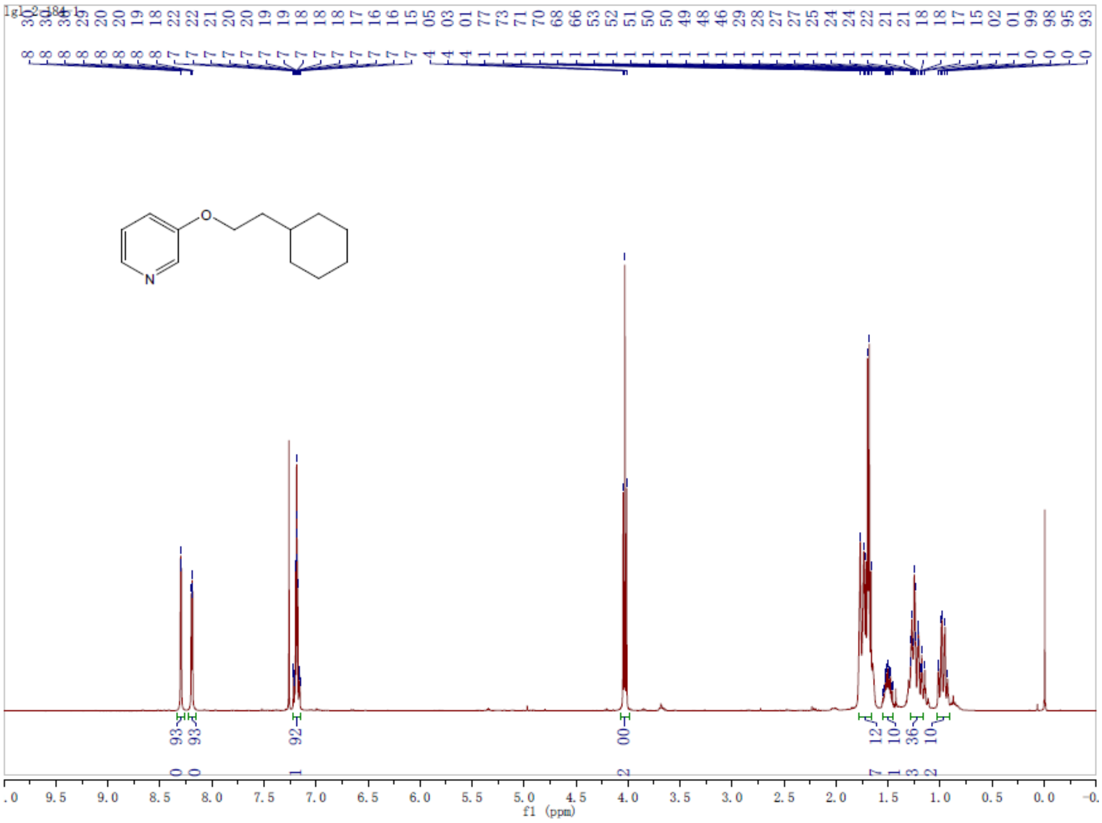

Supplementary Figure 50. ^{1}**H NMR spectra (CDCl$_3$, 400 MHz) of compound 4k**

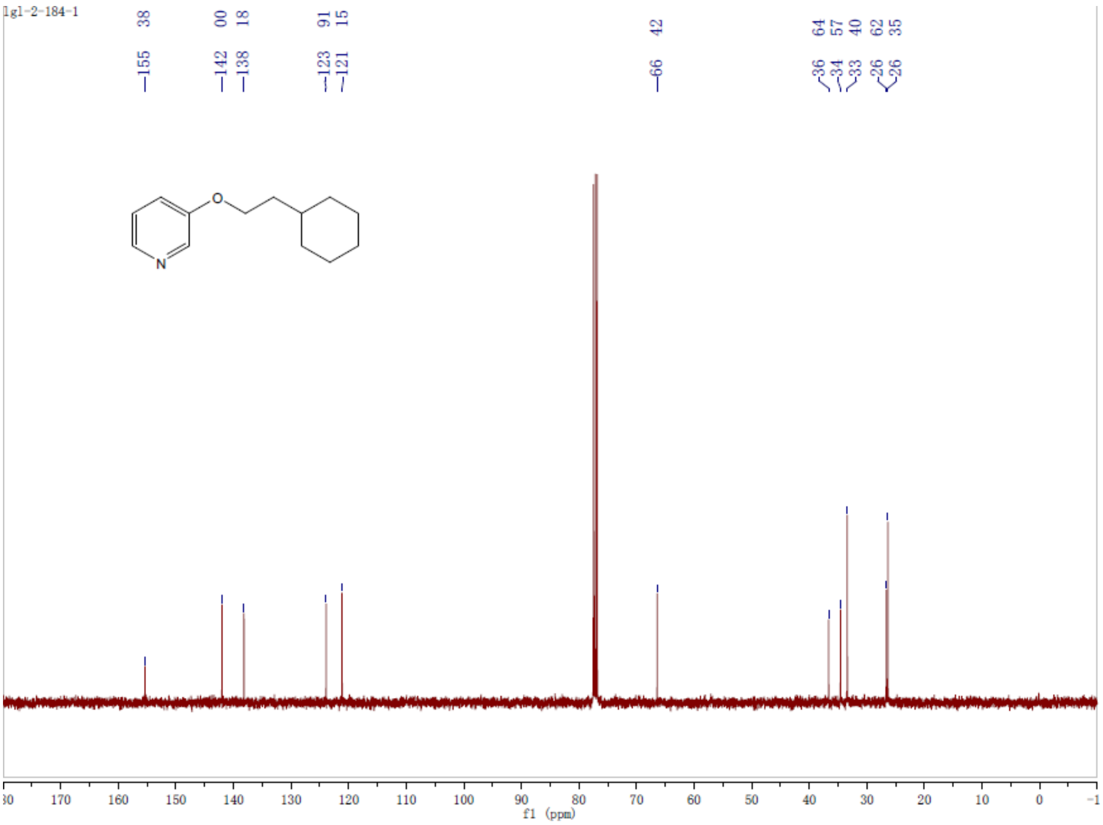

Supplementary Figure 51. ^{13}**C NMR spectra (CDCl$_3$, 100 MHz) of compound 4k**

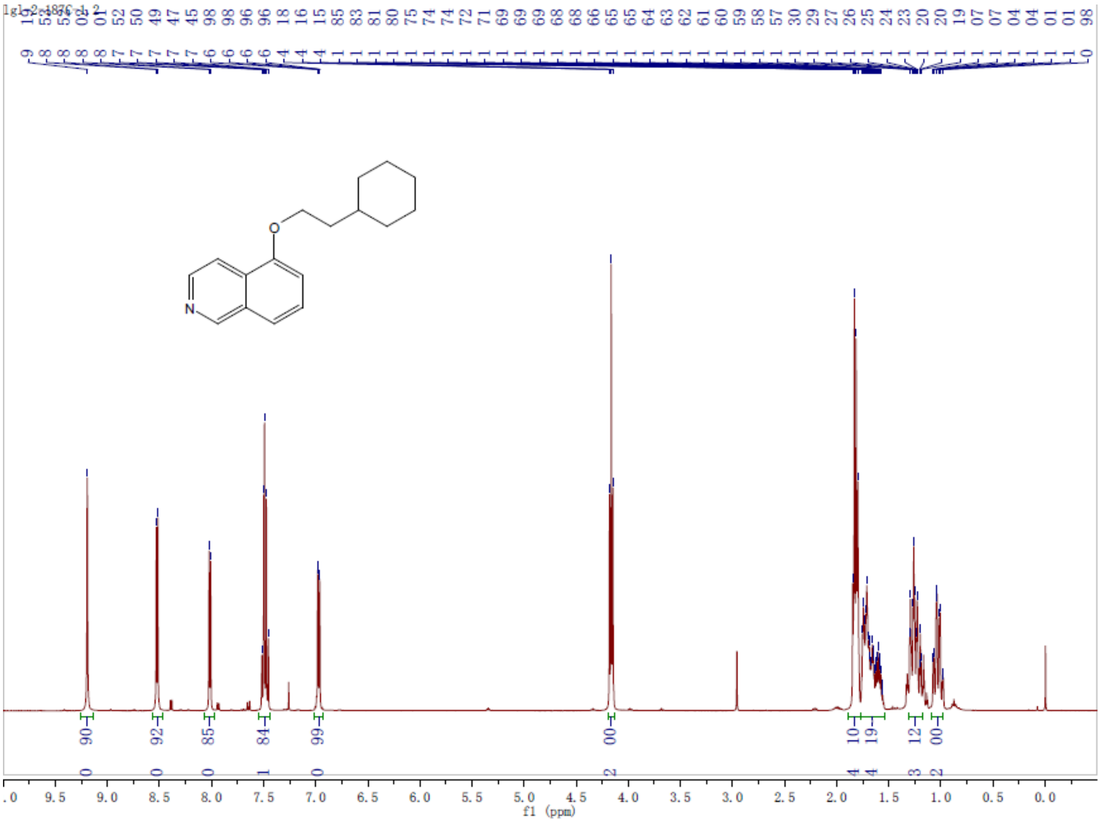

Supplementary Figure 52.^{1}H NMR spectra ($CDCl_3$, 400 MHz) of compound 4l

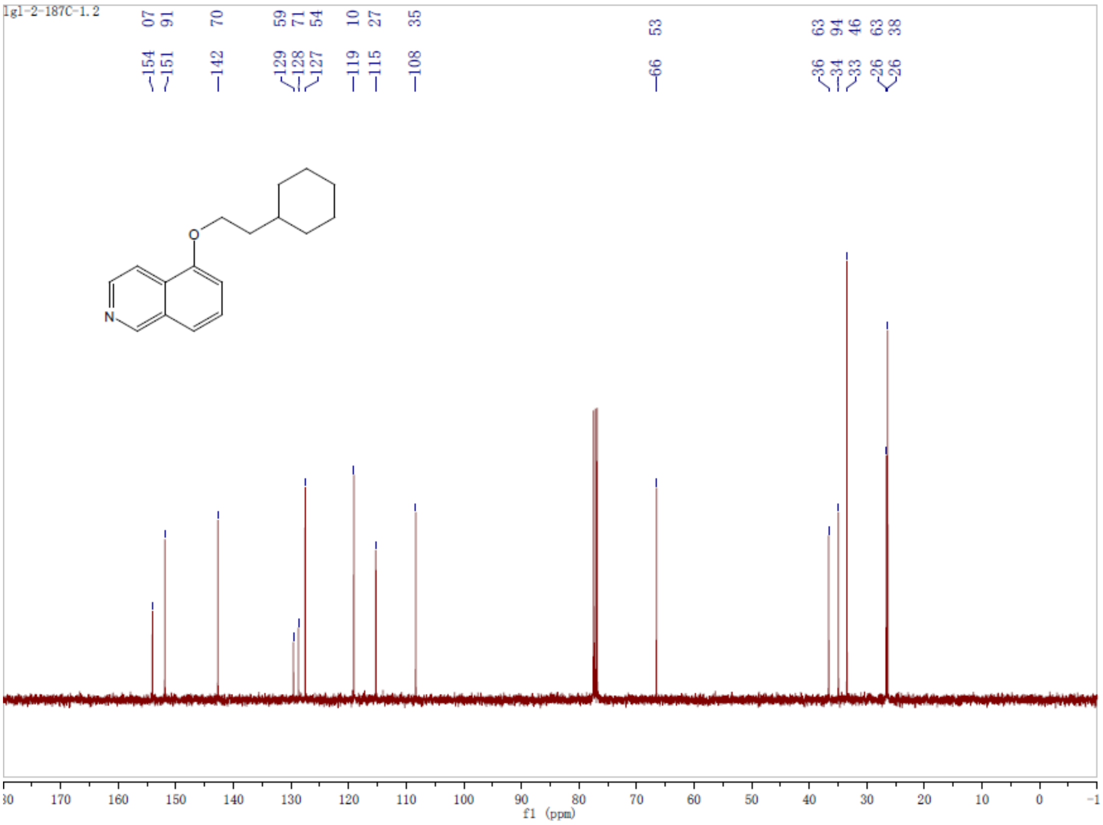

Supplementary Figure 53.^{13}C NMR spectra ($CDCl_3$, 100 MHz) of compound 4l

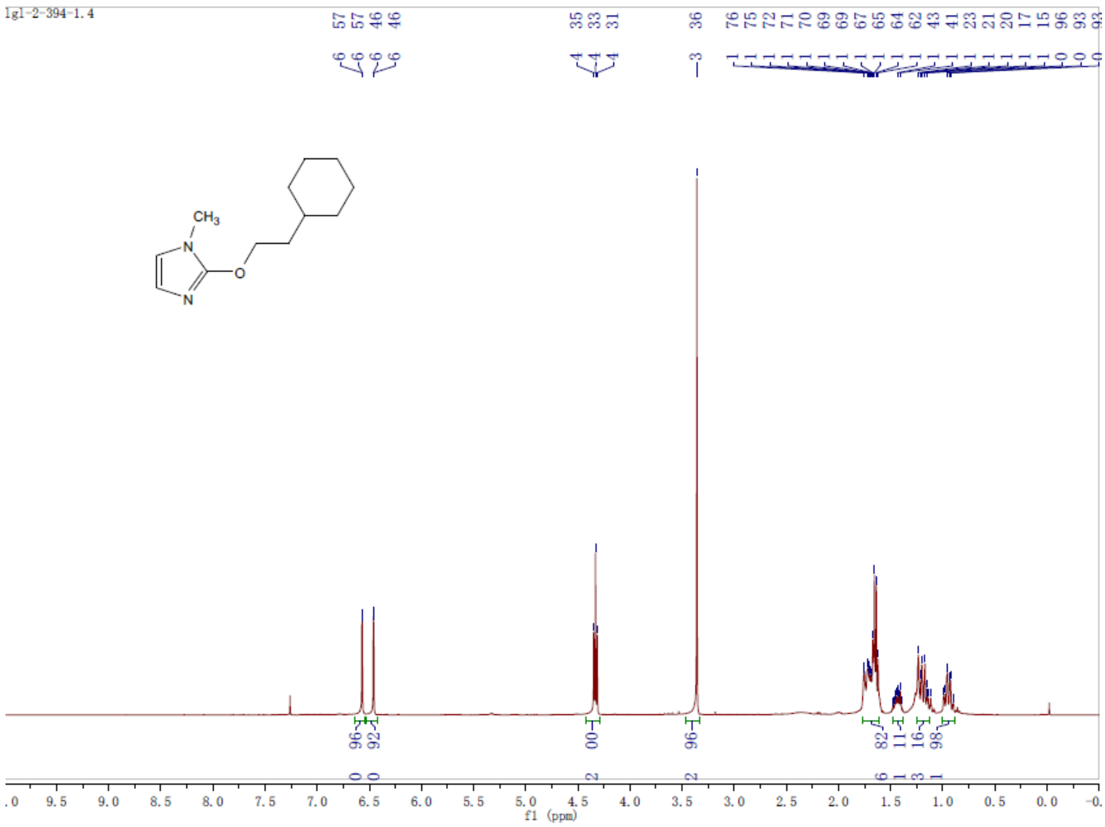

Supplementary Figure 54.^{1}H NMR spectra ($CDCl_3$, 400 MHz) of compound 4m

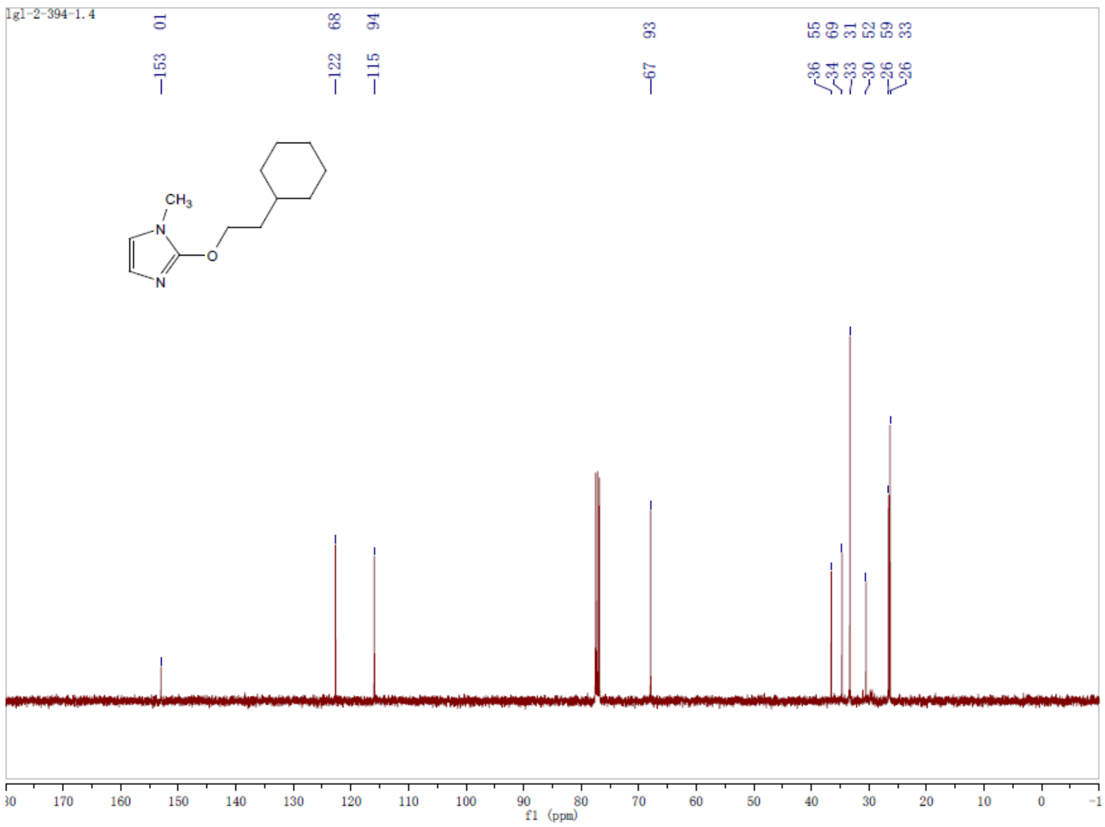

Supplementary Figure 55.^{13}C NMR spectra ($CDCl_3$, 100 MHz) of compound 4m

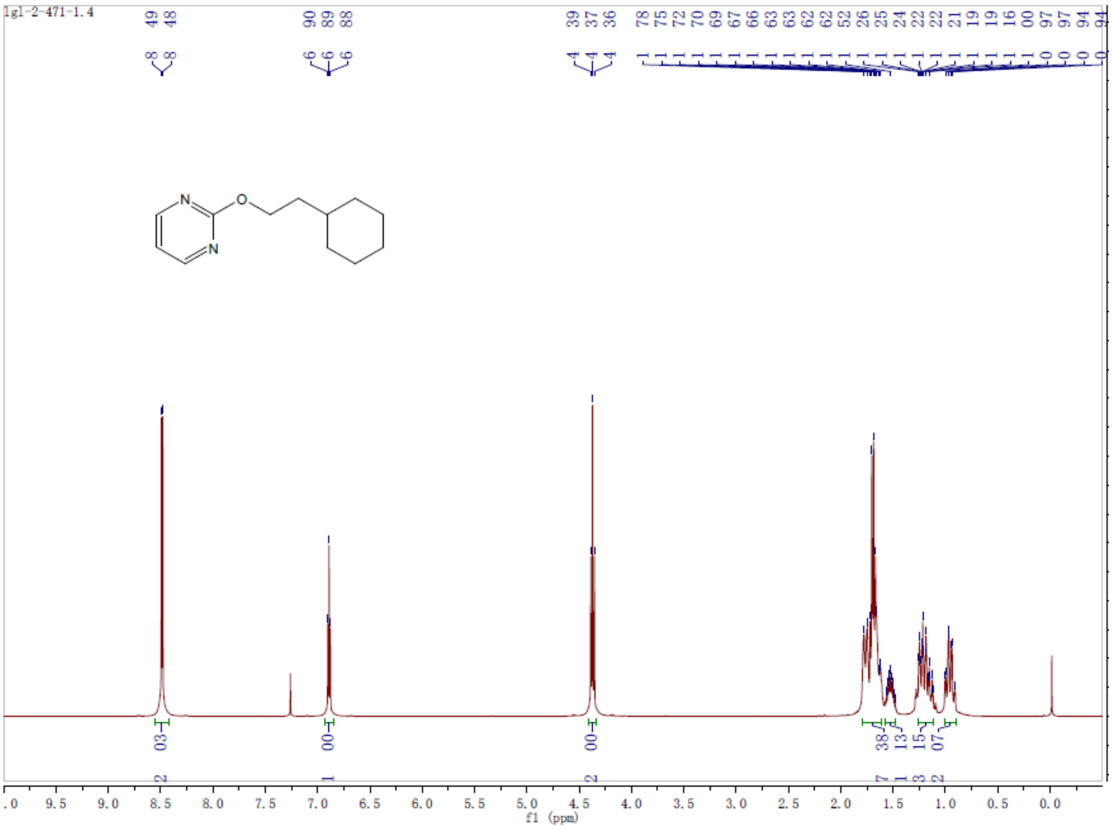

Supplementary Figure 56.^{1}H NMR spectra ($CDCl_3$, 400 MHz) of compound 4n

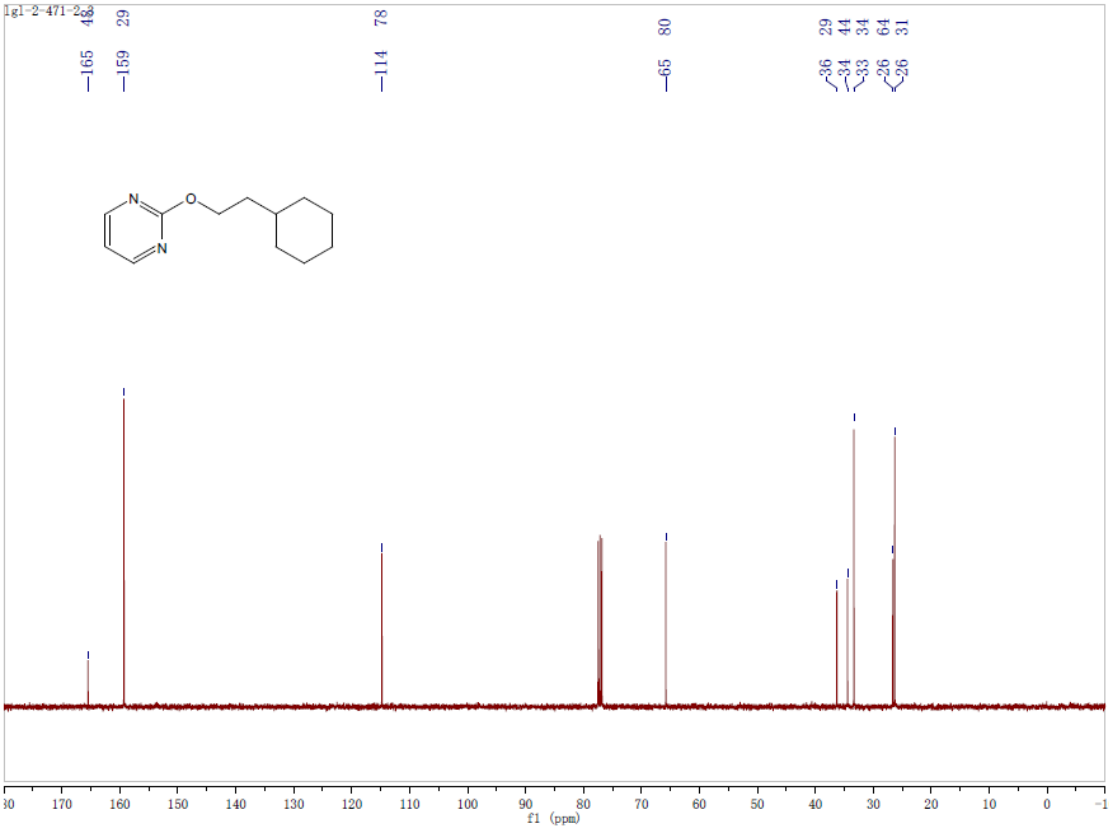

Supplementary Figure 57.^{13}C NMR spectra ($CDCl_3$, 100 MHz) of compound 4n

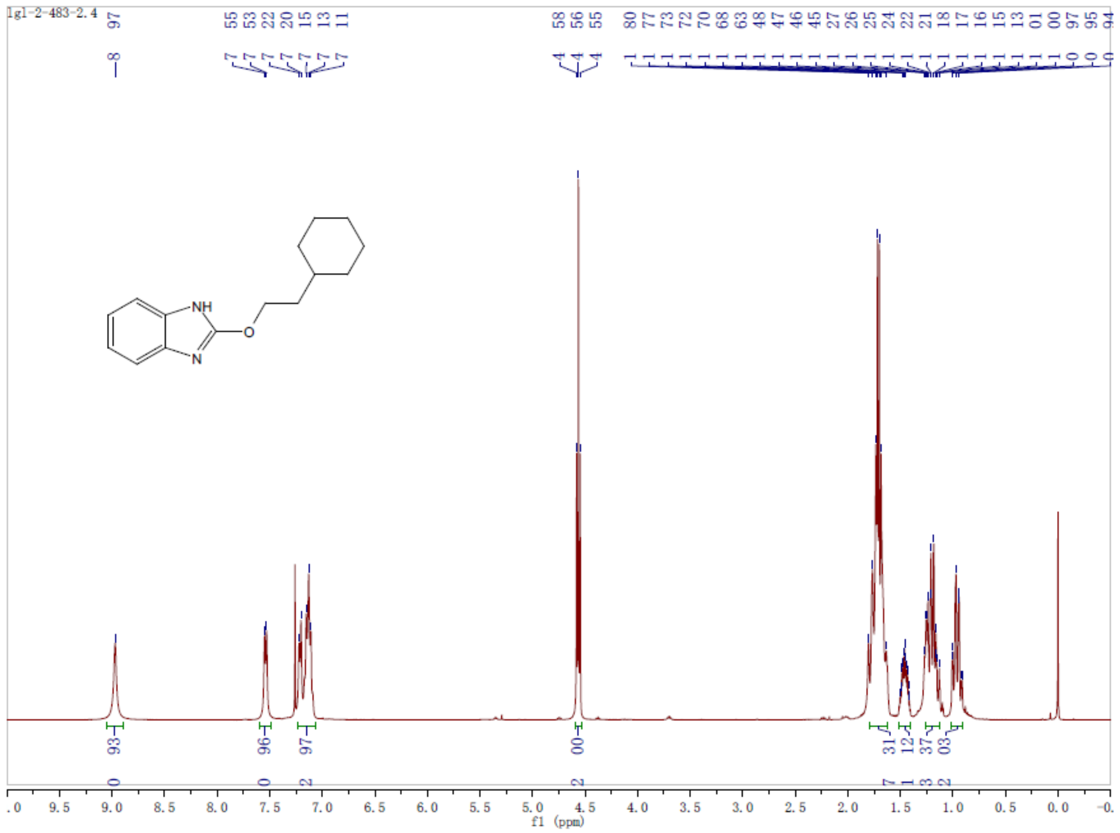

Supplementary Figure 58.[1]**H NMR spectra ($CDCl_3$, 400 MHz) of compound 4o**

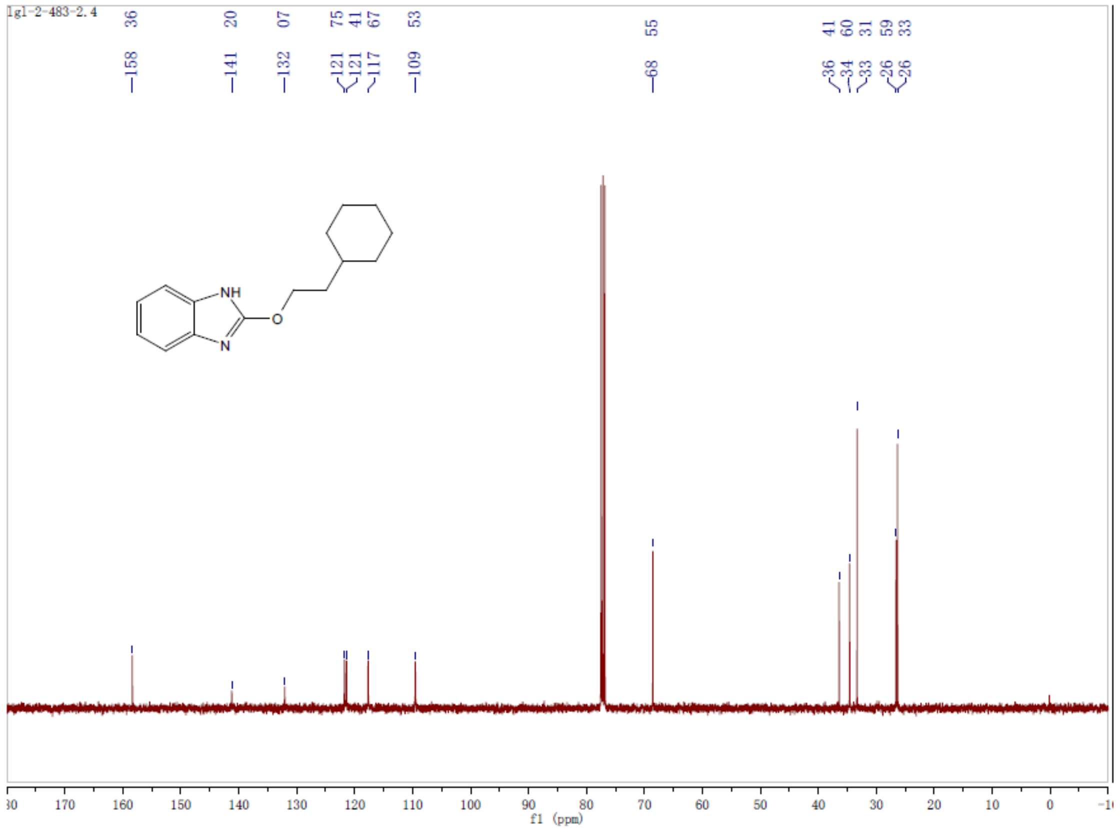

Supplementary Figure 59.[13]**C NMR spectra ($CDCl_3$, 100 MHz) of compound 4o**

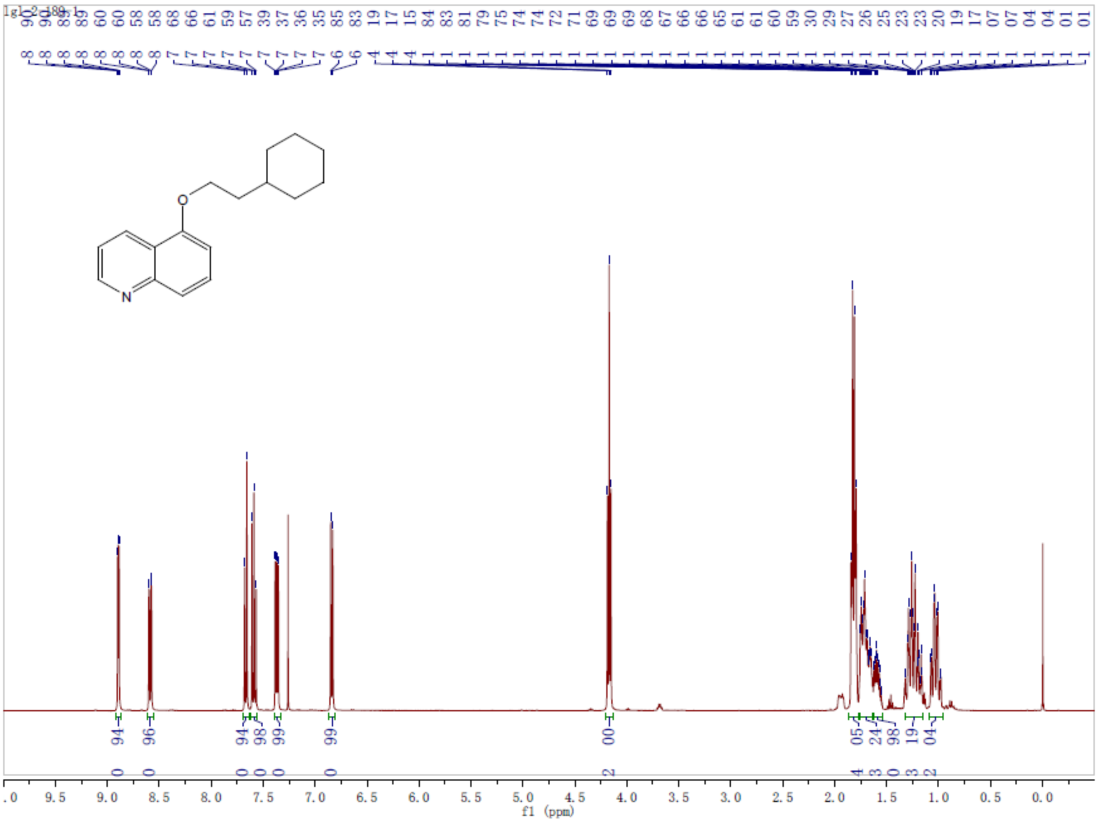

Supplementary Figure 60.^{1}H NMR spectra ($CDCl_3$, 400 MHz) of compound 4p

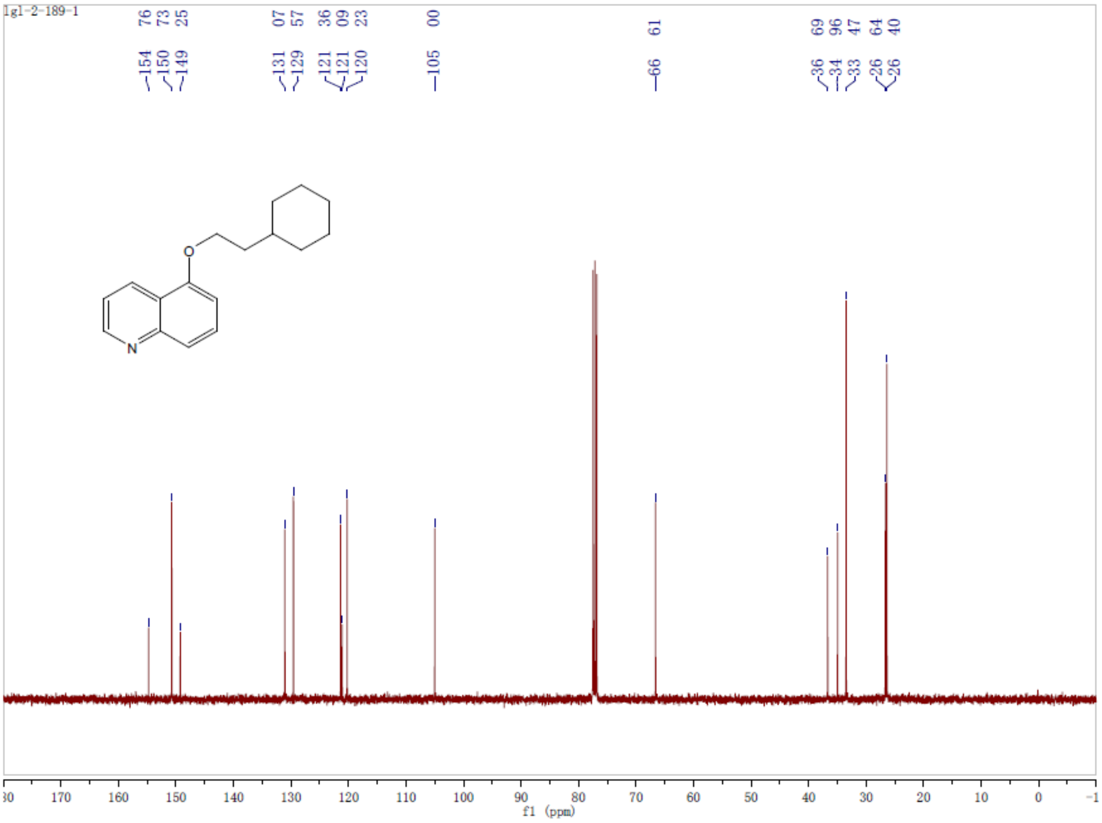

Supplementary Figure 61.^{13}C NMR spectra ($CDCl_3$, 100 MHz) of compound 4p

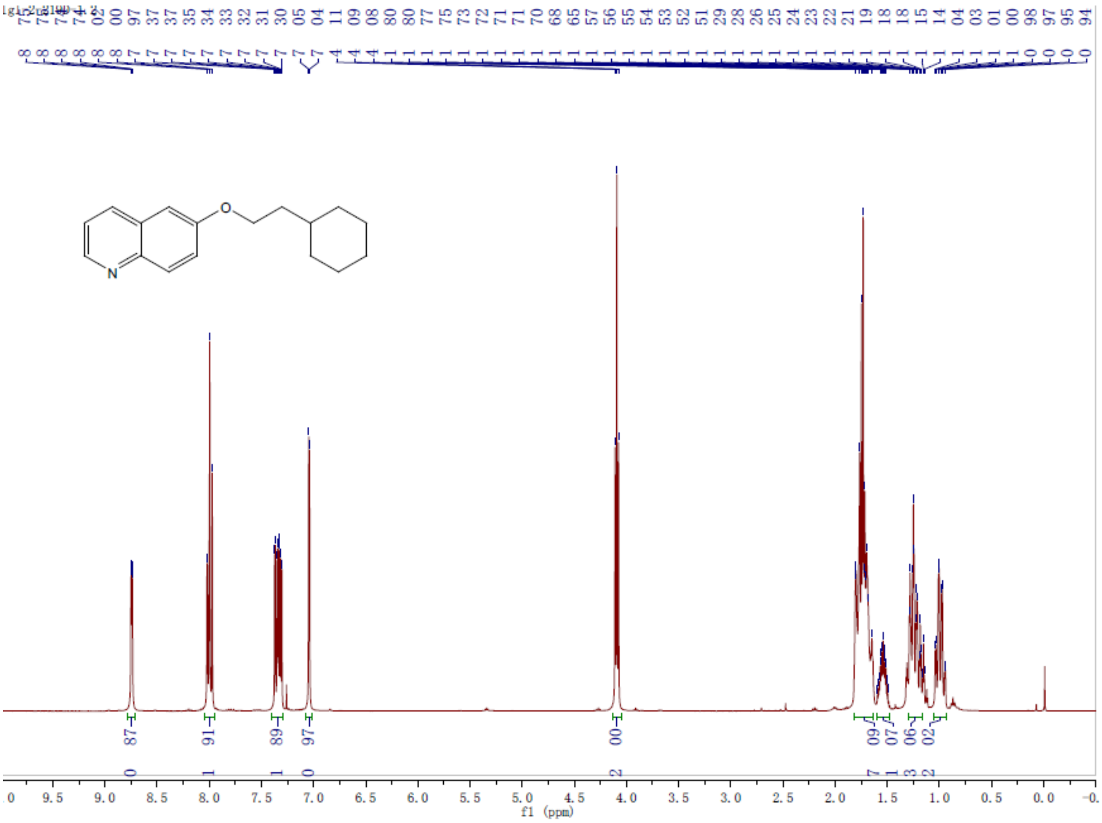

Supplementary Figure 62.^{1}H NMR spectra ($CDCl_3$, 400 MHz) of compound 4q

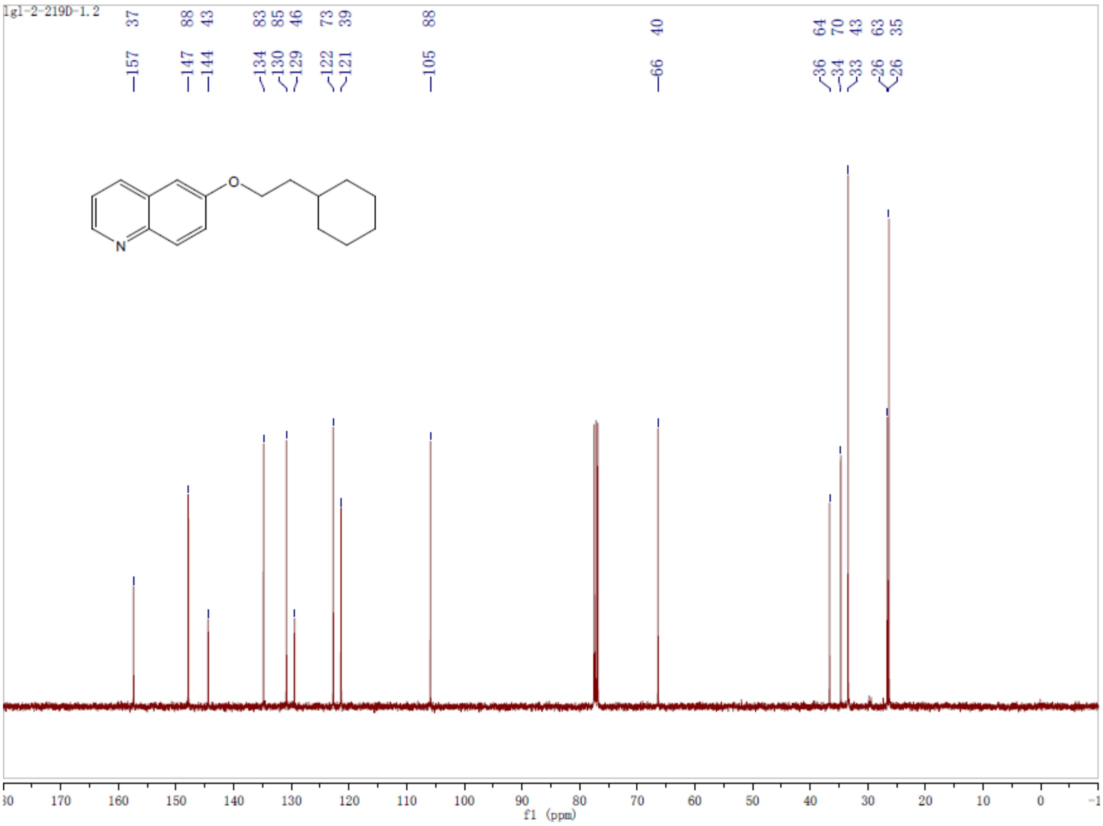

Supplementary Figure 63.^{13}C NMR spectra ($CDCl_3$, 100 MHz) of compound 4q

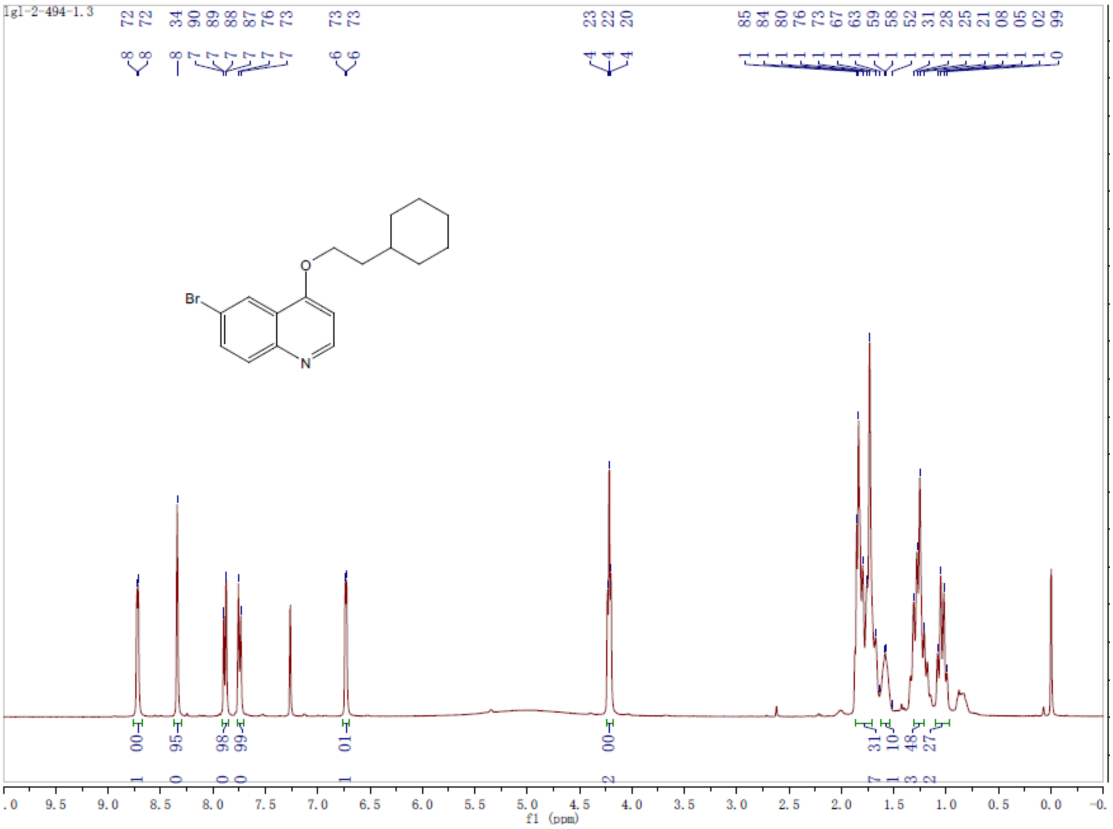

Supplementary Figure 64.^{1}H NMR spectra ($CDCl_3$, 400 MHz) of compound 4r

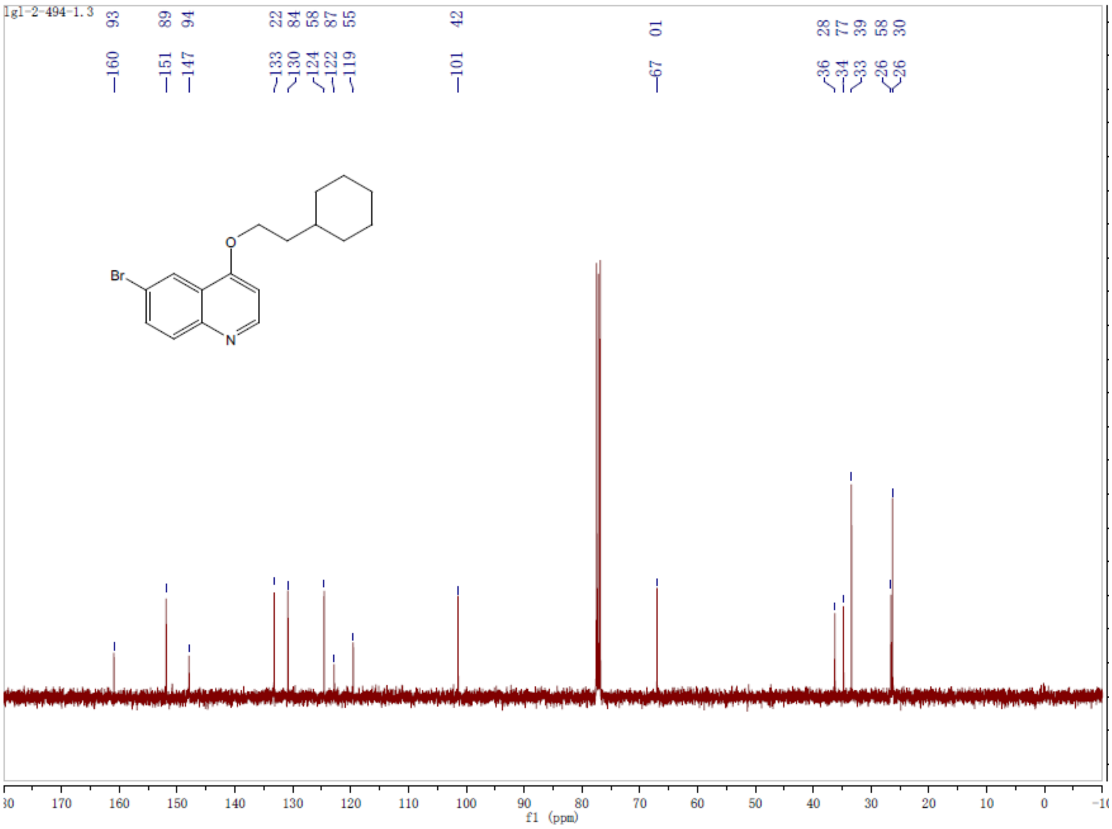

Supplementary Figure 65.^{13}C NMR spectra ($CDCl_3$, 100 MHz) of compound 4r

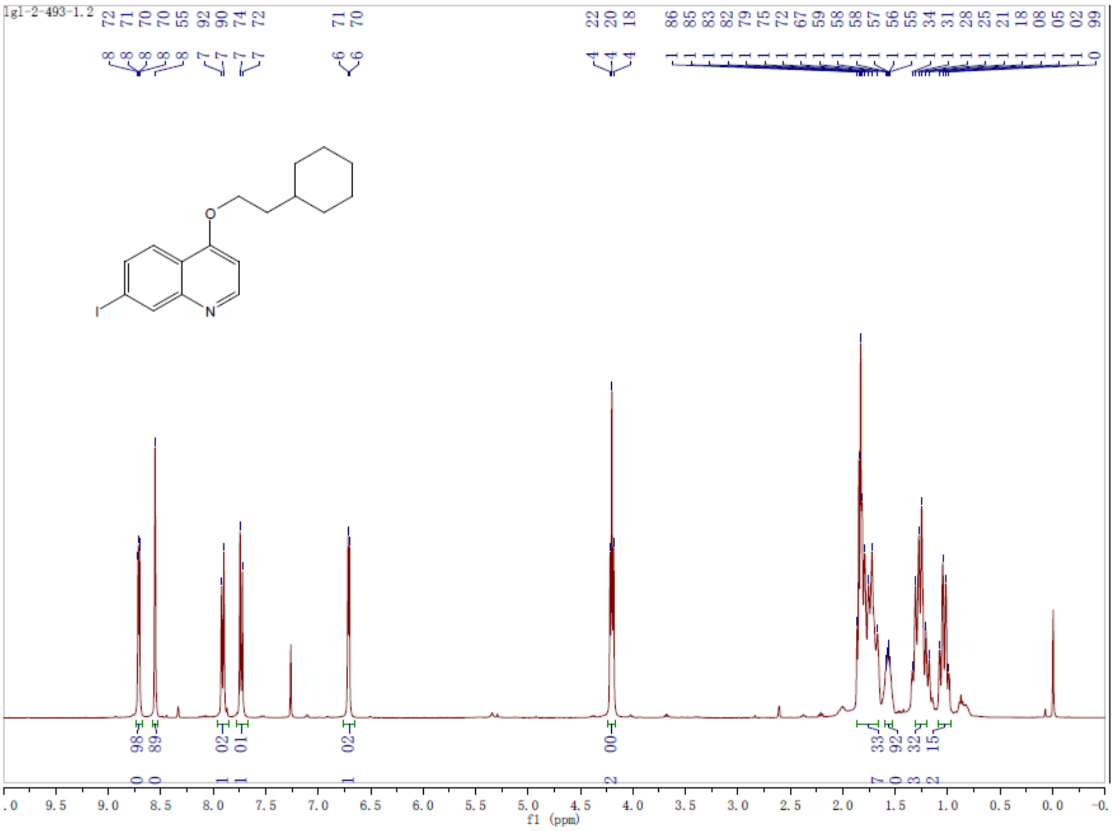

Supplementary Figure 66.^{1}H NMR spectra ($CDCl_3$, 400 MHz) of compound 4s

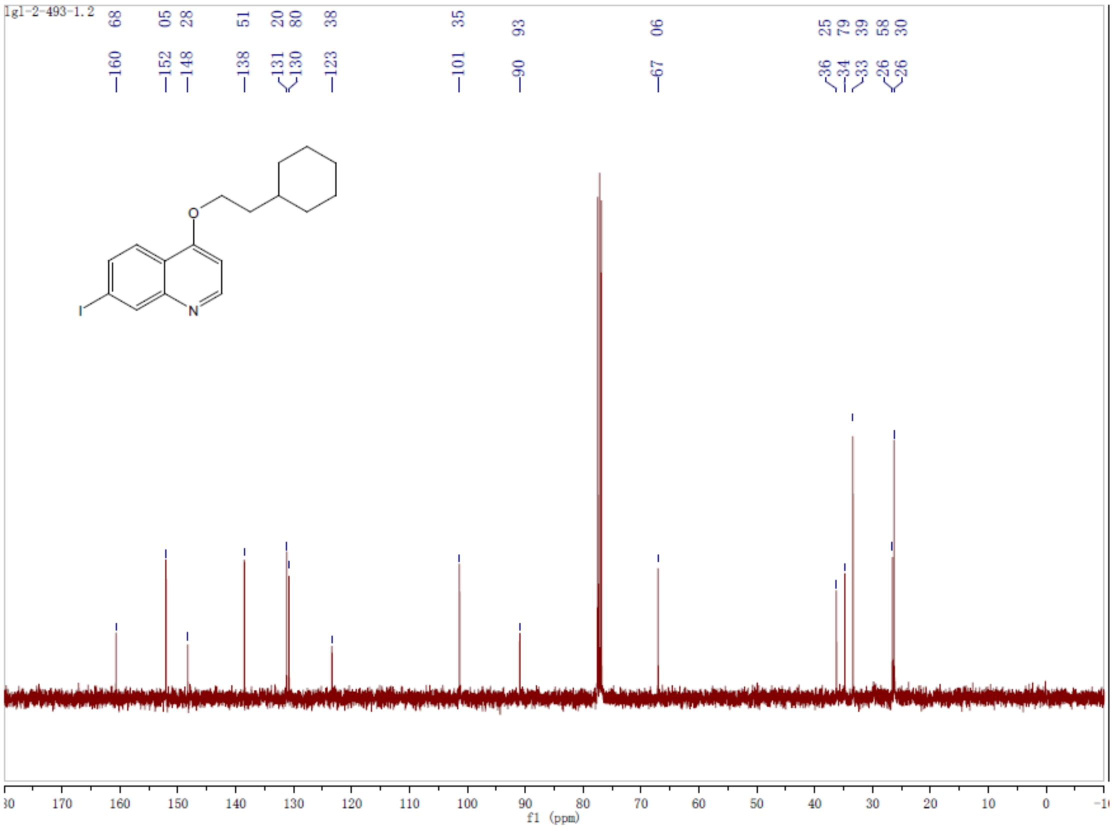

Supplementary Figure 67.^{13}C NMR spectra ($CDCl_3$, 100 MHz) of compound 4s

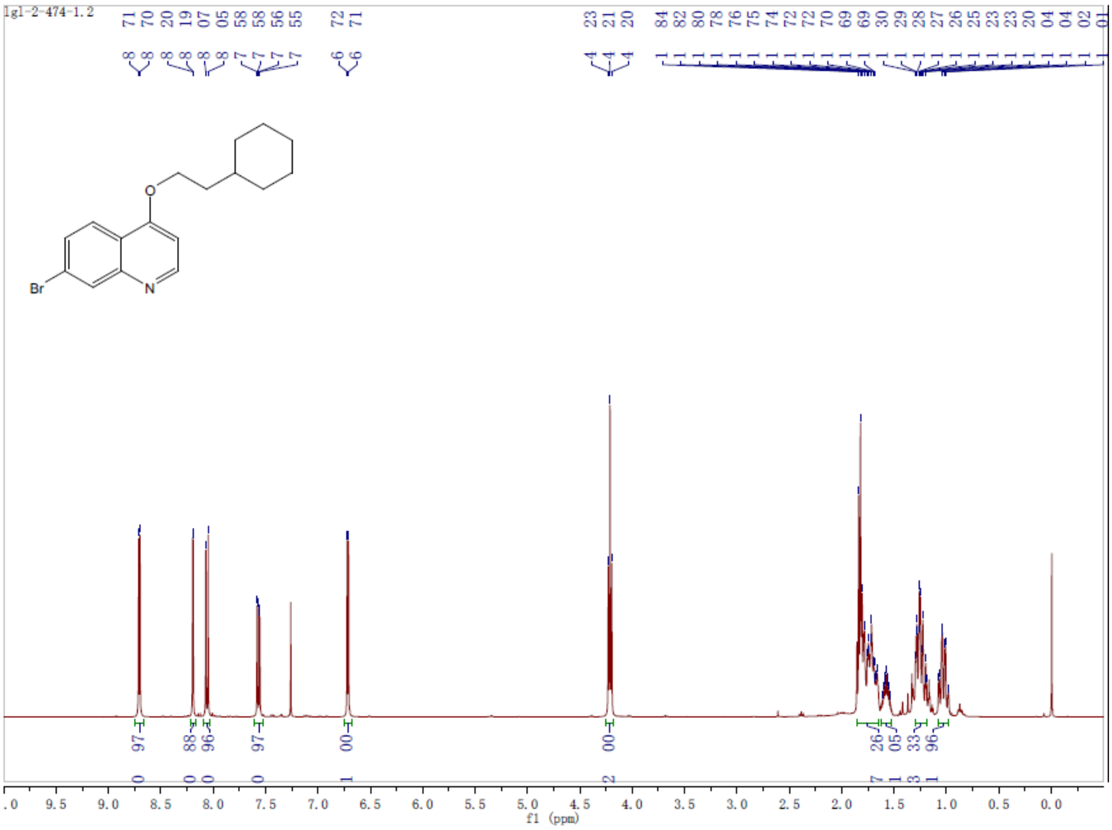

Supplementary Figure 68. ^{1}H **NMR spectra (** $CDCl_3$ **, 400 MHz) of compound 4t**

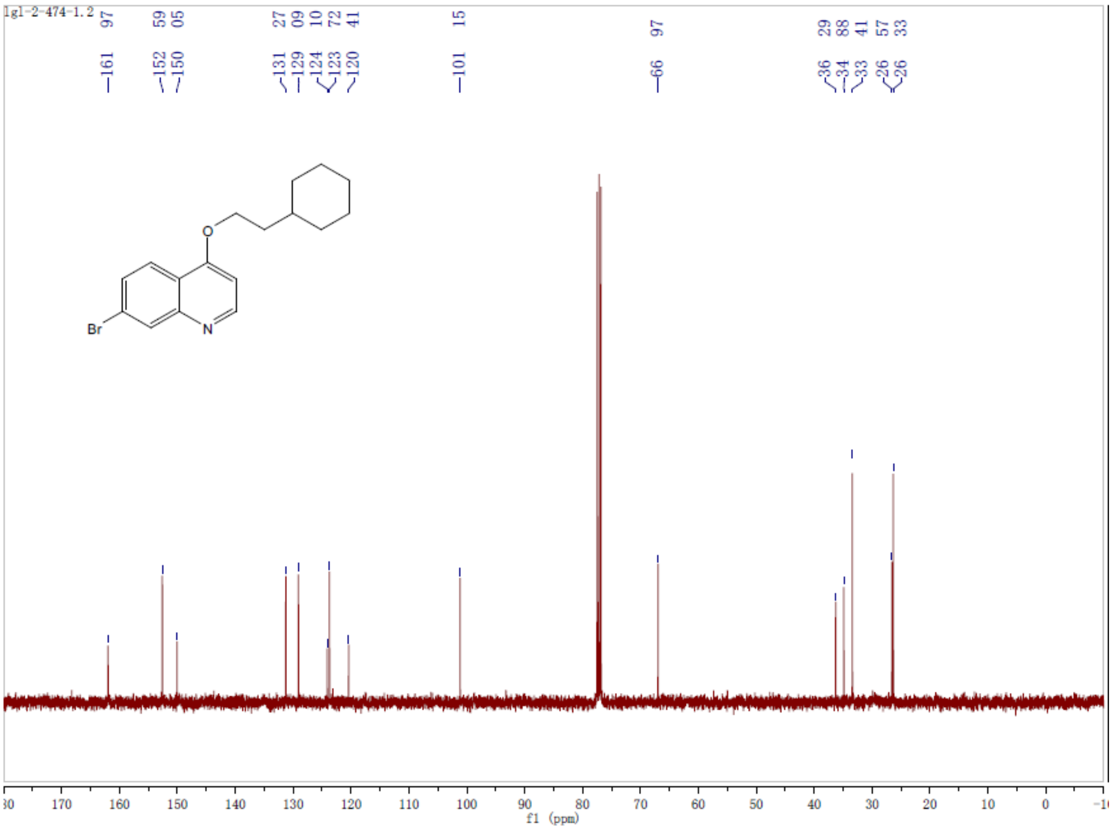

Supplementary Figure 69. ^{13}C **NMR spectra (** $CDCl_3$ **, 100 MHz) of compound 4t**

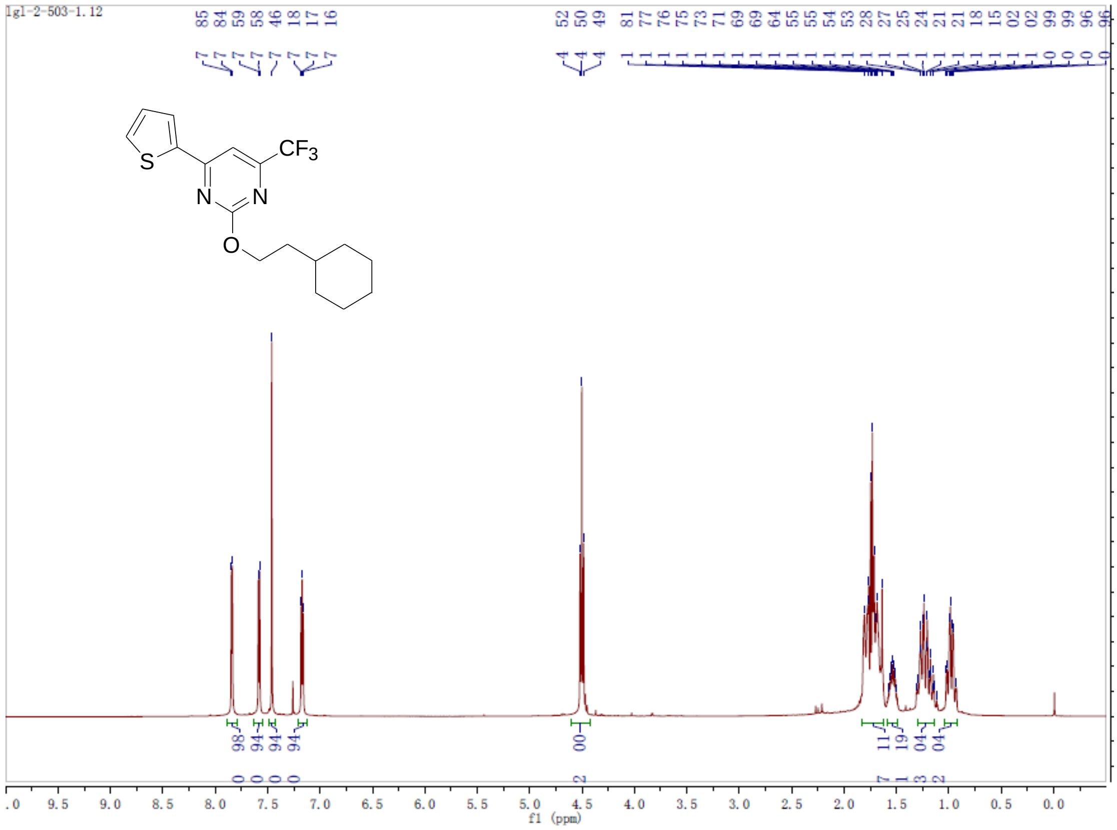

Supplementary Figure 70.^{1}H NMR spectra ($CDCl_3$, 400 MHz) of compound 4u

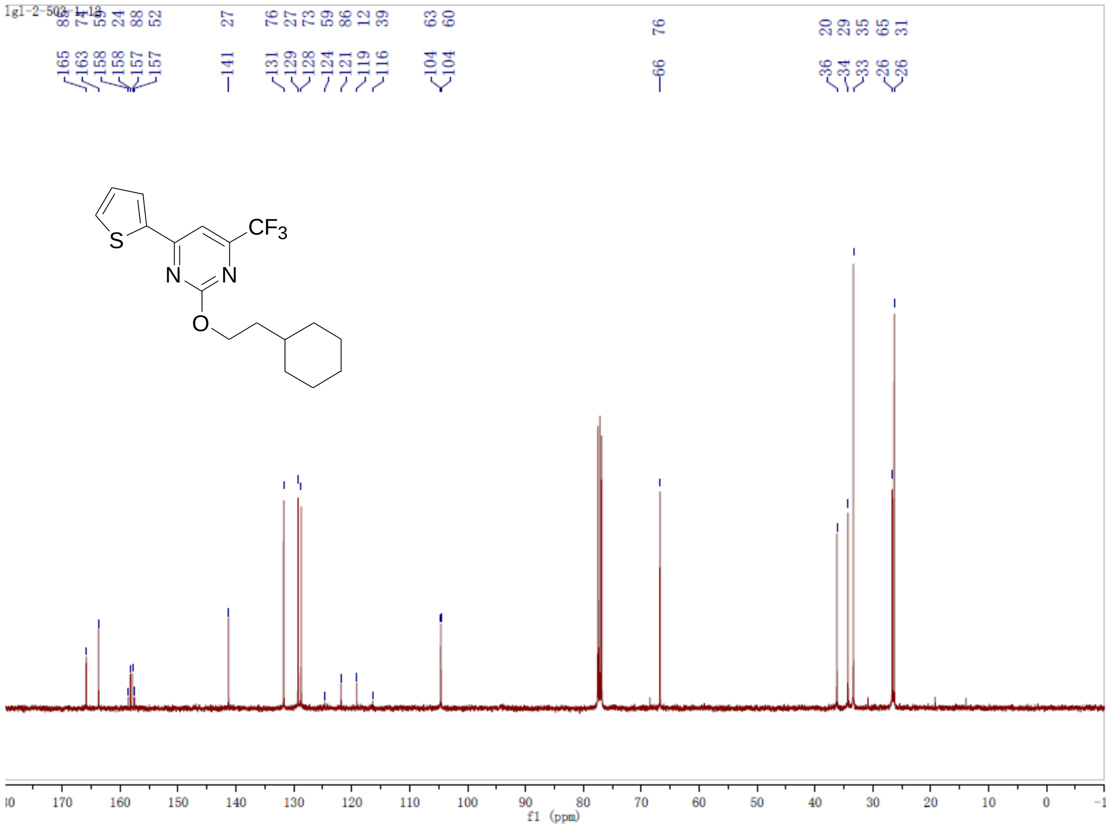

Supplementary Figure 71.^{13}C NMR spectra ($CDCl_3$, 100 MHz) of compound 4u

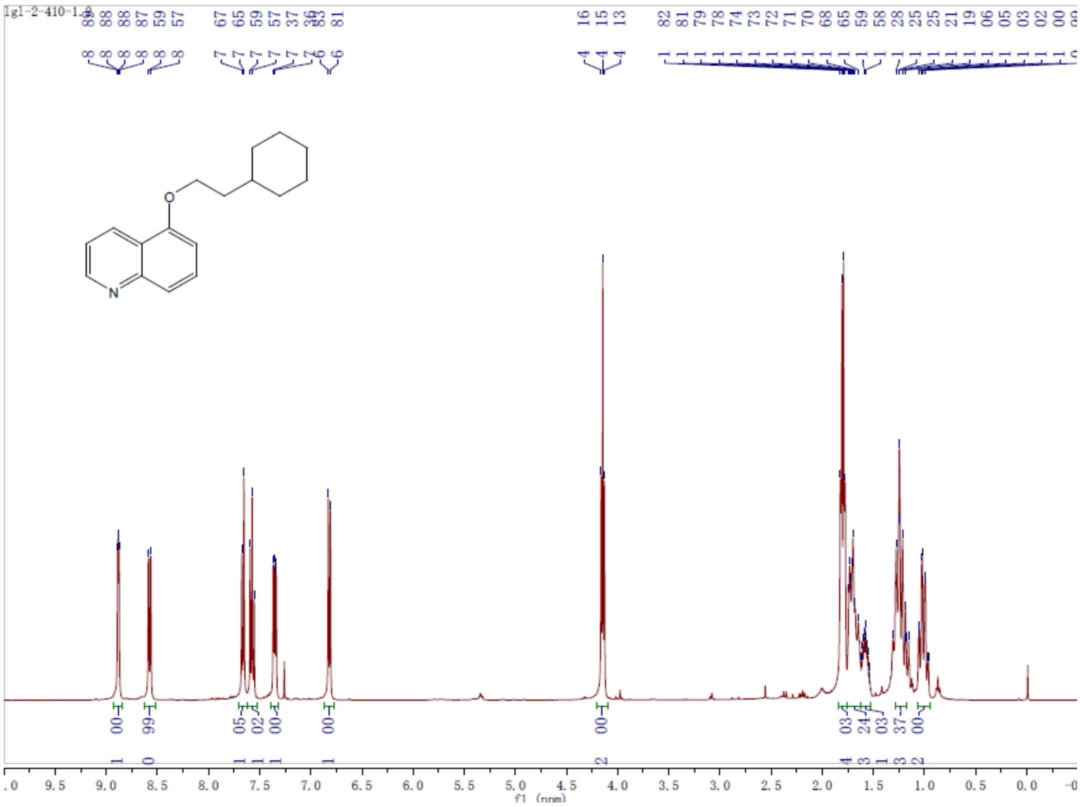

Supplementary Figure 72.^{1}H NMR spectra ($CDCl_3$, 400 MHz) of compound 4p (Gram scale)

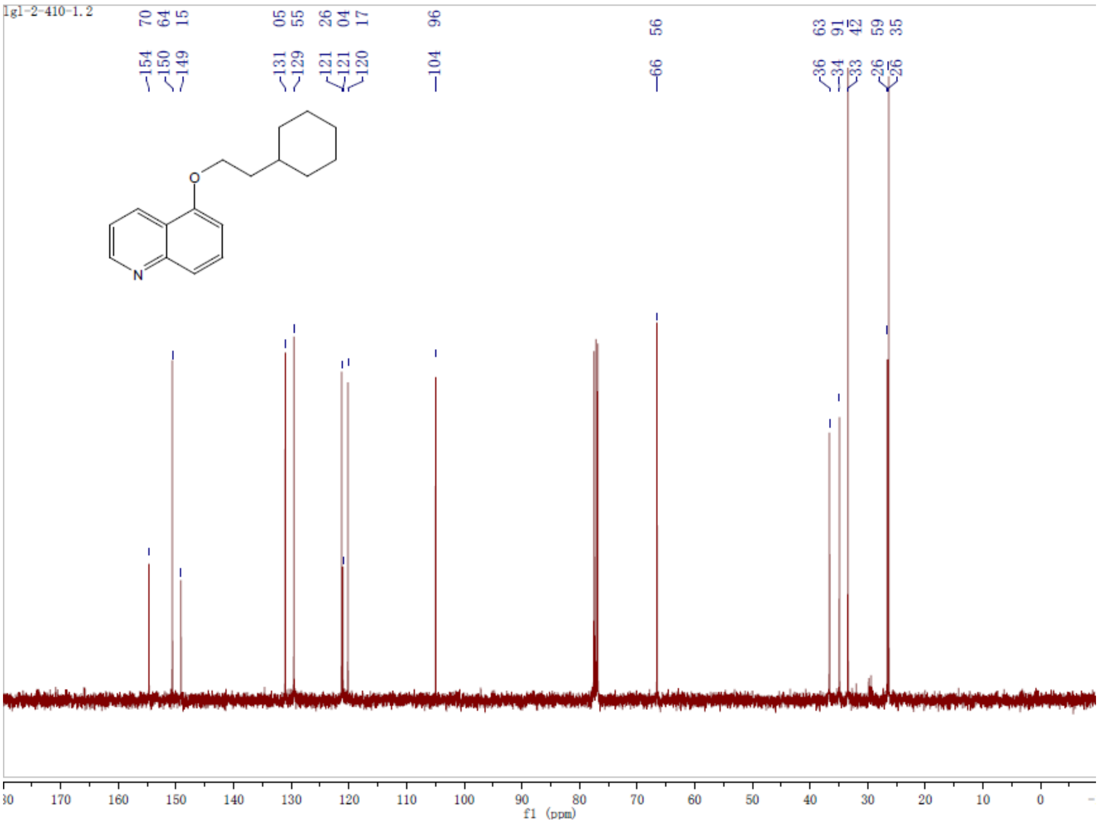

Supplementary Figure 73.^{13}C NMR spectra ($CDCl_3$, 100 MHz) of compound 4p (Gram scale)

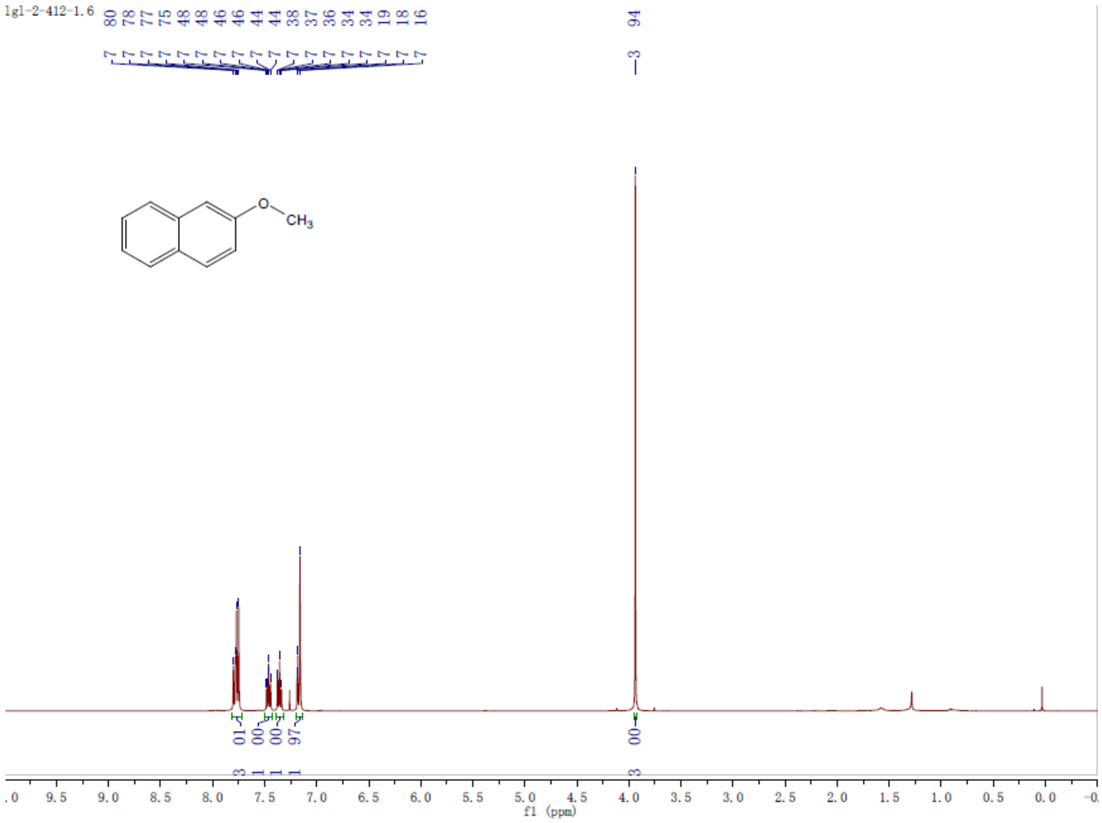

Supplementary Figure 74.^{1}H NMR spectra ($CDCl_3$, 400 MHz) of compound 3a (from 5b)

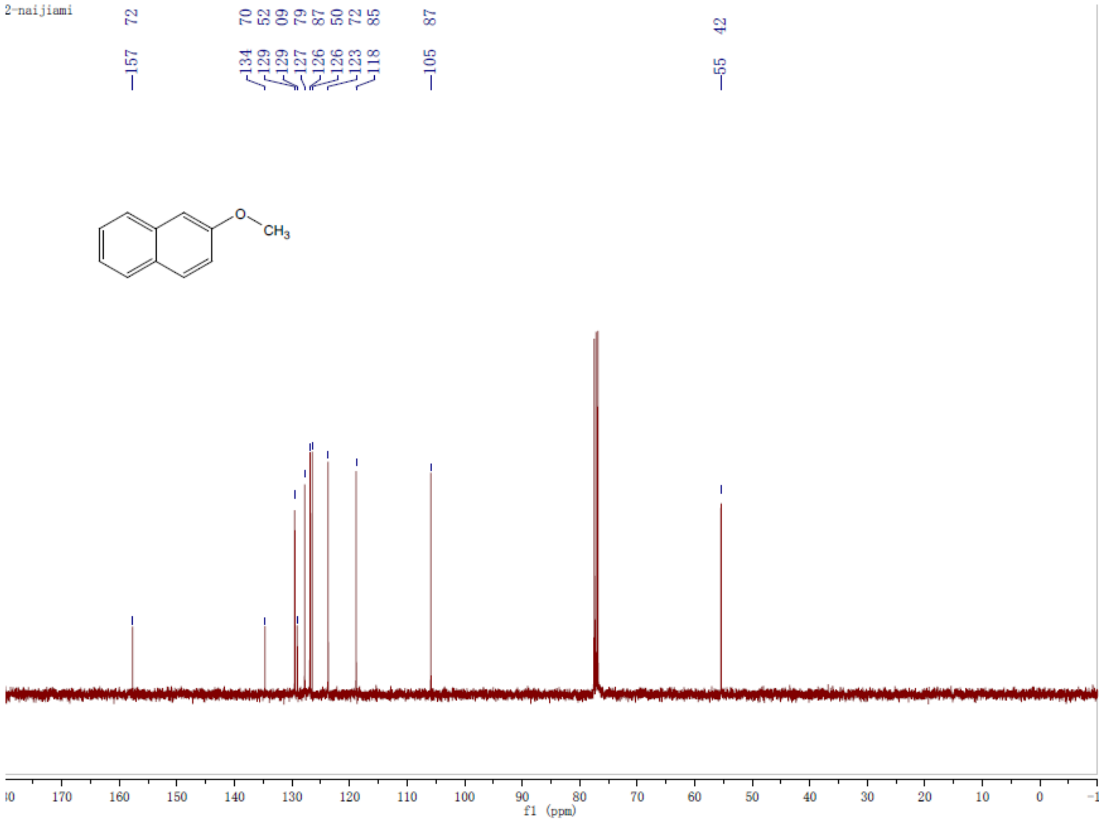

Supplementary Figure 75.^{13}C NMR spectra ($CDCl_3$, 100 MHz) of compound 3a (from 5b)

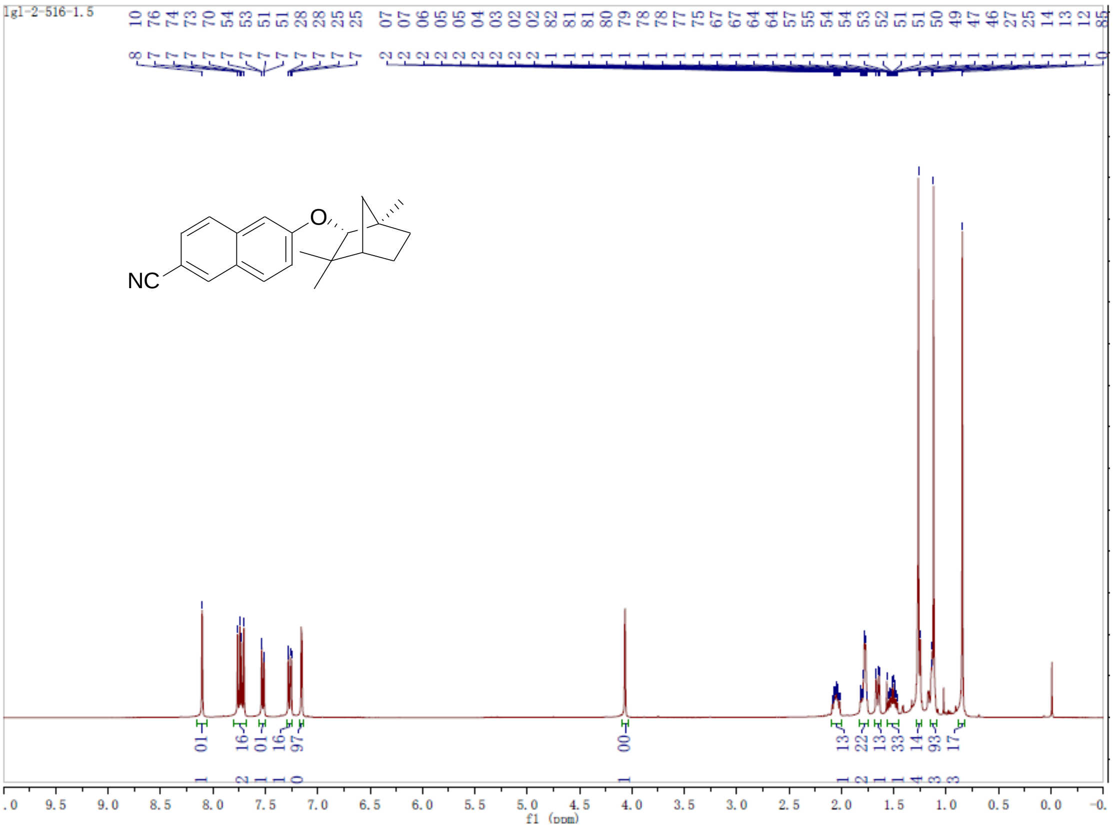

Supplementary Figure 76.^{1}H NMR spectra ($CDCl_3$, 400 MHz) of compound 6b

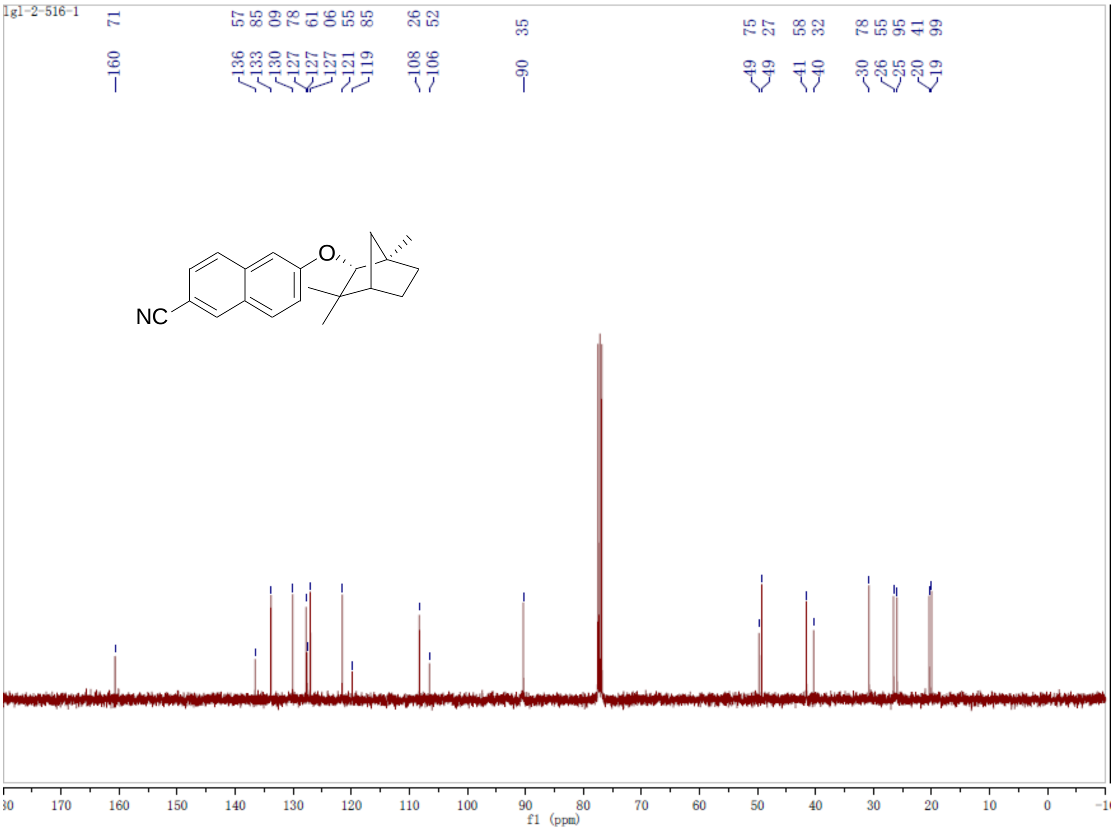

Supplementary Figure 77.^{13}C NMR spectra ($CDCl_3$, 100 MHz) of compound 6b

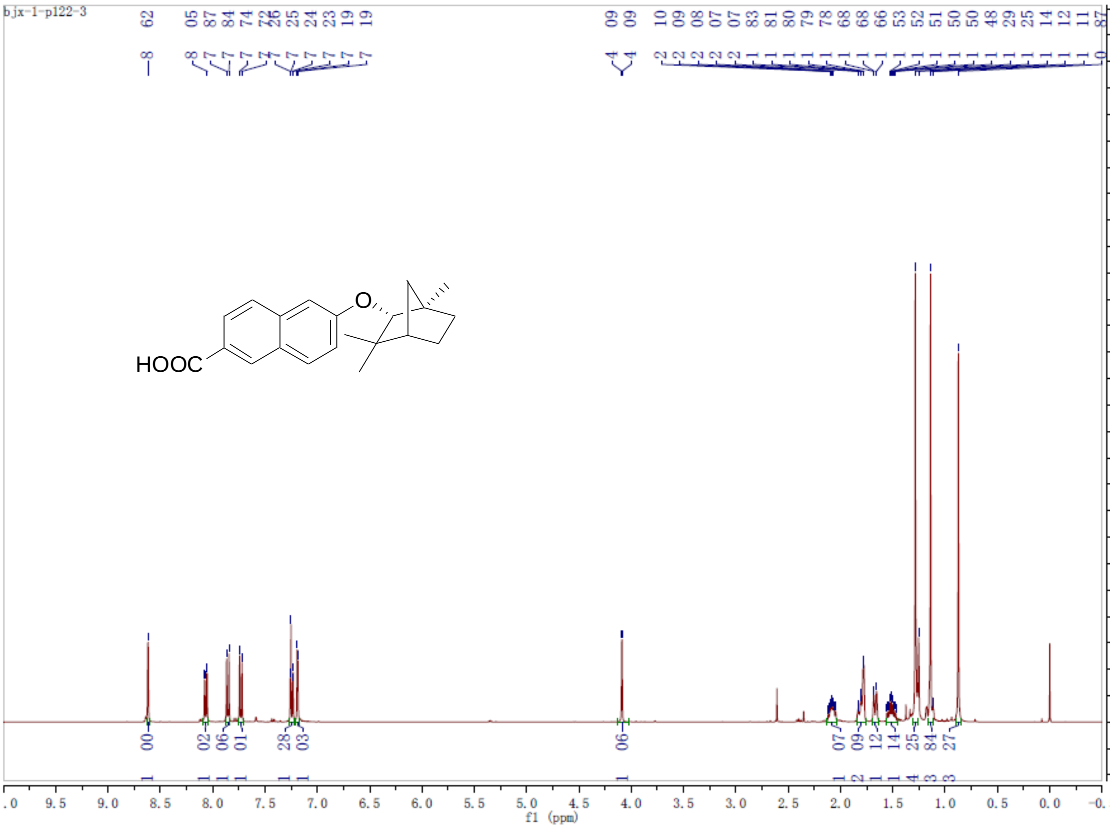

Supplementary Figure 78.^{1}H NMR spectra ($CDCl_3$, 400 MHz) of compound 6c

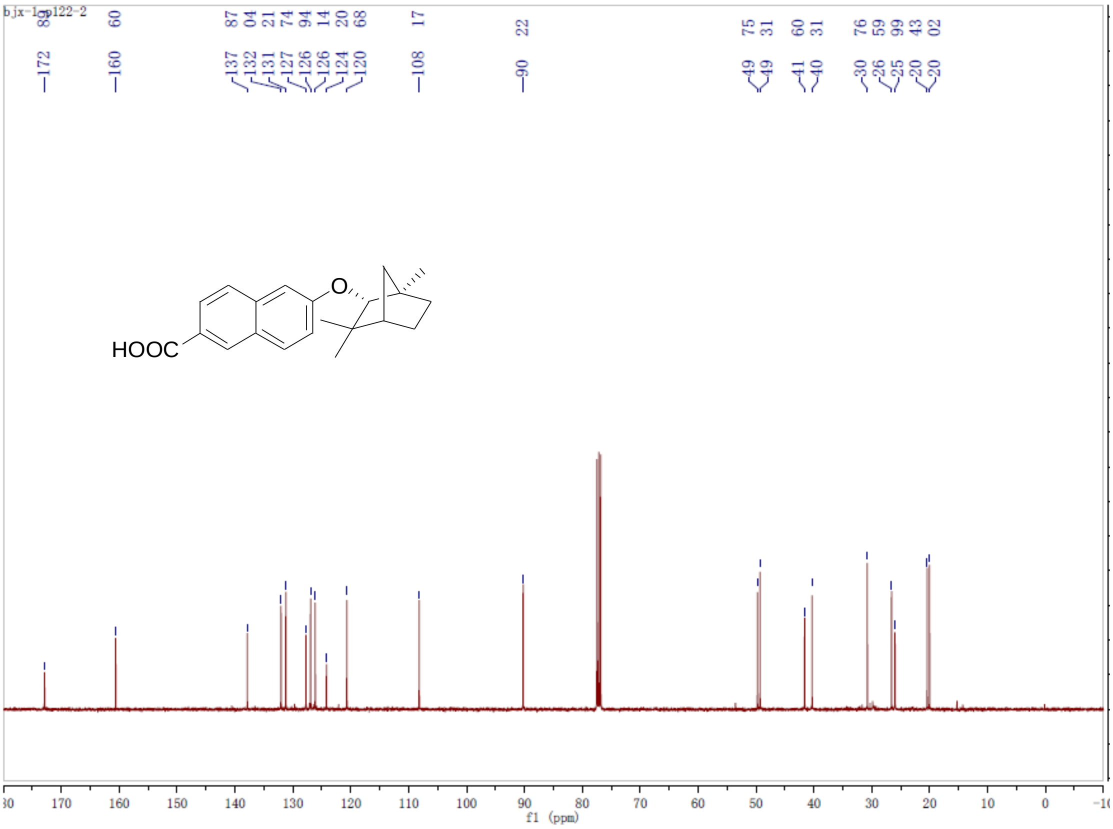

Supplementary Figure 79.^{13}C NMR spectra ($CDCl_3$, 100 MHz) of compound 6c

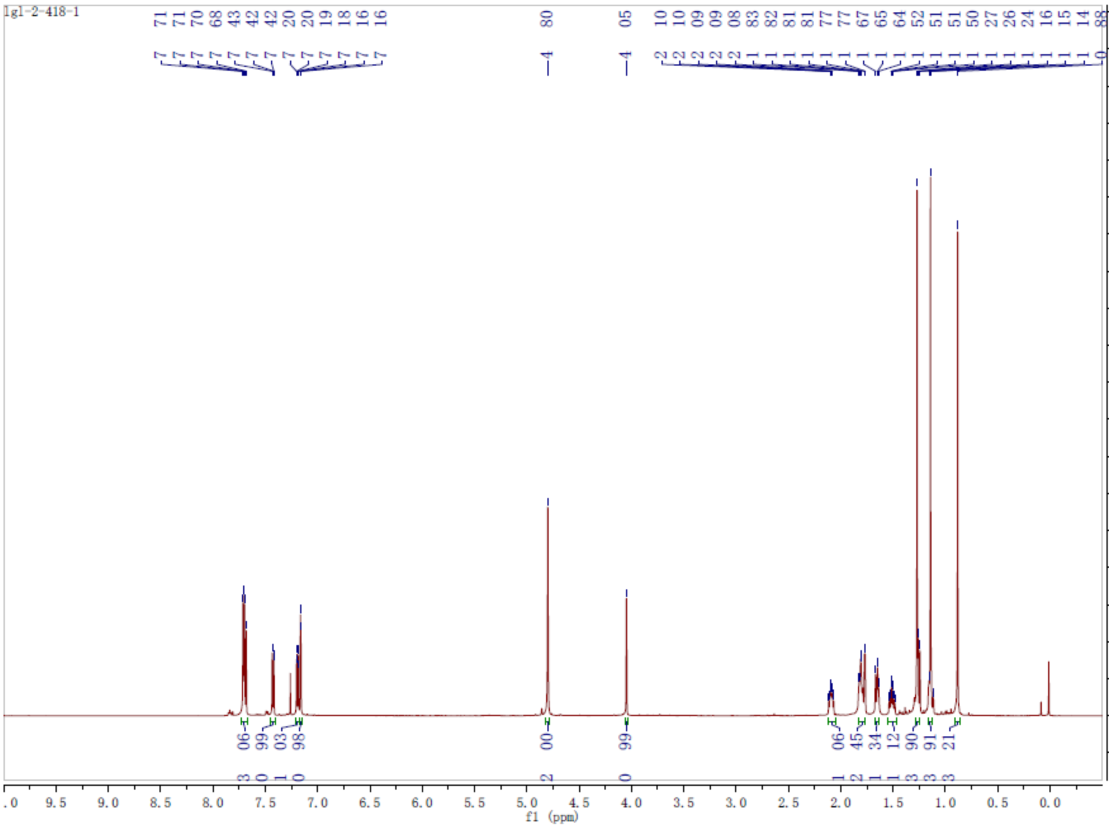

Supplementary Figure 80. ^{1}H **NMR spectra ($CDCl_3$, 400 MHz) of compound 6d**

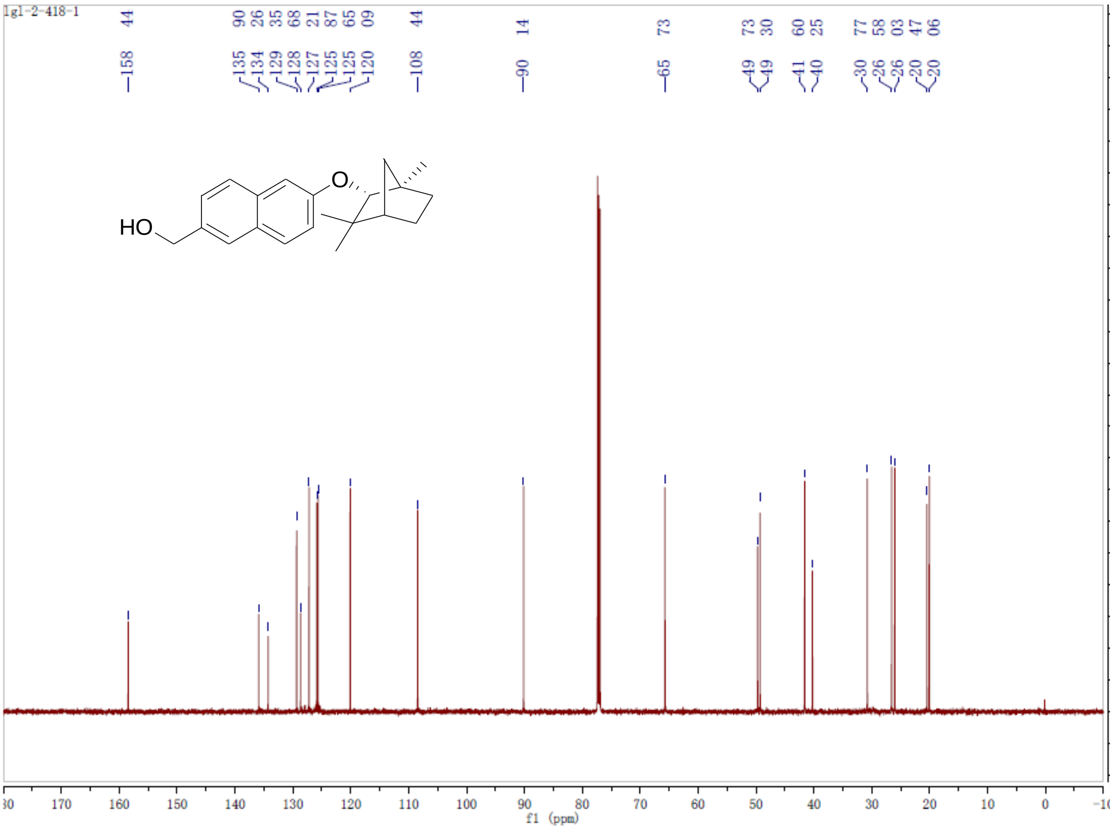

Supplementary Figure 81. ^{13}C **NMR spectra ($CDCl_3$, 100 MHz) of compound 6d**

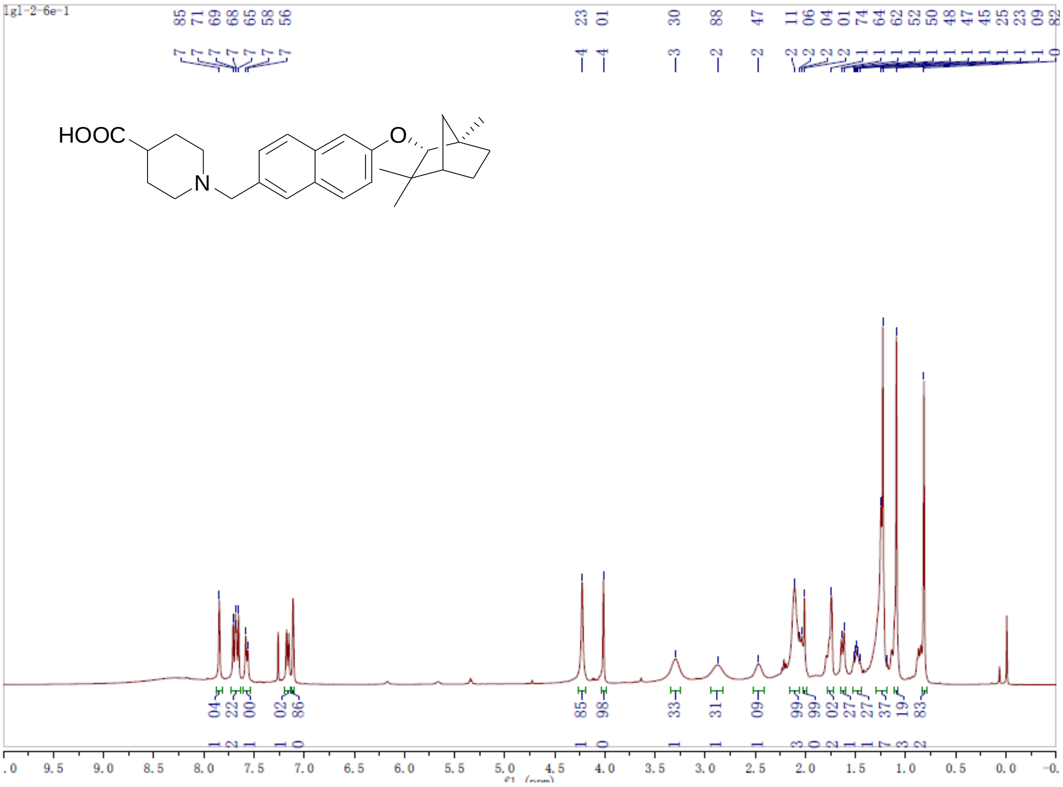

Supplementary Figure 82.^{1}H NMR spectra ($CDCl_3$, 400 MHz) of compound 6e

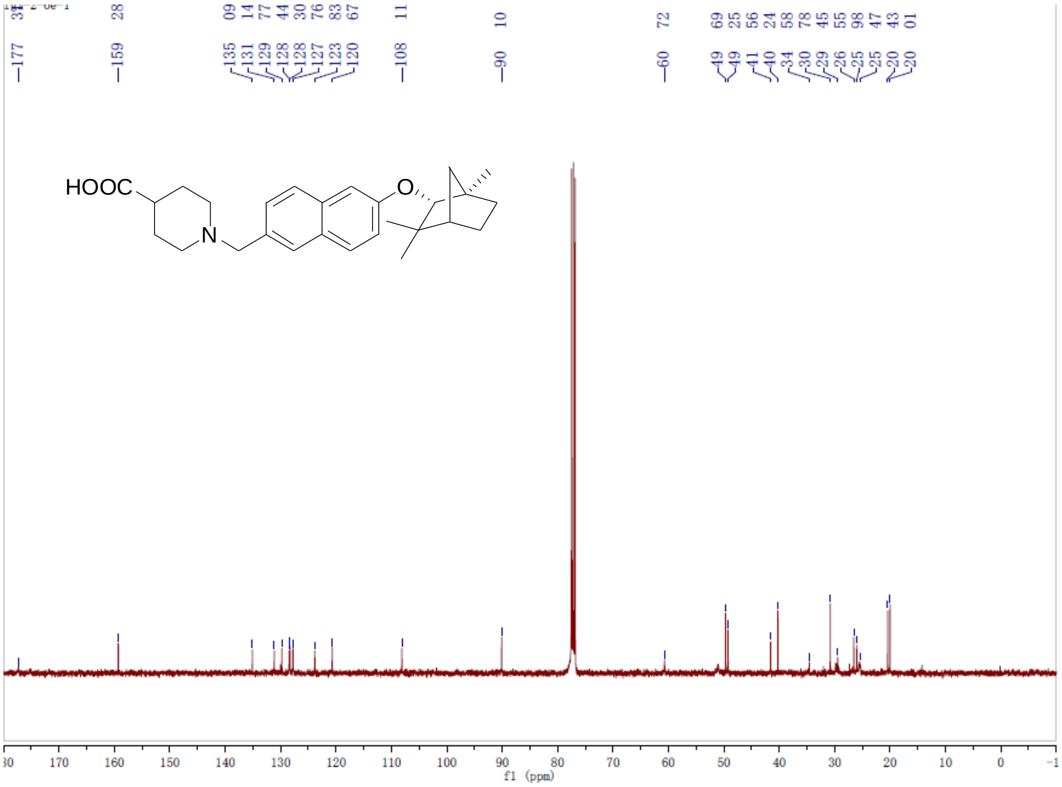

Supplementary Figure 83.^{13}C NMR spectra ($CDCl_3$, 100 MHz) of compound 6e

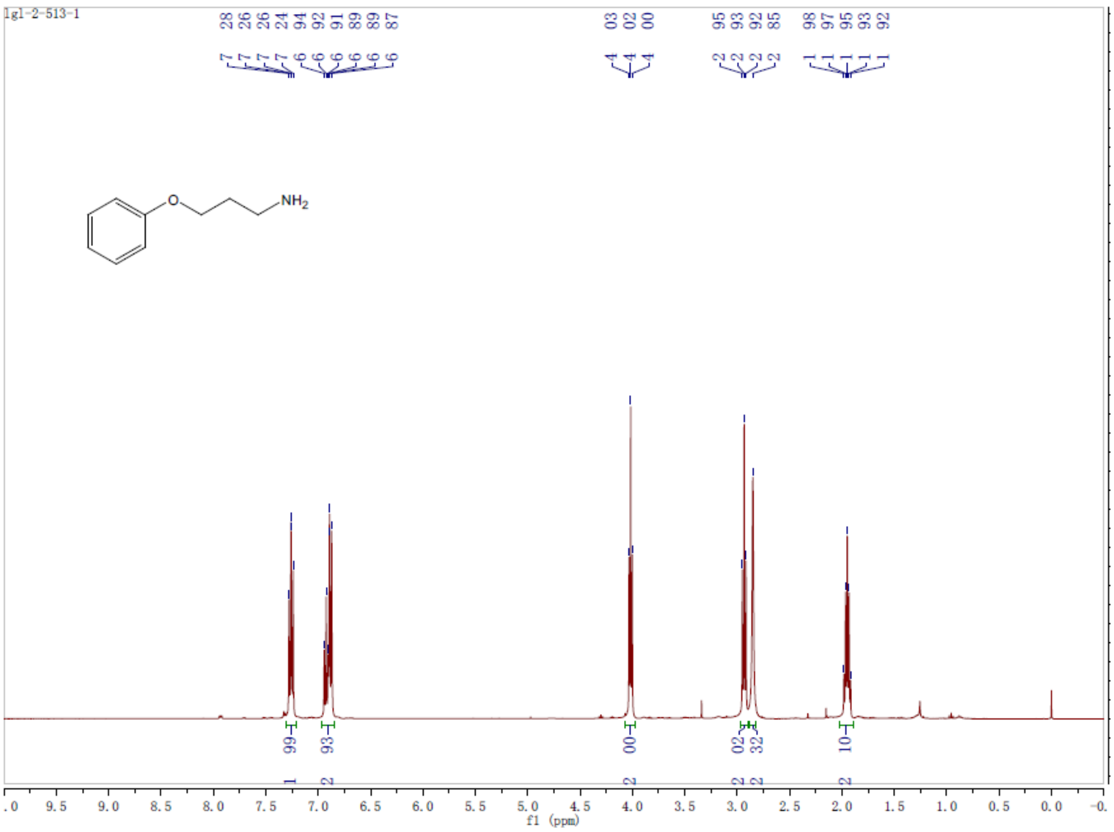

Supplementary Figure 84.^{1}H NMR spectra ($CDCl_3$, 400 MHz) of compound 7a

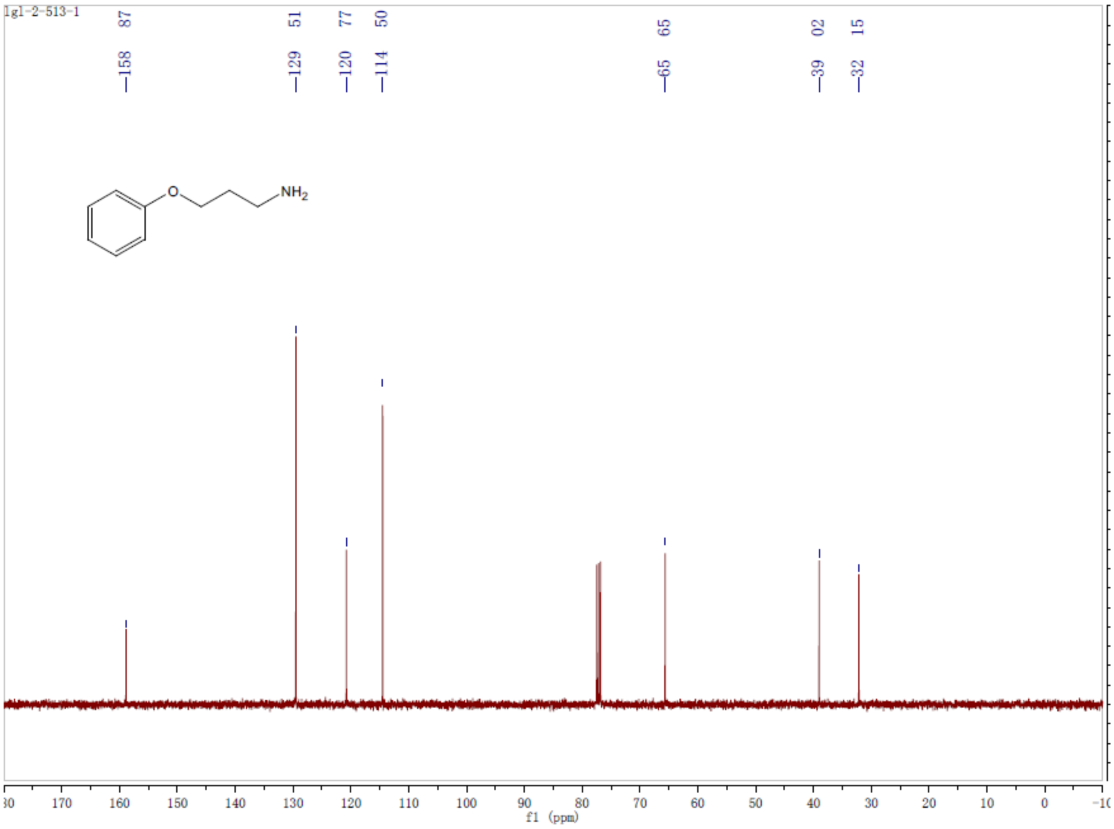

Supplementary Figure 85.^{13}C NMR spectra ($CDCl_3$, 100 MHz) of compound 7a

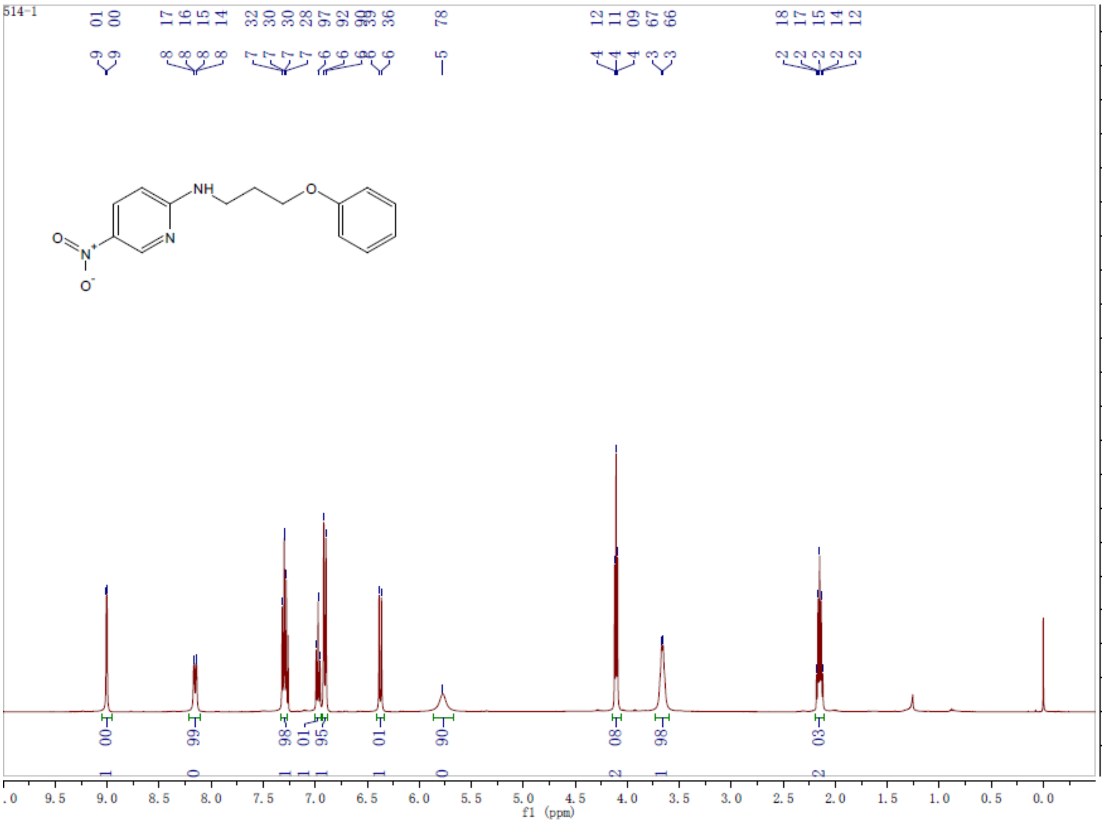

Supplementary Figure 86.^{1}**H NMR spectra (CDCl$_3$, 400 MHz) of compound 7b**

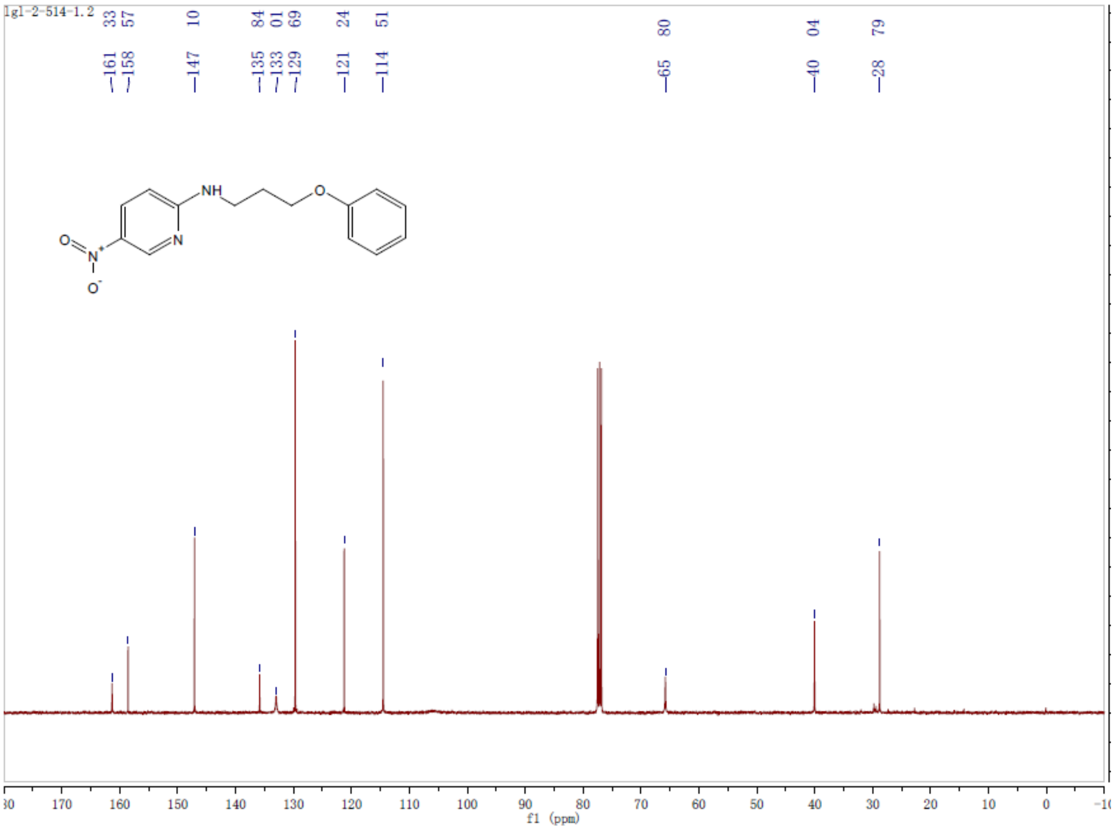

Supplementary Figure 87.^{13}**C NMR spectra (CDCl$_3$, 100 MHz) of compound 7b**

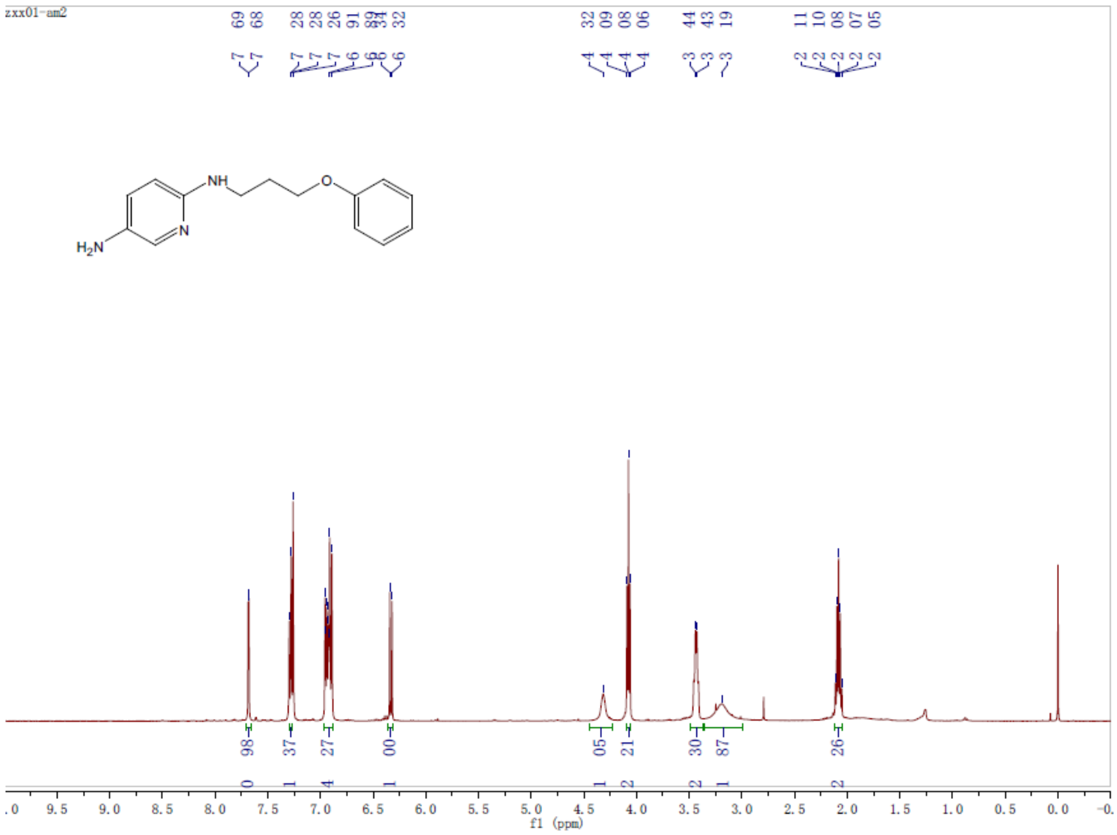

Supplementary Figure 88. ^{1}H **NMR spectra ($CDCl_3$, 400 MHz) of compound 7c**

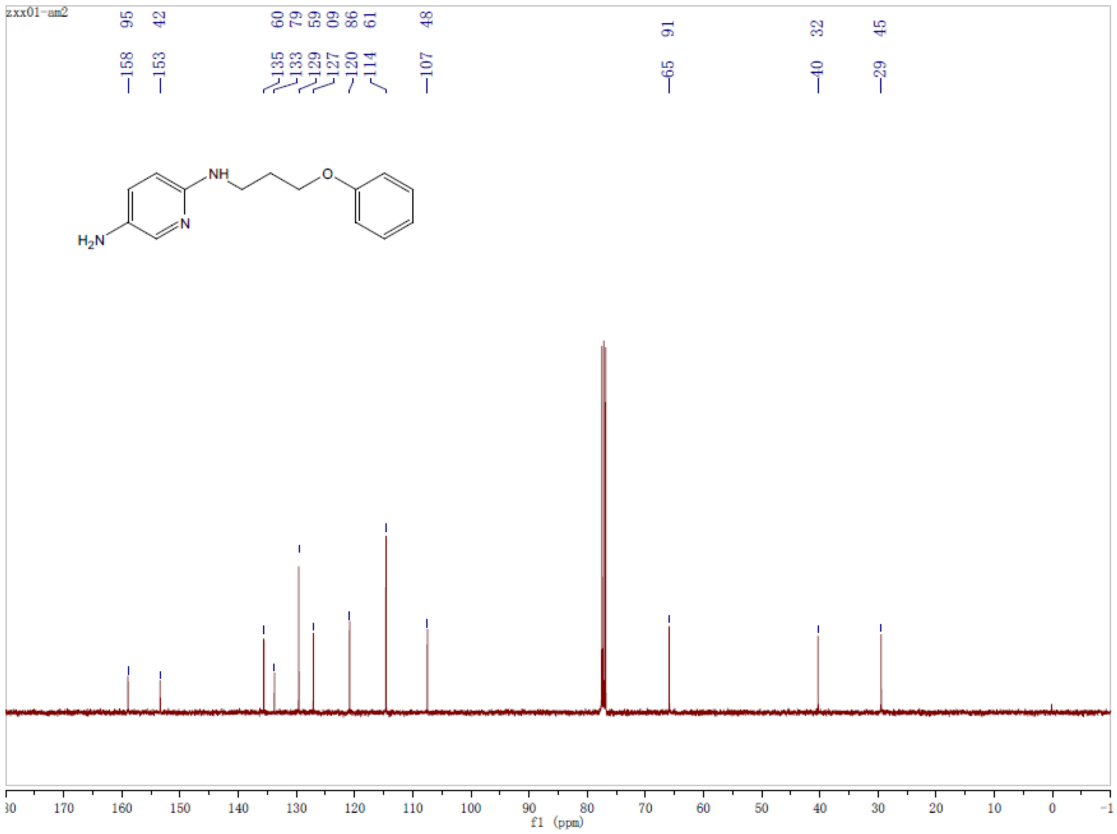

Supplementary Figure 89. ^{13}C **NMR spectra ($CDCl_3$, 100 MHz) of compound 7c**

Supplementary References.

1. Lii, P. J. & Shulgin, A. T. Metallation - Sulfidation: A Convenient Method for the Synthesis of Aryl Alkyl Sulfides and of Unsymmetrical Diaryl Sulfides. *Synth. Comm.* **11**, 957-968 (2006).
2. Renand, P., Bourquard, T., Carrupt, P.-A. & Gerster, M. Origin of Stereoselectivity in (Ethoxycarbonyl)-, Cyano-, and Phenyl-Substituted (Arylsulfinyl)methyl Radicals. *Helv. Chim. Acta* **81**, 1048 (1998).
3. Kumar, B. S., Ravi, K., Verma, A. K., Fatima, K., Hasanain, M., Singh, A., Negi, A. S. Synthesis of Pharmacologically Important Naphthoquinones and Anticancer Activity of 2-Benzyllawsone through DNA Topoisomerase-II Inhibition. *Bioorg. Med. Chem.* **25**, 1364-1373 (2017).
4. Cazorla, C., Pfordt, É., Duclos, M.-C., Métay, E. & Lemaire, M. O-Alkylation of Phenol Derivatives via a Nucleophilic Substitution. *Green Chem.* **13**, 2482 (2011).
5. Barbasiewicz, M., Szadkowska, A., Makal, A., Jarzembska, K., Wozniak, K. & Grela, K. Is the Hoveyda-Grubbs Complex a Vinylogous Fischer-Type Carbene? Aromaticity-Controlled Activity of Ruthenium Metathesis Catalysts. *Chem. Eur. J.* **14**, 9330-9337 (2008).
6. Ghiaci, M., Sedaghat, M. E., Kalbasi, R. J. & Abbaspur, A. Applications of Surfactant-Modified Clays to Synthetic Organic Chemistry. *Tetrahedron* **61**, 5529-5534 (2005).
7. Kozacik, A. P. & Reid, E. E. Lengthening Carbon Chains by Three Units: Assay of Primary Bromides from the Addition of Hydrogen Bromide. *J. Am. Chem. Soc.* **60**, 2463 (1938).
8. Adamson, D. W. & Kenner, J. The Preparation of Diazomethane and its Homologues. *J. Chem. Soc.* 286 (1935).
9. Jadhav, V. H., Jeong, H.-J., Lim, S. T., Sohn, M.-H., Song, C. E. & Kim, D. W. Polymer-Supported Oligoethylene Glycols as Heterogeneous Multifunctional Catalysts for Nucleophilic Substitution. *Tetrahedron* **69**, 3577-3583 (2013).
10. Behnke, N. E., Kielawa, R., Kwon, D. H., Ess, D. H. & Kurti, L. Direct Primary Amination of Alkylmetals with NH-Oxaziridine. *Org. Lett.* **20**, 8064-8068 (2018).
11. Gaussian 09, Revision D.01, M. J. Frisch, G. W. Trucks, H. B. Schlegel, G. E. Scuseria, M. A. Robb, J. R. Cheeseman, G. Scalmani, V. Barone, B. Mennucci, G. A. Petersson, H. Nakatsuji, M. Caricato, X. Li, H. P. Hratchian, A. F. Izmaylov, J. Bloino, G. Zheng, J. L. Sonnenberg, M. Hada, M. Ehara, K. Toyota, R. Fukuda, J. Hasegawa, M. Ishida, T. Nakajima, Y. Honda, O. Kitao, H. Nakai, T. Vreven, Jr. J. A. Montgomery, J. E. Peralta, F. Ogliaro, M. Bearpark, J. J. Heyd, E. Brothers, K. N. Kudin, V. N. Staroverov, T. Keith, R. Kobayashi, J. Normand, K. Raghavachari, A. Rendell, J. C. Burant, S. S. Iyengar, J. Tomasi, M. Cossi, N. Rega, J. M. Millam, M. Klene, J. E. Knox, J. B. Cross, V. Bakken, C. Adamo, J. Jaramillo, R. Gomperts, R. E. Stratmann, O. Yazyev, A. J. Austin, R. Cammi, C. Pomelli, J. W. Ochterski, R. L. Martin, K. Morokuma, V. G. Zakrzewski, G. A. Voth, P. Salvador, J. J. Dannenberg, S. Dapprich, A. D. Daniels, O. Farkas, J. B. Foresman, J. V. Ortiz, J. Cioslowski, and Fox, D. J. Gaussian, Inc., Wallingford CT, 2013.
