# Supplementary material for: Transition-metal-free formal cross-coupling of aryl methyl sulfoxides and alcohols via nucleophilic activation of C-S bond
Source: Nat Commun. 2020 Jun 8;11:2890. doi: 10.1038/s41467-020-16713-8 (PMC7280189; doi:10.1038/s41467-020-16713-8)
Supplement: Supplementary file 3 — Description of Additional Supplementary Files [file 41467_2020_16713_MOESM3_ESM.pdf]

Description of Additional Supplementary Files

File Name: Supplementary Data 1

Description: Coordinates and thermochemical data for computed intermediates and transition states of three possible reaction mechanisms in Fig. 5 are summarized in Supplementary Data 1.
