# Supplementary material for: Transition-metal-free formal cross-coupling of aryl methyl sulfoxides and alcohols via nucleophilic activation of C-S bond
Source: Nat Commun. 2020 Jun 8;11:2890. doi: 10.1038/s41467-020-16713-8 (PMC7280189; doi:10.1038/s41467-020-16713-8)
Supplement: Supplementary file 4 — Supplementary Data 1 [file 41467_2020_16713_MOESM4_ESM.zip › 236624_2_data_set_4600485_q9wtn7 (1).docx]

**Coordinates and thermochemical data for computed intermediates and transition states**

***Mechanism C:***

2-(methylsulfinyl)naphthalene (Starting Material):

Zero-point correction= 0.179614 (Hartree/Particle)

Thermal correction to Energy= 0.190867

Thermal correction to Enthalpy= 0.191812

Thermal correction to Gibbs Free Energy= 0.141751

Sum of electronic and zero-point Energies= -898.409376

Sum of electronic and thermal Energies= -898.398123

Sum of electronic and thermal Enthalpies= -898.397178

Sum of electronic and thermal Free Energies= -898.447239

Electronic Energy = -898.35102

C,0,-3.6074525215,-1.253495161,0.2550141938

C,0,-2.2476364982,-1.1157373816,0.4346013237

C,0,-1.5917191309,0.1074483039,0.1251190747

C,0,-2.3714168052,1.2008968067,-0.3801113055

C,0,-3.7718520881,1.0258649009,-0.5580302036

C,0,-4.3767943243,-0.172776027,-0.2468559006

H,0,0.4016005207,-0.5569860672,0.65860739

H,0,-4.0960322023,-2.1938150466,0.4965123798

H,0,-1.6563556875,-1.9447869043,0.8169126465

C,0,-0.1885398726,0.2792140828,0.2898409582

C,0,-1.7154493799,2.4224466759,-0.6953765406

H,0,-4.3599815667,1.8553002695,-0.9447842187

H,0,-5.447808663,-0.2944147686,-0.38603611

C,0,-0.3588427274,2.5501537028,-0.4957617229

C,0,0.4249946274,1.4737108119,-0.0176265924

H,0,-2.3011463154,3.2470356009,-1.0994907063

H,0,1.4986440263,1.609144326,0.0800893549

S,0,0.4822307005,4.1146548405,-0.9297083985

O,0,1.9725694285,3.8230069957,-0.9850680405

C,0,0.1931001414,4.9714946525,0.6742679419

H,0,-0.8814617715,5.0961042935,0.8376195438

H,0,0.6456516482,4.3819130281,1.4762585907

H,0,0.6819292414,5.9469899745,0.6016296316

Potassium methoxide:

Zero-point correction= 0.039095 (Hartree/Particle)

Thermal correction to Energy= 0.043750

Thermal correction to Enthalpy= 0.044694

Thermal correction to Gibbs Free Energy= 0.011832

Sum of electronic and zero-point Energies= -715.007866

Sum of electronic and thermal Energies= -715.003211

Sum of electronic and thermal Enthalpies= -715.002267

Sum of electronic and thermal Free Energies= -715.035129

Electronic Energy = -714.9726

O,0,0.8122484304,0.0010335842,-0.0008016801

C,0,2.189316493,-0.0012477834,-0.0000963245

H,0,2.6265007903,-0.5155612949,-0.8870050031

H,0,2.6285384342,1.0231141207,-0.0011373146

H,0,2.6256985978,-0.5134112937,0.8884772669

K,0,-1.4679217456,0.0006316671,0.0005460554

Sodium methoxide:

Zero-point correction= 0.039495 (Hartree/Particle)

Thermal correction to Energy= 0.044139

Thermal correction to Enthalpy= 0.045083

Thermal correction to Gibbs Free Energy= 0.012741

Sum of electronic and zero-point Energies= -277.394927

Sum of electronic and thermal Energies= -277.390283

Sum of electronic and thermal Enthalpies= -277.389339

Sum of electronic and thermal Free Energies= -277.421681

Electronic Energy = -277.3477

O,0,-0.8079053359,0.0012895045,-0.0004294415

C,0,-2.185091388,-0.0006347567,0.0002126963

H,0,-2.6176992376,0.3985515355,0.9442090715

H,0,-2.6188068175,0.6166102033,-0.8174224854

H,0,-2.6164057904,-1.0183747521,-0.1254806018

Na,0,1.1487060593,-0.0000561545,-0.0000261791

Lithium methoxide:

Zero-point correction= 0.041214 (Hartree/Particle)

Thermal correction to Energy= 0.045418

Thermal correction to Enthalpy= 0.046362

Thermal correction to Gibbs Free Energy= 0.016601

Sum of electronic and zero-point Energies= -122.644422

Sum of electronic and thermal Energies= -122.640219

Sum of electronic and thermal Enthalpies= -122.639274

Electronic Energy = -122.6299

Sum of electronic and thermal Free Energies= -122.669035

O,0,0.2358219817,0.0000511014,0.000025544

C,0,1.6164580928,0.000574636,0.0002904249

H,0,2.0358485886,-0.4862453841,0.902719325

H,0,2.0361989641,-0.5372510477,-0.8725331039

H,0,2.035389707,1.025703123,-0.0290814397

Li,0,-1.3653896543,-0.0005712788,-0.0003105303

2-methoxynaphthalene (Product):

Zero-point correction= 0.179996 (Hartree/Particle)

Thermal correction to Energy= 0.189488

Thermal correction to Enthalpy= 0.190433

Thermal correction to Gibbs Free Energy= 0.145131

Sum of electronic and zero-point Energies= -500.252096

Sum of electronic and thermal Energies= -500.242603

Sum of electronic and thermal Enthalpies= -500.241659

Sum of electronic and thermal Free Energies= -500.286960

Electronic Energy = -500.2288

C,0,3.288554428,0.7649525007,-0.8281339055

C,0,2.222021389,1.3628575988,-0.193555064

C,0,0.904427768,0.8448943188,-0.3355665648

C,0,0.6928079955,-0.3163871187,-1.1528111052

C,0,1.8182821209,-0.9101766084,-1.7951348557

C,0,3.081220311,-0.383783611,-1.6369677709

H,0,-0.0711117907,2.3116302793,0.9227993203

H,0,4.2909213287,1.1689581189,-0.7126852408

H,0,2.3735338298,2.2433397957,0.4277279166

C,0,-0.218838103,1.4312991199,0.3008156351

C,0,-0.6166257528,-0.8342815532,-1.2962854715

H,0,1.6627987229,-1.7901643706,-2.4154544441

H,0,3.928814668,-0.8490599221,-2.1340953275

C,0,-1.6910368139,-0.2367069441,-0.6606189659

C,0,-1.4891589673,0.9124467479,0.1495052125

H,0,-0.792127674,-1.7127021003,-1.9114774881

H,0,-2.3233504623,1.3887443411,0.6522923072

O,0,-2.9160258848,-0.8144402541,-0.8633274654

C,0,-4.0595462068,-0.2471054259,-0.2385842133

H,0,-4.9017112451,-0.8697262401,-0.5459766598

H,0,-3.9727277069,-0.2691833648,0.8558138062

H,0,-4.2304611742,0.7846436522,-0.5732000354

KOSMe:

Zero-point correction= 0.040577 (Hartree/Particle)

Thermal correction to Energy= 0.046726

Thermal correction to Enthalpy= 0.047670

Thermal correction to Gibbs Free Energy= 0.009156

Sum of electronic and zero-point Energies= -1113.201624

Sum of electronic and thermal Energies= -1113.195475

Sum of electronic and thermal Enthalpies= -1113.194531

Sum of electronic and thermal Free Energies= -1113.233045

Electronic Energy = -1113.13

S,0,0.8796206712,-0.705072773,-0.0010495497

C,0,1.9051624208,0.8049494427,-0.0000517564

H,0,2.9563777,0.4921034808,-0.0002615683

H,0,1.7114956507,1.4094784673,-0.8940363903

H,0,1.7115112762,1.4083719677,0.8946839017

O,0,-0.6410429287,-0.0944777955,-0.0000915729

K,0,-2.8815748903,0.4197089,0.0002454158

NaOSMe:

Zero-point correction= 0.040997 (Hartree/Particle)

Thermal correction to Energy= 0.046243

Thermal correction to Enthalpy= 0.047187

Thermal correction to Gibbs Free Energy= 0.011470

Sum of electronic and zero-point Energies= -675.581931

Sum of electronic and thermal Energies= -675.576685

Sum of electronic and thermal Enthalpies= -675.575741

Sum of electronic and thermal Free Energies= -675.611458

Electronic Energy = -675.5061

S,0,-0.8986996296,-0.6713294002,0.0000042998

C,0,-1.978554174,0.7972762753,0.0000852008

H,0,-3.017624393,0.443115267,0.0007805044

H,0,-1.8074436343,1.4087246049,0.8945865856

H,0,-1.8084170145,1.4080790102,-0.8950390497

O,0,0.5911047463,0.0118611441,-0.0006587539

Na,0,2.5587030991,0.2327580986,0.000324213

LiOSMe:

Zero-point correction= 0.042378 (Hartree/Particle)

Thermal correction to Energy= 0.048042

Thermal correction to Enthalpy= 0.048987

Thermal correction to Gibbs Free Energy= 0.013507

Sum of electronic and zero-point Energies= -520.826445

Sum of electronic and thermal Energies= -520.820781

Sum of electronic and thermal Enthalpies= -520.819837

Sum of electronic and thermal Free Energies= -520.855316

Electronic Energy = -520.7838

S,0,0.8719155622,-0.6785698659,-0.0003537235

C,0,1.9307536663,0.8016069504,-0.0001161878

H,0,2.9717486392,0.45477739,-0.0002897918

H,0,1.7556509686,1.4092539289,-0.8955054714

H,0,1.7558168965,1.4088597426,0.8955730864

O,0,-0.6238616961,-0.0053195585,-0.0000162981

Li,0,-2.2251789367,0.2578194425,0.0005440462

**INT1** with Potassium:

Zero-point correction= 0.220107 (Hartree/Particle)

Thermal correction to Energy= 0.237297

Thermal correction to Enthalpy= 0.238241

Thermal correction to Gibbs Free Energy= 0.173566

Sum of electronic and zero-point Energies= -1613.403505

Sum of electronic and thermal Energies= -1613.386315

Sum of electronic and thermal Enthalpies= -1613.385371

Sum of electronic and thermal Free Energies= -1613.450046

Electronic Energy = -1613.3184

C,0,4.2366711186,-0.3076296552,0.7358044707

C,0,3.2461454086,0.5913029292,1.0773548783

C,0,1.9005034481,0.3839760409,0.6730540191

C,0,1.5716445939,-0.7800983389,-0.1017820751

C,0,2.6179327785,-1.6887732044,-0.4357973643

C,0,3.9155395509,-1.4581144947,-0.0284154819

H,0,1.0816602677,2.1651902105,1.6077260538

H,0,5.2612199572,-0.1413795984,1.0578116075

H,0,3.4838563117,1.4703755015,1.6733780488

C,0,0.8488280718,1.2875670315,1.0073316594

C,0,0.2292755025,-0.9783091592,-0.5293577669

H,0,2.3731025109,-2.5803868352,-1.0095112483

H,0,4.6984282184,-2.1672238823,-0.2859248203

C,0,-0.7802591147,-0.099245877,-0.1620289797

C,0,-0.4466408557,1.0615226454,0.6002572856

H,0,0.0031452911,-1.8610185828,-1.1212362953

H,0,-1.2369411409,1.751803734,0.8821887486

S,0,-2.4116868723,-0.0758793269,-0.9418355987

O,0,-2.0435466684,0.5668584386,-2.3541132162

C,0,-2.6636840628,-1.8235981113,-1.4222611349

H,0,-1.9922435209,-2.0454660083,-2.2561743389

H,0,-2.524701155,-2.4642750181,-0.5527663298

H,0,-3.6976188671,-1.8786555562,-1.7733605091

O,0,-2.5296522089,-1.07649093,1.1299728583

C,0,-2.9824031891,-0.2766529034,2.1584315163

H,0,-3.5801381655,-0.8580358593,2.8890863044

H,0,-2.1622927018,0.1995167635,2.7451892936

H,0,-3.6410830572,0.5535612935,1.809409556

K,0,0.2694948703,1.436043213,-2.6422890809

**INT1** with Sodium:

Zero-point correction= 0.220736 (Hartree/Particle)

Thermal correction to Energy= 0.237670

Thermal correction to Enthalpy= 0.238614

Thermal correction to Gibbs Free Energy= 0.175176

Sum of electronic and zero-point Energies= -1175.788522

Sum of electronic and thermal Energies= -1175.771587

Sum of electronic and thermal Enthalpies= -1175.770643

Sum of electronic and thermal Free Energies= -1175.834082

Electronic Energy = -1175.69

C,0,-4.3596952072,0.5691675166,-0.0687931588

C,0,-3.4261285902,0.7494691399,0.9316052676

C,0,-2.0394799626,0.5859405247,0.6723409659

C,0,-1.6114197392,0.225433038,-0.6493434043

C,0,-2.5997939373,0.0480901098,-1.6593938141

C,0,-3.9393488061,0.2157253813,-1.3757010147

H,0,-1.358854886,1.0425555477,2.6825373394

H,0,-5.4175818317,0.7038143058,0.1399845685

H,0,-3.7418338328,1.0292636078,1.9346119514

C,0,-1.0451067425,0.7642243865,1.6784111341

C,0,-0.2218436056,0.0411072473,-0.9064243894

H,0,-2.2797964584,-0.2110622071,-2.6664742174

H,0,-4.6800112397,0.0836971017,-2.1603849481

C,0,0.7271610834,0.266623034,0.0826402988

C,0,0.2938678795,0.604613279,1.4014724539

H,0,0.0849224325,-0.2241819124,-1.9148527576

H,0,1.0371900724,0.7672681266,2.1765869545

S,0,2.4637822712,-0.2148193431,-0.0789405851

O,0,2.307748934,-1.8075947304,0.0882665752

C,0,2.7674942471,-0.123806071,-1.8817502887

H,0,2.2423909612,-0.9577013282,-2.3544528215

H,0,2.4794072489,0.8577709136,-2.2542330509

H,0,3.8446837733,-0.2700580368,-1.9970983922

O,0,2.3105149449,2.0056606232,-0.3969703407

C,0,2.6525023124,2.7788832524,0.6963643905

H,0,3.1614338198,3.7113934503,0.3836657557

H,0,1.7751358274,3.0949596349,1.3051808234

H,0,3.3510765939,2.2598539245,1.3925476853

Na,0,0.3003972372,-2.3343005867,0.5152215393

**INT1** with Lithium:

Zero-point correction= 0.222251 (Hartree/Particle)

Thermal correction to Energy= 0.238567

Thermal correction to Enthalpy= 0.239512

Thermal correction to Gibbs Free Energy= 0.177641

Sum of electronic and zero-point Energies= -1021.028901

Sum of electronic and thermal Energies= -1021.012584

Sum of electronic and thermal Enthalpies= -1021.011640

Sum of electronic and thermal Free Energies= -1021.073511

Electronic Energy = -1020.9645

C,0,-4.4775463683,0.4574626456,-0.1020268689

C,0,-3.547578445,0.8308705959,0.8472132547

C,0,-2.158118982,0.6598037494,0.6108336811

C,0,-1.7301472537,0.0853892648,-0.6298416457

C,0,-2.7102461039,-0.2839239792,-1.5906686467

C,0,-4.0539961109,-0.1026800692,-1.3319996138

H,0,-1.4884044412,1.491197106,2.499350093

H,0,-5.5381034072,0.596921148,0.0892964293

H,0,-3.868840735,1.2687801364,1.7896307975

C,0,-1.1666696676,1.0452620409,1.5607428173

C,0,-0.3310775435,-0.1028304881,-0.8553646128

H,0,-2.385907605,-0.7049914932,-2.5402510734

H,0,-4.7931175366,-0.3861396289,-2.0767507912

C,0,0.6055427354,0.3047560133,0.08243146

C,0,0.1752013648,0.8823866463,1.3085993254

H,0,-0.010424648,-0.5081197156,-1.8151664848

H,0,0.917177407,1.2138914512,2.0266045879

S,0,2.3709664349,-0.0977191558,-0.1051048532

O,0,2.1513960302,-1.7282992596,-0.1603328129

C,0,2.7070993261,0.1608922491,-1.8889865198

H,0,2.3362225995,-0.7132702886,-2.4290005227

H,0,2.2723142652,1.1051670545,-2.212721378

H,0,3.7946522588,0.216509173,-1.9785148868

O,0,2.4496070129,2.0257795485,-0.1447696161

C,0,3.1429918649,2.5607928552,0.931335604

H,0,3.7853582233,3.4012783187,0.6097944267

H,0,2.4774708568,2.9617595425,1.7275542377

H,0,3.8111418056,1.8181173313,1.4208732072

Li,0,0.4960521226,-2.1407543922,0.029676265

**TS1** with Potassium:

Zero-point correction= 0.219841 (Hartree/Particle)

Thermal correction to Energy= 0.236198

Thermal correction to Enthalpy= 0.237142

Thermal correction to Gibbs Free Energy= 0.175398

Sum of electronic and zero-point Energies= -1613.403705

Sum of electronic and thermal Energies= -1613.387348

Sum of electronic and thermal Enthalpies= -1613.386404

Sum of electronic and thermal Free Energies= -1613.448148

Electronic Energy = -1613.317

C,0,-4.2580472599,0.470745623,0.0000758608

C,0,-3.3199592037,0.5564565774,1.0107312132

C,0,-1.9299198604,0.4932498825,0.7300820394

C,0,-1.4862794091,0.340861156,-0.6327577108

C,0,-2.4848866742,0.2598028155,-1.6526048002

C,0,-3.8278456633,0.3221957571,-1.3438881019

H,0,-1.2480270855,0.7075397736,2.7826592939

H,0,-5.3189325377,0.5286603173,0.227819739

H,0,-3.63778151,0.6859678287,2.0441338364

C,0,-0.9320157998,0.577531095,1.7483647885

C,0,-0.0990777374,0.2389689894,-0.9159189873

H,0,-2.1650995827,0.1625484801,-2.6883194867

H,0,-4.5668585223,0.2673563411,-2.1399314179

C,0,0.8591793329,0.3687788499,0.0969008558

C,0,0.4102733715,0.5014792199,1.4525392239

H,0,0.2110080918,0.1269929628,-1.9512378007

H,0,1.1488353479,0.572566547,2.2470323778

S,0,2.5601210215,-0.6774329742,-0.0137213152

O,0,2.0831925576,-2.1664211822,0.0284198535

C,0,2.9978554858,-0.3960838628,-1.7599517103

H,0,2.2055286385,-0.7966919252,-2.3984850501

H,0,3.1274214413,0.6788879576,-1.8950460664

H,0,3.9238000494,-0.9497667439,-1.93910556

O,0,2.1821518789,1.9212414044,-0.3105346113

C,0,1.9904765474,3.0016527249,0.5378602247

H,0,2.377354957,3.9264169921,0.069825616

H,0,0.9196089675,3.1898002105,0.7617400779

H,0,2.5106024327,2.8974574452,1.5139102002

K,0,-0.4049046756,-2.5172482124,0.4564591678

**TS1** with Sodium:

Zero-point correction= 0.220376 (Hartree/Particle)

Thermal correction to Energy= 0.236509

Thermal correction to Enthalpy= 0.237454

Thermal correction to Gibbs Free Energy= 0.176618

Sum of electronic and zero-point Energies= -1175.788709

Sum of electronic and thermal Energies= -1175.772576

Sum of electronic and thermal Enthalpies= -1175.771632

Sum of electronic and thermal Free Energies= -1175.832467

Electronic Energy = -1175.688

C,0,-4.3029144292,-0.5099703442,0.0828273765

C,0,-3.3771237043,-0.6669542256,-0.9290960518

C,0,-1.9868493658,-0.539220097,-0.672207102

C,0,-1.5410293621,-0.2381518677,0.6597964079

C,0,-2.5230220502,-0.082958824,1.6815125759

C,0,-3.8667058461,-0.2159135033,1.3995384914

H,0,-1.3247218544,-0.9126471039,-2.7059975387

H,0,-5.3642297777,-0.6174901757,-0.1239689351

H,0,-3.7028643935,-0.9013548101,-1.940683078

C,0,-1.0005180596,-0.6890790319,-1.6913191009

C,0,-0.14909664,-0.0819478338,0.9145374846

H,0,-2.1935851109,0.1306700278,2.6962413746

H,0,-4.5997033834,-0.1020068305,2.194395844

C,0,0.7997679374,-0.3088644792,-0.0843719249

C,0,0.3427823949,-0.5647794499,-1.4189854931

H,0,0.165562276,0.1368481398,1.931026255

H,0,1.0750058431,-0.6975908469,-2.2105668084

S,0,2.5086215231,0.2721381845,0.0337850659

O,0,2.3217710889,1.8448757096,-0.1471232163

C,0,2.8648266345,0.1656302457,1.8233157482

H,0,2.2515898848,0.9068167572,2.3422487576

H,0,2.6963615916,-0.8568172737,2.1580912456

H,0,3.9172199319,0.4450107743,1.9223057311

O,0,2.1554444208,-2.0223555036,0.3948842322

C,0,2.3592091838,-2.9017959931,-0.6534746686

H,0,2.6742033241,-3.8948637015,-0.2789254075

H,0,1.4437340238,-3.0792259245,-1.2612875127

H,0,3.1499103343,-2.5643503012,-1.3601151229

Na,0,0.2471148041,2.2569087126,-0.4957469597

**TS1** with Lithium:

Zero-point correction= 0.222428 (Hartree/Particle)

Thermal correction to Energy= 0.238057

Thermal correction to Enthalpy= 0.239002

Thermal correction to Gibbs Free Energy= 0.178827

Sum of electronic and zero-point Energies= -1021.024309

Sum of electronic and thermal Energies= -1021.008680

Sum of electronic and thermal Enthalpies= -1021.007736

Sum of electronic and thermal Free Energies= -1021.067911

Electronic Energy = -1020.9623

C,0,-4.3628553233,-0.6204399864,0.2495860595

C,0,-3.543883676,-0.3556498377,-0.8276872188

C,0,-2.1306250761,-0.3204703659,-0.6762033105

C,0,-1.5637856248,-0.5677964018,0.6174588237

C,0,-2.4364312454,-0.8416864666,1.7077042206

C,0,-3.8032001257,-0.8663027317,1.5295906929

H,0,-1.6685198698,0.0845007652,-2.7564877401

H,0,-5.4419249251,-0.6457104613,0.1205205516

H,0,-3.9712801394,-0.1722073688,-1.8114643667

C,0,-1.2500672903,-0.0636656611,-1.7626554555

C,0,-0.1503430747,-0.5475545843,0.7765681459

H,0,-2.0044483596,-1.0366366477,2.6866327785

H,0,-4.4582281529,-1.0794515434,2.3704920687

C,0,0.6701910602,-0.2511814841,-0.2870214451

C,0,0.1149675896,-0.0179122518,-1.5732683708

H,0,0.2815348774,-0.803597364,1.7379139129

H,0,0.7789260625,0.1607231489,-2.4148144988

S,0,2.4908425894,-0.138183112,-0.1357096388

O,0,2.5676458978,1.3787498334,-0.8454743812

C,0,2.7665805523,0.5287303719,1.5480270631

H,0,2.0774150737,1.3633473423,1.6912539761

H,0,2.6458901286,-0.2639800396,2.2836953833

H,0,3.7920666822,0.9051224282,1.54333808

O,0,2.3966741603,-1.8984106421,0.8845387106

C,0,2.3631333148,-2.9871067247,0.0196097168

H,0,1.335386612,-3.251028529,-0.3060816068

H,0,2.9587699436,-2.8120503517,-0.903036804

H,0,2.7894548303,-3.8777847016,0.5104594981

Li,0,1.9557581881,2.6596390373,-1.6808127551

***Mechanism B:***

**TS1_B_** with Potassium:

Zero-point correction= 0.219511 (Hartree/Particle)

Thermal correction to Energy= 0.235909

Thermal correction to Enthalpy= 0.236853

Thermal correction to Gibbs Free Energy= 0.174840

Sum of electronic and zero-point Energies= -1613.414502

Sum of electronic and thermal Energies= -1613.398104

Sum of electronic and thermal Enthalpies= -1613.397160

Sum of electronic and thermal Free Energies= -1613.459174

C,0,-4.69858903,0.3269138386,0.3236168702

C,0,-3.6489743823,0.642024464,1.1683314776

C,0,-2.3093444706,0.3555057085,0.8144136047

C,0,-2.0186140519,-0.2745585158,-0.4455811975

C,0,-3.1253783689,-0.5908313834,-1.2914141319

C,0,-4.4219528513,-0.2984272562,-0.9175457863

H,0,-1.4087588661,1.1001337418,2.6449822376

H,0,-5.7235378775,0.552335325,0.6064142906

H,0,-3.8434090466,1.1190539484,2.128369772

C,0,-1.2066788503,0.6477844411,1.6741698718

C,0,-0.6818252125,-0.5698411962,-0.8017377826

H,0,-2.9293250041,-1.0714071735,-2.2484985067

H,0,-5.2432327167,-0.5512777795,-1.5853038009

C,0,0.4181970763,-0.2044917388,0.0248996731

C,0,0.0857172475,0.3644152458,1.3236116401

H,0,-0.4845928714,-1.0526169195,-1.7576036902

H,0,0.899738372,0.5834306576,2.0132873453

S,0,1.6954560719,-1.6731387794,0.0383647424

O,0,3.047603807,-1.2807286244,0.7185713742

O,0,1.5243896638,0.8329340094,-0.7086393075

C,0,0.8523581347,1.9082876365,-1.3414554366

H,0,1.5921131167,2.4910678764,-1.9090718975

H,0,0.086681349,1.5499895766,-2.0426746409

H,0,0.3633674017,2.5716731295,-0.6107719856

K,0,3.941658405,0.9757769642,0.1557074538

C,0,0.8633578956,-2.6763735925,1.3111976847

H,0,-0.1457397686,-2.9200040543,0.97152379

H,0,1.4577371121,-3.5847844364,1.4384459057

H,0,0.8253200554,-2.1083832536,2.2453751804

**TS1_B_** with Sodium:

Zero-point correction= 0.220426 (Hartree/Particle)

Thermal correction to Energy= 0.236440

Thermal correction to Enthalpy= 0.237384

Thermal correction to Gibbs Free Energy= 0.176975

Sum of electronic and zero-point Energies= -1175.805234

Sum of electronic and thermal Energies= -1175.789220

Sum of electronic and thermal Enthalpies= -1175.788275

Sum of electronic and thermal Free Energies= -1175.848685

C,0,4.7318970934,-0.2046646749,0.4262393307

C,0,3.6667070306,-0.2522846663,1.3072728898

C,0,2.3308181315,-0.1556601354,0.849703756

C,0,2.0632225778,-0.0051627951,-0.5550545328

C,0,3.1854357323,0.0442867171,-1.4367207462

C,0,4.4771543024,-0.0531192604,-0.9595745221

H,0,1.3929916078,-0.2803211284,2.8043031067

H,0,5.7534862626,-0.2808186921,0.7891280295

H,0,3.8444831216,-0.3655397396,2.3760212901

C,0,1.2104308991,-0.1854493586,1.7342284365

C,0,0.730071065,0.0953627209,-1.0188619895

H,0,3.005683821,0.1602493563,-2.5041134416

H,0,5.3116323296,-0.0133159528,-1.6567524338

C,0,-0.3824083714,0.0064396871,-0.1376638751

C,0,-0.0784804189,-0.0944654294,1.2813298659

H,0,0.5491685979,0.2145311885,-2.0856708298

H,0,-0.9078629279,-0.1108635425,1.9871974204

S,0,-1.6652772775,1.4317196865,-0.6424330869

O,0,-3.0750639638,1.2497671908,0.0341156197

O,0,-1.466372065,-1.1913947668,-0.515457058

C,0,-0.8036595838,-2.4364179896,-0.7045551125

H,0,-1.5429902154,-3.1620138105,-1.0670419565

H,0,-0.0041037678,-2.3475539033,-1.4502041405

H,0,-0.3653528039,-2.8069371073,0.2332894711

C,0,-0.9058589582,2.7623974116,0.3374908276

H,0,0.1292837497,2.8938365292,0.0140465979

H,0,-1.4858087491,3.6692400249,0.1489641847

H,0,-0.9439171344,2.4953442323,1.3975507875

Na,0,-3.5359090854,-0.8384927923,0.0893521114

**TS1_B_** with Lithium:

Zero-point correction= 0.221988 (Hartree/Particle)

Thermal correction to Energy= 0.237410

Thermal correction to Enthalpy= 0.238354

Thermal correction to Gibbs Free Energy= 0.179820

Sum of electronic and zero-point Energies= -1021.052739

Sum of electronic and thermal Energies= -1021.037317

Sum of electronic and thermal Enthalpies= -1021.036373

Sum of electronic and thermal Free Energies= -1021.094907

C,0,4.7691783643,-0.1753811704,0.4133722755

C,0,3.7057228752,-0.2098645426,1.2953810885

C,0,2.3682016819,-0.1408671676,0.8333937316

C,0,2.1037255646,-0.0337650607,-0.5747862935

C,0,3.2240339618,0.0016593913,-1.4580937891

C,0,4.5157273912,-0.067682678,-0.9770371783

H,0,1.4270885468,-0.2463487452,2.7870624463

H,0,5.7912383377,-0.2293661391,0.7787084378

H,0,3.8840643454,-0.2909588268,2.3666494997

C,0,1.2480663765,-0.1660665008,1.7155627167

C,0,0.7685010458,0.0407712284,-1.043569547

H,0,3.0434632608,0.0852742541,-2.5281849936

H,0,5.3510240948,-0.0392172794,-1.6734444982

C,0,-0.3318209017,-0.0042904496,-0.1590996235

C,0,-0.0417796424,-0.0984845155,1.2552687307

H,0,0.588329997,0.1348698256,-2.1126929877

H,0,-0.874186254,-0.1283338428,1.9563039696

S,0,-1.642575359,1.4794143821,-0.6898937872

O,0,-3.0972375679,1.1703371628,-0.1373984224

O,0,-1.4910868092,-1.1494694273,-0.5301236799

C,0,-0.9116947346,-2.4461277784,-0.6476231855

H,0,-1.682721542,-3.1287732259,-1.0234157413

H,0,-0.0745162123,-2.430187078,-1.3552088384

H,0,-0.5463425736,-2.808698251,0.3224550091

C,0,-1.0138106599,2.7463553039,0.4472829248

H,0,0.0368608699,2.9315169462,0.2080315159

H,0,-1.6056926665,3.6516455636,0.2917821464

H,0,-1.1111973445,2.3957517798,1.4784727208

Li,0,-3.181163446,-0.6050141587,-0.1630246479
